# Extending the low-temperature operation of sodium metal batteries combining linear and cyclic ether-based electrolyte solutions

**DOI:** 10.1038/s41467-022-32606-4

**Published:** 2022-08-22

**Authors:** Chuanlong Wang, Akila C. Thenuwara, Jianmin Luo, Pralav P. Shetty, Matthew T. McDowell, Haoyu Zhu, Sergio Posada-Pérez, Hui Xiong, Geoffroy Hautier, Weiyang Li

**Affiliations:** 1 Thayer School of Engineering, Dartmouth College, 14 Engineering Drive, Hanover, NH 03755 USA; 2 G.W. Woodruff School of Mechanical Engineering, Georgia Institute of Technology, 801 Ferst Drive, Atlanta, GA 30332 USA; 3 School of Materials Science and Engineering, Georgia Institute of Technology, 771 Ferst Drive, Atlanta, GA 30332 USA; 4 Micron School of Materials Science and Engineering, Boise State University, 1910 University Drive, Boise, ID 83725 USA; 5 Institute of Condensed Matter and Nanosciences, UCLouvain, Chemin des Étoiles 8, B-1348 Louvain-la-Neuve, Belgium; 6 Institut de Química Computacional i Catàlisi and Departament de Química, Universitat de Girona, C/ Maria Aurèlia Capmany, 69, 17003 Girona, Catalonia Spain; 7 Center for Advanced Energy Studies, Idaho Falls, ID 83401 USA

**Keywords:** Energy science and technology, Batteries, Materials for energy and catalysis, Electrochemistry, Energy

## Abstract

Nonaqueous sodium-based batteries are ideal candidates for the next generation of electrochemical energy storage devices. However, despite the promising performance at ambient temperature, their low-temperature (e.g., < 0 °C) operation is detrimentally affected by the increase in the electrolyte resistance and solid electrolyte interphase (SEI) instability. Here, to circumvent these issues, we propose specific electrolyte formulations comprising linear and cyclic ether-based solvents and sodium trifluoromethanesulfonate salt that are thermally stable down to −150 °C and enable the formation of a stable SEI at low temperatures. When tested in the Na||Na coin cell configuration, the low-temperature electrolytes enable long-term cycling down to −80 °C. Via ex situ physicochemical (e.g., X-ray photoelectron spectroscopy, cryogenic transmission electron microscopy and atomic force microscopy) electrode measurements and density functional theory calculations, we investigate the mechanisms responsible for efficient low-temperature electrochemical performance. We also report the assembly and testing between −20 °C and −60 °C of full Na||Na_3_V_2_(PO_4_)_3_ coin cells. The cell tested at −40 °C shows an initial discharge capacity of 68 mAh g^−1^ with a capacity retention of approximately 94% after 100 cycles at 22 mA g^−1^.

## Abstract (web summary)

The low-temperature operation of non-aqueous sodium-based batteries is affected by the properties of the electrolyte. Here the authors propose specific electrolyte formulations that are thermally stable down to −150 °C and enable a stable electrode|electrolyte interface at low temperatures.

## Introduction

Lithium-ion batteries (LIBs) have been extensively employed in portable electronics and electric vehicles because of their high energy density and long cycle life^1–3^. Nevertheless, they inevitably suffer from severe energy/power losses in cold environments, especially when the temperature drops below −20 °C^4,5^. Such poor low-temperature performance limits their application in aeronautics/space missions, polar expeditions and many military and civil facilities in cold regions, in which a battery operating temperature below −40 °C is required^4,6^.

Searching for a system with appealing electrochemical energy storage features beyond Li-based technologies would be promising for addressing the challenges associated with low-temperature operation. As an alkali metal, sodium (Na) stands out because it shares many chemical and physical properties with Li while being substantially more naturally abundant^7–9^. Na has a lower first ionization energy than Li (495.8 vs. 520.2 kJ mol^−1^)^10^, which may contribute to improved chemical/electrochemical reactivity and facilitate electrochemical reactions in cold environments. Na metal plays a crucial role as an anode material for Na batteries because of its low electrode potential (−2.714 V vs. standard hydrogen electrode) and high theoretical specific capacity (1166 mAh g^−1^)^7–9,11–14^. Nevertheless, investigation of Na batteries at low temperatures has been limited, and in particular, an understanding of the behavior of Na metal as an electrode is largely lacking^15–18^.

The enabling of low-temperature battery operation highly depends on the nature of the electrolyte^19–22^. The electrolyte resistance rapidly increases as the temperature drops because of the relatively high freezing/melting points of nonaqueous carbonate solvents and the reduced solubility of conducting salts^5,19^. Moreover, the solid electrolyte interphase (SEI) formed at ambient temperature might not be able to maintain the same protective capabilities under cold conditions to enable efficient cycling. Meanwhile, the low-temperature structural and compositional evolution of the SEI formed on the Na metal electrode still remains elusive.

One viable solution to circumvent these problems is to formulate electrolytes targeted at low-temperature operation using solvents with low melting points and salts capable of forming a stable SEI. Here, applying such an electrolyte strategy, we demonstrate that an electrolyte solution comprising an acyclic ether solvent and a compatible Na salt can extend the Na metal operating temperature to −40 °C. The trifluoromethanesulfonate (OTf) salt is found to play a critical role in enabling the formation of a stable SEI at low temperatures. Furthermore, adding a cyclic ether solvent to prepare a binary-solvent electrolyte solution can expand the thermostability temperature threshold down to −150 °C. We demonstrate stable Na metal plating/stripping in symmetric cells at low temperatures down to −80 °C, exhibiting a low overpotential of ~150 mV for over 750 h. This performance expands the low-temperature operational capability of alkali metal electrodes in nonaqueous electrolyte solutions (see Supplementary Fig. 1 and Supplementary Table 1 for a comparison against the state of the art). Coupled experimental characterizations (e.g., X-ray photoelectron spectroscopy, cryogenic transmission electron microscopy, and atomic force microscopy) and density functional theory calculations allow an understanding of the mechanistic features that enable efficient low-temperature electrochemical performance. Full Na||Na_3_V_2_(PO_4_)_3_ coin cells are also assembled and are tested between −20 °C and −60 °C. The cells tested at −40 °C and −60 °C show initial discharge capacities of ~68 and 39 mAh g^−1^, respectively, with capacity retentions of ~94% and 91% after 100 cycles at 22 mA g^−1^.

## Results

### Screening of single-solvent electrolytes at low temperatures

We screened different electrolyte solutions at −35 °C using a variety of salt-solvent combinations. Ethers with low melting points, including diethylene glycol dimethyl ether (DEGDME), 1,2-dimethoxyethane (DME) and 1,3-dioxolane (DOL), were chosen as candidate solvents (Supplementary Table 2). A variety of conducting salts, including sodium hexafluorophosphate (NaPF_6_), sodium trifluoromethanesulfonate (NaOTf), sodium perchlorate (NaClO_4_), sodium bis(trifluoromethanesulfonyl)imide (NaTFSI) and sodium bis(fluorosulfonyl)imide (NaFSI), were investigated as candidate salts. The salt concentration in the solvent was kept at 1 M for the solubility screening of these single-solvent electrolytes at low temperatures. The results (Supplementary Table 3) suggest that the solubility of NaPF_6_ cannot reach a 1 M concentration in all three solvents at −35 °C, while the other four salts present good solubility in DEGDME. Therefore, while 1 M NaPF_6_-DEGDME has been shown to be a suitable electrolyte for the Na metal anode under ambient conditions^23^, it may not be suitable for low-temperature operation. Moreover, DOL cannot easily dissolve Na salts except for NaTFSI. The electrolyte consisting of 1 M NaPF_6_-DEGDME was further investigated across a range of temperatures in Na||Na and stainless steel||stainless steel symmetric cells, respectively (Supplementary Fig. 2). A comparison of the electrochemical impedance spectroscopy (EIS) spectra of the 1 M NaPF_6_-DEGDME tested in Na||Na symmetric cells shows that the electrode-electrolyte interfacial resistance at −20 °C is significantly higher than that at 20 °C (Supplementary Table 4). Meanwhile, the total electrolyte resistance measured in the stainless steel||stainless steel cells (cell configuration is shown in Supplementary Fig. 3) increases with decreasing temperature. This increase in electrolyte resistance is especially significant at a temperature below −20 °C (Supplementary Table 5), which could be mainly due to the precipitation of NaPF_6_ in DEGDME at low temperatures. In summary, eight of the fifteen single-solvent electrolytes are thermally stable at −35 °C at 1 M concentrations, and these were chosen for further electrochemical evaluation with metallic Na.

### Electrochemical behavior of single-solvent electrolytes

**Fig. 1:**
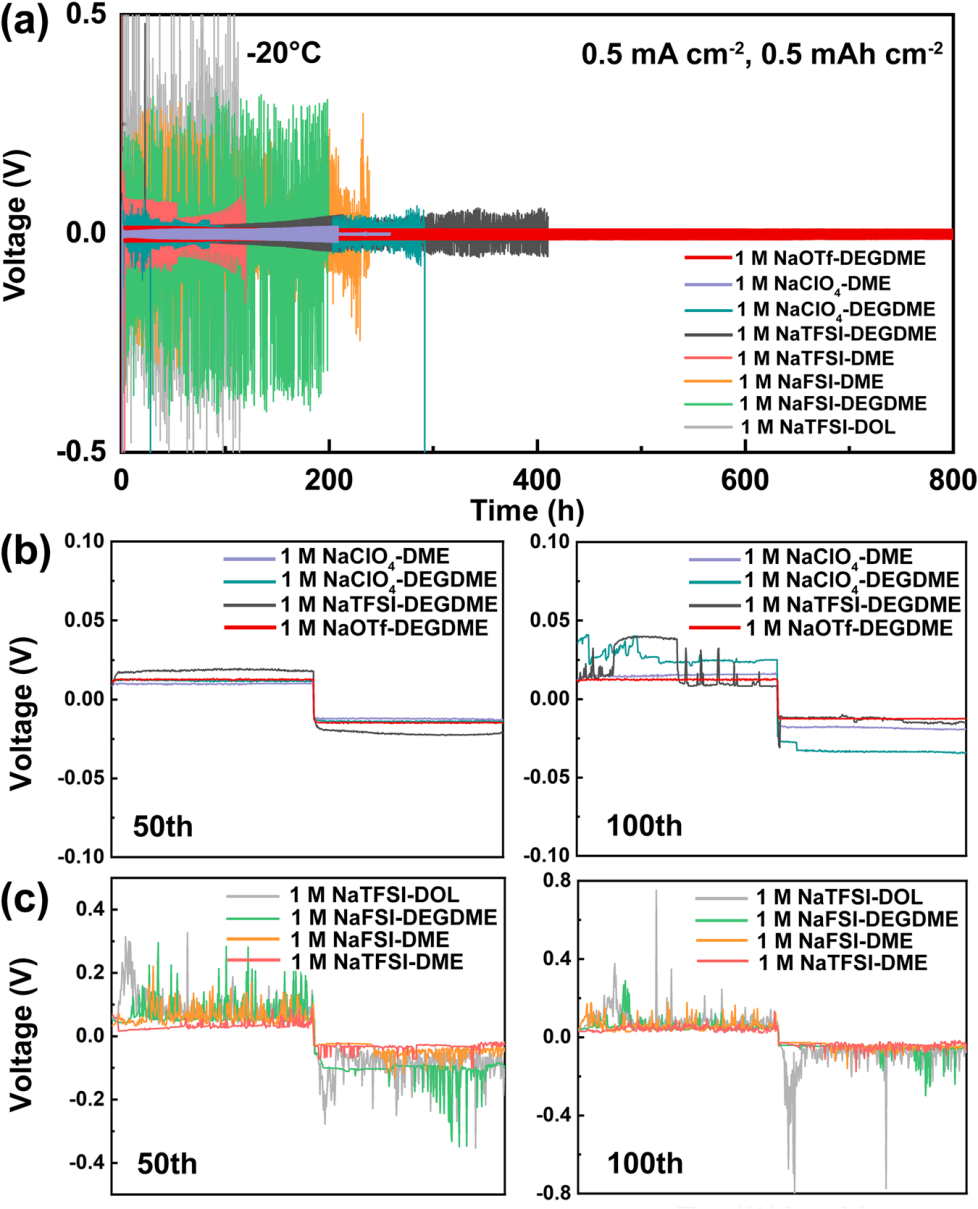
Electrochemical cycling of Na metal electrodes in various electrolyte formulations at −20 °C. **a** Galvanostatic cycling of Na||Na symmetric cells operating in eight different electrolyte formulations with a 1 M salt concentration at 0.5 mA cm^−2^ and 0.5 mAh cm^−2^. **b**, **c** Corresponding enlarged voltage profiles at the 50th cycle and 100th cycle for **b** four of the formulations and **c** the remaining four formulations.

Galvanostatic cycling measurements were performed in symmetric Na||Na cells at a current density of 0.5 mA cm^−2^ with a cycling capacity of 0.5 mAh cm^−2^ at −20 °C (Fig. 1a–c) and +20 °C (Supplementary Figs. 4 and 5). Among the eight electrolyte candidates, the cell with 1 M NaOTf-DEGDME presents the most stable cycling with the smallest average overpotential (<10 mV) for 600 h (300 cycles) at +20 °C (the electrochemical behavior for different electrolytes at +20 °C is summarized in Supplementary Table 6). The electrolytes with NaTFSI salt all show much higher overpotentials than the other electrolytes. Specifically, the cell with NaTFSI-DOL shows an increasing overpotential that rapidly reaches 1 V, and the cells with 1 M NaTFSI-DEGDME and 1 M NaTFSI-DME failed quickly, reaching the protection voltage (5 V) at 32 and 44 h, respectively.

**Fig. 2:**
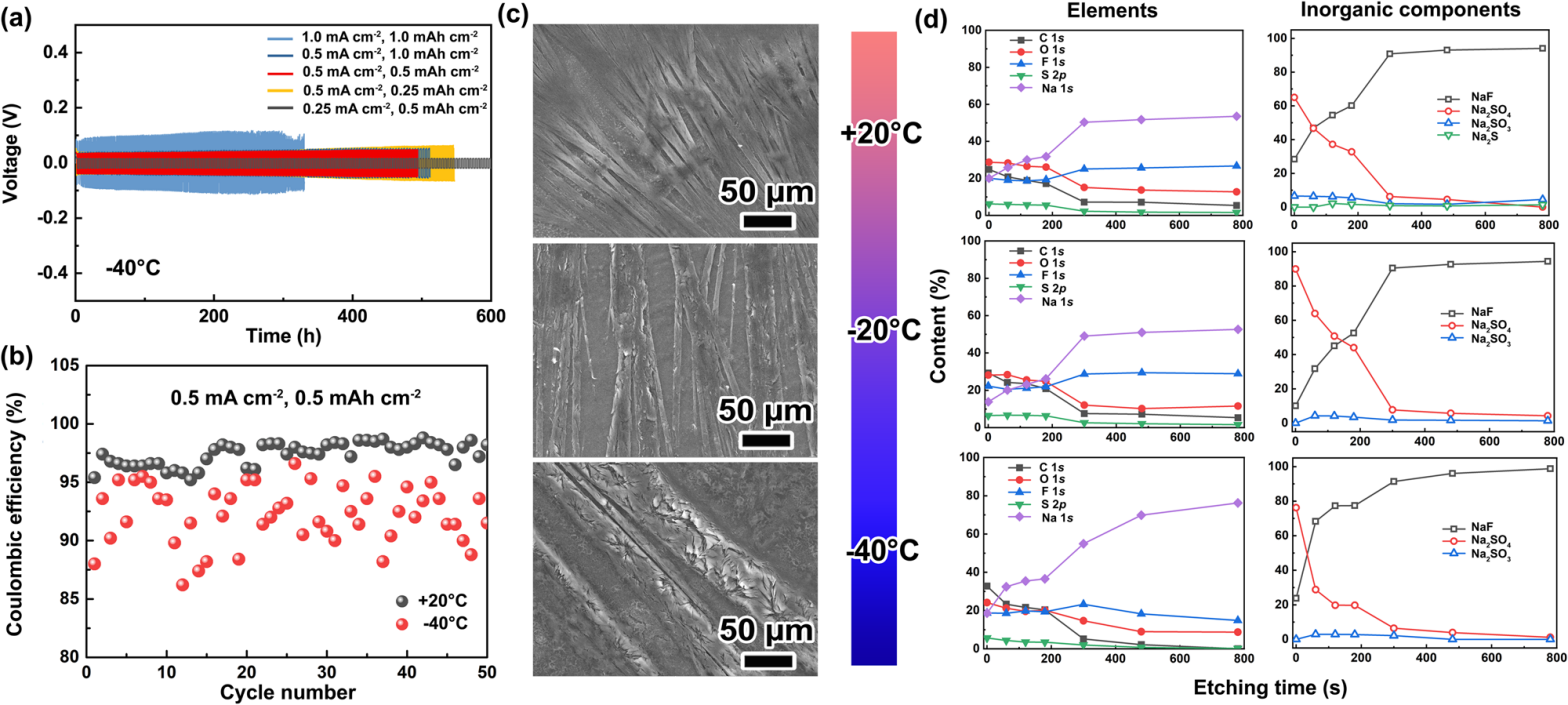
Electrochemical and physicochemical characterizations of Na metal electrodes cycled in 1 M NaOTf-DEGDME at various temperatures. **a** Galvanostatic cycling of Na||Na symmetric cells at current densities up to 1.0 mA cm^−2^ with capacities up to 1.0 mAh cm^−2^ at −40 °C. **b** CE as a function of cycle number for Na||stainless steel cells at 0.5 mA cm^−2^ and 0.5 mAh cm^−2^ at +20 °C and −40 °C. **c** Ex situ postmortem SEM measurements of Na metal electrodes after 50 cycles at 0.5 mA cm^−2^ and 0.5 mAh cm^−2^ at +20 °C, −20 °C and −40 °C. **d** Contents of elements (left column) and inorganic components (right column) determined by ex situ postmortem XPS depth profiling of the Na metal electrodes shown in Fig. 2c.

When tested at −20 °C, the cell with 1 M NaOTf-DEGDME still displays the highest stability, with smooth and steady voltage plateaus as well as the lowest overpotential of ~16 mV (Fig. 1a, b). In comparison, unstable and fluctuating voltage spikes are observed for the other electrolyte formulations (Fig. 1b, c). Because of the better performance of NaOTf-DEGDME at both +20 °C and −20 °C, it was selected for further testing in symmetric cells at −40 °C (Fig. 2a). At such a low temperature, the initial overpotential of 40 mV only rises to 50 mV even after 500 h of cycling at 0.5 mA cm^−2^ (capacity: 1 mAh cm^−2^), corresponding to a less than 0.2% increase per cycle. Even at a current density of 1 mA cm^−2^, an average overpotential of 100 mV can be maintained for over 300 h. Regarding the coulombic efficiency (CE) evaluation (Fig. 2b), the low average value of the CE at −40 °C compared to that at +20 °C suggests that a significant Na inventory loss occurs at −40 °C.

### Physicochemical characterization of single-solvent electrolytes

The morphology of the Na metal surface after Na plating/stripping in different electrolytes was characterized using scanning electron microscopy (SEM). NaTFSI, NaClO_4_ and NaFSI salts all result in irregular surfaces (pores and/or cracks) of the deposited Na (Supplementary Figs. 6–9). In contrast, NaOTf-DEGDME enables relatively smooth surfaces at both +20 °C and −20 °C, where an inhomogeneous texture can be observed on the surface and within the cross-section. This microstructure becomes more distinct at −40 °C (Fig. 2c).

The chemical composition of the SEI formed on the Na metal electrode in NaOTf-DEGDME was revealed by X-ray photoelectron spectroscopy (XPS), as shown in Supplementary Fig. 10. The binding energies of all elements were calibrated with respect to the C 1*s* signal at 284.8 eV. In the C 1*s* XPS profile, the peak with a binding energy of 293.4 eV is assigned to –CF_3_, and the peaks at 288.7 eV and 286.6 eV are ascribed to O–C=O and C–O–C, respectively^24–29^. In the O 1*s* spectrum, the peaks at 533.3 eV and 531.2 eV correspond to CH_3_–O–/–CH_2_–O– groups^30^ and C–O–Na (e.g., RCH_2_ONa)^31^, respectively, and the peak at 536.3 eV is assigned to Na KLL. For S 2*p*, the doublets at 169.6 eV and 167.2 eV (based on 2*p*_3/2_) are assigned to SO_4_^2−^ and SO_3_^2−^, respectively. For F 1*s*, the peaks at 689.3 eV and 684.1 eV are assigned to C–F and NaF, respectively^24–29^. These XPS spectra at different temperatures reveal similar compositions of the SEI surface, suggesting the existence of –CF_3_-containing compounds, Na_2_SO_4_, Na_2_SO_3_, Na_2_CO_3_, NaF, C–O–Na and organic debris. Na_2_SO_3_ can only be detected on the surface at +20 °C. In addition, Na_2_S (S^2−^: 161.1 eV) cannot be detected in the inner SEI at −20 °C and −40 °C (Supplementary Figs. 11–13). The absence of species with lower oxidation states at low temperatures suggests that the parasitic reactions between the electrolyte and Na metal electrode are suppressed. Importantly, the SEI composition formed in NaOTf-DEGDME shows high consistency across these temperatures. For the other systems tested, the discussion of the Na surface morphology (Supplementary Figs. 6–9) and chemical composition of the SEI (Supplementary Figs. 14–22, Supplementary Tables 7 and 8) is detailed in Supplementary Notes 1 and 2, respectively.

The distribution of chemical species within the SEI formed in 1 M NaOTf-DEGDME at different temperatures was further analyzed by XPS depth profiling (Fig. 2d). At all temperatures, the O and C contents decrease with increasing depth, revealing that the organic debris is mainly present in the upper layer of the SEI. For inorganic components, NaF and Na_2_SO_4_ are the two major species. The NaF/Na_2_SO_4_ ratio increases with increasing depth, suggesting that NaF dominates the inner part of the SEI. At −40 °C, the Na content exhibits a rapid increase with increasing etching time due to the formation of a thinner SEI layer at a lower temperature.

### Theoretical investigation on single-solvent electrolytes

**Fig. 3:**
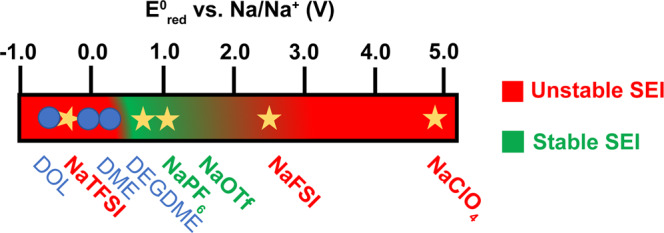
Schematic of the calculated reduction potentials of salts (stars) and solvents (dots). The green and red regions show the optimum and poor salt reduction potentials for building a uniform SEI.

To help rationalize the performance of different salts, we performed first-principles computations within density functional theory (DFT) to evaluate their reduction potentials. Figure 3 shows the computed reduction potentials for the different salts and compares them to the SEI quality. Supplementary Table 9 collects the calculated reduction potentials of salts and solvents with or without the Na cation. Salts such as NaTFSI, which have a low reduction potential close to the electrolyte reduction potential, do not allow fast formation of an inorganic-compound-rich SEI capable of preventing electrolyte decomposition. This is well consistent with the Na stripping/plating behavior in all NaTFSI-based electrolytes, which show high overpotentials (Supplementary Table 6). In contrast, salts with high reduction potentials such as NaFSI and NaClO_4_ (2.37 V and 4.96 V vs. Na/Na^+^, respectively) promptly react with Na metal to form an inorganic SEI layer, but the strong driving force for the reaction leads to the formation of SEIs with a nonuniform morphology (Supplementary Figs. 6 and 7). NaOTf exhibits an intermediate reduction potential (1.02 V vs. Na/Na^+^), leading to the formation of an inorganic protective layer before the electrolyte is decomposed, but with a moderate driving force, leading to a mild reaction and formation of a smooth SEI film (Fig. 2c). For the sake of comparison, we also provide the results for NaPF_6_, which is known to form a stable SEI at room temperature^23^. NaPF_6_ also exhibits a moderate reduction potential (0.75 V vs. Na/Na^+^), confirming our speculation that a salt with an intermediate reduction potential helps the formation of a uniform SEI on the Na metal electrode surface. Note that NaPF_6_ may not be a suitable low-temperature salt because of solubility issues and the fact that the performance of NaOTf comes from the combination of a low-temperature solubility and a moderate reduction potential.

### Design of binary-solvent electrolytes for temperatures below −40 °C

Motivated by the electrochemical behavior of the NaOTf-DEGDME electrolyte at low temperatures, this electrolyte solution was selected for further formulation of electrolytes that can operate below −40 °C. DOL, which is a cyclic ether with a melting point of −95 °C, was introduced into DEGDME to produce binary-solvent electrolytes (Supplementary Fig. 23) to further improve the low-temperature properties of the electrolyte. However, the Na^+^ solvation ability of DOL is lower than that of DEGDME^32^. Therefore, the solvent volume ratio (DEGDME:DOL = 8:2, 5:5 or 2:8 volume fraction) and salt concentration (0.5 M or 1 M) were evaluated in the screening of binary-solvent electrolytes (Supplementary Table 10).

**Fig. 4:**
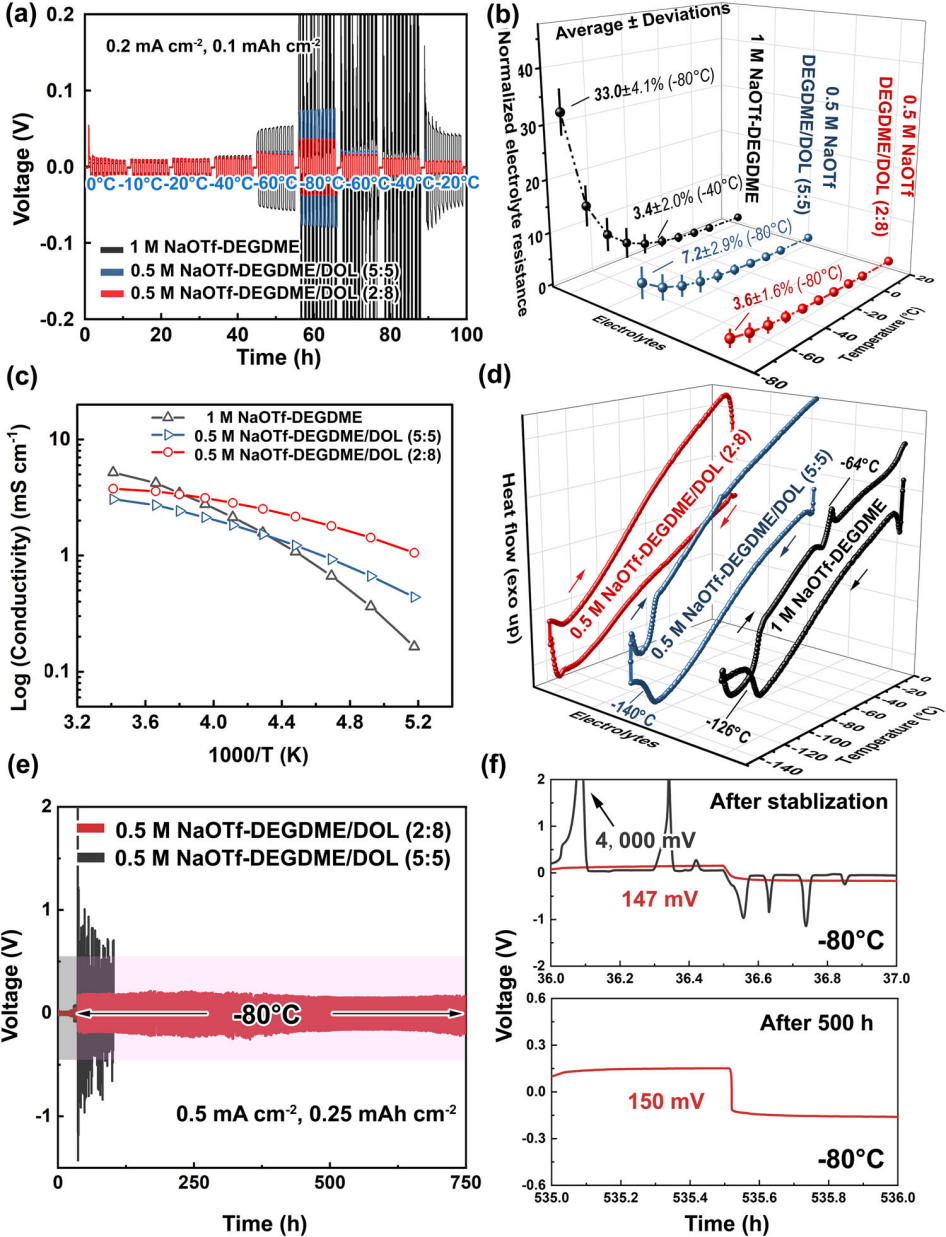
Electrochemical and physicochemical characterizations of different NaOTf-based electrolyte solutions at various temperatures. **a** Temperature-dependent galvanostatic cycling of Na||Na symmetric cells at 0.2 mA cm^−2^ and 0.1 mAh cm^−2^. **b** Change in the resistance of the electrolyte solutions in the temperature range between +20 °C and −80 °C. The resistance values at different temperatures are normalized to those at +20 °C (the value of each electrolyte solution at +20 °C is given as 1). The averages/deviations were calculated based on three consecutive measurements. **c** Temperature-dependent ionic conductivity of the electrolyte solutions in the range between +20 °C and −80 °C. **d** DSC thermograms from 0 °C to −150 °C. **e** Galvanostatic cycling of Na||Na symmetric cells at 0.5 mA cm^−2^ and 0.25 mAh cm^−2^ at −80 °C (pink zone). Note that an initial stepwise temperature drop (gray zone) was applied to stabilize the cells. **f** Enlarged voltage profiles of Fig. 4e after stabilization and after 500 h at −80 °C.

The introduction of DOL extends the low-temperature operating range of the NaOTf-DEGDME system, in which a higher DOL volume fraction leads to better Na cycling stability in symmetric cells (Fig. 4a and Supplementary Fig. 24). Additionally, a lower salt concentration (0.5 M) can accommodate an increased DOL proportion, further enhancing the cell cycling performance (Fig. 4a). At −80 °C, the symmetric cell overpotential (~35 mV) in 0.5 M NaOTf-DEGDME/DOL (2:8) is less than half that (~75 mV) in 0.5 M NaOTf-DEGDME/DOL (5:5) and six times less than that (>200 mV) in 1 M NaOTf-DEGDME/DOL (5:5). Thus, both the NaOTf salt concentration and DEGDME volume fraction should be carefully chosen to formulate an electrolyte solution that enables good low-temperature electrochemical energy storage performance. Replacing DEGDME with DME leads to an increased overpotential and/or an asymmetric voltage profile (Supplementary Fig. 25), which also occurs with the substitution of NaOTf by NaClO_4_ (Supplementary Fig. 26).

### Physicochemical and electrochemical characterization of binary-solvent electrolyte solutions

To understand the mechanism of the enhanced performance at low temperatures, the electrolyte resistance was investigated at different temperatures (Fig. 4b). The resistance of the electrolyte solution was measured by EIS in stainless steel||stainless steel cells (see Methods for details). The symmetric cell configuration is shown in Supplementary Fig. 3 and the resistance evaluation based on EIS is show in Supplementary Fig. 27. The resistance of the 0.5 M NaOTf-DEGDME/DOL (2:8) electrolyte solution at +20 °C is 11.6 Ohm, and it increases by ~3.6 times when the temperature decreases to −80 °C (Supplementary Table 11). This difference is almost half that for 0.5 M NaOTf-DEGDME/DOL (5:5) (7.0 times from +20 °C to −80 °C) and one ninth of that for 1 M NaOTf-DEGDME (31.7 times from +20 °C to −80 °C) (Supplementary Tables 12 and 13). Moreover, the ionic conductivity of the electrolyte can be calculated based on the resistance of the electrolyte solution (see Methods for calculation details). Figure 4c shows that 0.5 M NaOTf-DEGDME/DOL (2:8) gives the slowest decrease in the ionic conductivity with temperature. At −80 °C, the ionic conductivities of 0.5 M NaOTf-DEGDME/DOL (2:8), 0.5 M NaOTf-DEGDME/DOL (5:5) and 1 M NaOTf-DEGDME are 1.05 mS cm^−1^, 0.44 mS cm^−1^, and 0.16 mS cm^−1^, respectively. The ionic conductivities of the other four single-solvent systems are shown in Supplementary Fig. 28. The small change in the electrolyte resistance and ionic conductivity of NaOTf-DEGDME/DOL (2:8) could be attributed to the relatively low dynamic viscosity of the solvents (Supplementary Fig. 29). Note that both binary-solvent and single-solvent electrolytes show good homogeneity without salt precipitation across temperatures (Supplementary Fig. 30). Differential scanning calorimetry (DSC) further confirms the thermal stability of 0.5 M NaOTf-DEGDME/DOL (2:8), revealing no phase transition even down to −150 °C (Fig. 4d)^33^. Similarly, no phase transition is detected until −140 °C for 0.5 M NaOTf-DEGDME/DOL (5:5). In comparison, 1 M NaOTf-DEGDME displays a second-order phase transition at −126 °C during cooling and a first-order phase transition at −64 °C (melting point of DEGDME) during subsequent heating.

Based on the resistance and thermal behavior of the electrolyte solutions, we carried out prolonged Na||Na symmetric cell cycling at −80 °C (Fig. 4e and Supplementary Fig. 31). The cells using 0.5 M NaOTf-DEGDME/DOL (2:8) display a small overpotential of ~50 mV, which does not increase for over 2000 h at 0.2 mA cm^−2^ with a 0.1 mAh cm^−2^ cycling capacity (Supplementary Fig. 31). Even at a higher current of 0.5 mA cm^−2^ with a higher capacity of 0.25 mAh cm^−2^, stable operation for over 750 h can still be achieved (Fig. 4e). The enlarged voltage profile (Fig. 4f) further suggests that the overpotential at 0.5 mA cm^−2^ is initially 147 mV (after the stabilization process) and slightly increases to 150 mV after 500 h, revealing a smooth voltage profile without any spikes. In comparison, 0.5 M NaOTf-DEGDME/DOL (5:5) shows large spikes (>4000 mV) and an asymmetric voltage profile during cycling.

### Ex situ postmortem physicochemical characterization of sodium metal electrodes

**Fig. 5:**
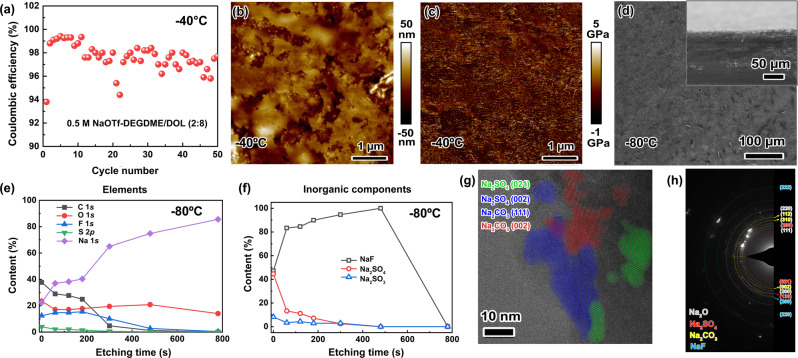
Electrochemical and physicochemical characterizations of Na metal electrodes cycled in 0.5 M NaOTf-DEGDME/DOL (2:8) at low temperatures. **a** CE as a function of cycle number in Na||stainless steel cells at −40 °C at 0.5 mA cm^−2^ and 0.5 mAh cm^−2^. **b** AFM topography of the SEI formed on copper foil after Na plating/stripping at 0.5 mA cm^−2^ (0.5 mAh cm^−2^) with a cutoff voltage of 1.5 V after the 1 st cycle at −40 °C (Cu foil is fully desodiated). **c** Young’s modulus (determined by AFM) of the SEI in Fig. 5b. **d** SEM of the Na metal electrode surface after cycling at −80 °C (Inset: corresponding cross-sectional SEM image). **e** Contents of C 1*s*, O 1*s*, F 1*s*, S 2*p* and Na 1*s* elements in the SEI at −80 °C. **f** Contents of the main SEI inorganic components, including NaF, Na_2_SO_4_ and Na_2_SO_3_, at −80 °C. **g** Cryo-TEM image of the SEI after cycling at −40 °C. Visible lattice fringes from crystalline regions are overlaid with different colors. The red lattice fringes have a d-spacing of 3.0 Å, the blue have a d-spacing of 3.5 Å, and the green have a d-spacing of 3.8 Å. These three fringes likely correspond to the labeled planes shown. **h** SAED pattern of the SEI formed at −40 °C. The positions of the rings expected to be most intense from Na_2_O, Na_2_SO_4_, Na_2_CO_3_, and NaF are labeled; the large variety of crystalline phases likely present makes fully indexing the structures difficult. These crystalline phases also give rise to extra rings that are not marked, which likely account for the reflections that fall between marked lines. Note that the presence of Na_2_O may be attributed to the transfer process for these samples.

An improved CE with an average value of ~97.6% at −40 °C is observed in 0.5 M NaOTf-DEGDME/DOL (2:8) (Fig. 5a) compared to the single-solvent case (~92.2%, Fig. 2b) using Na||stainless steel cells at 0.5 mA cm^−2^ and 0.5 mAh cm^−2^. Ex situ atomic force microscopy (AFM) measurements were carried out to investigate the surface roughness and mechanical properties of the SEI formed on copper foil at 0.5 mA cm^−2^ (0.5 mAh cm^−2^) for the 1st cycle at −40 °C (Fig. 5b, c and Supplementary Fig. 32). In 0.5 M NaOTf-DEGDME/DOL (2:8), the height of the SEI formed at the electrode surface is uniform with a deviation of 50 nm (Fig. 5b), and the average Young’s modulus is ~1.2 GPa (Fig. 5c), which are better than those in 1 M NaOTf-DEGDME (height deviation: 100 nm; Young’s modulus: 0.5 GPa), as shown in Supplementary Fig. 32. The better uniformity and mechanical property of the SEI contributes to more efficient plating/striping behavior on copper foil (Supplementary Fig. 33) in the binary-solvent electrolyte compared to the single-solvent case. The SEI morphology after Na stripping was investigated via SEM (Supplementary Fig. 34), in which a smoother surface when using 0.5 M NaOTf-DEGDME/DOL (2:8) can be noted compared to the irregular and porous morphology obtained using the 1 M NaOTf-DEGDME electrolyte solution.

The binary-solvent system can also alter the composition and microstructure of the Na electrode surface in comparison to the single-solvent system. Specifically, a gradual increase in the volume fraction of DOL results in the lack of formation of irregular morphologies such as those observed in NaOTf-DEGDME at low temperatures (Fig. 5d and Supplementary Figs. 35 and 36). Regarding the composition, ex situ postmortem XPS measurements of the Na metal electrodes cycled using the NaOTf-DEGDME/DOL electrolyte at −80 °C (Supplementary Figs. 37–40) suggest inorganic components (NaF, Na_2_SO_4_, and Na_2_SO_3_) in the SEI similar to those formed in the single-solvent counterpart. Nevertheless, XPS depth profiling analysis reveals a difference in the distribution of elements and inorganic components in binary-solvent electrolytes (Fig. 5e, f and Supplementary Fig. 41) compared to the single-solvent counterpart (Fig. 2d). Specifically, the NaF/Na_2_SO_4_ ratio is higher on the surface (~1:1) when using the binary-solvent electrolytes (Fig. 5f and Supplementary Fig. 41b) than those (1:8.9 at −20 °C and 1:3.3 at −40 °C) in the single-solvent system (Fig. 2d). Taking into account the AFM investigations, assuming that the higher percentage of the NaF inorganic component in the binary-solvent electrolyte contributes to a higher Young’s modulus (Fig. 5c and Supplementary Fig. 32b) and thus to a better mechanical strength compared to the single-solvent system is plausible.

Cryo-TEM further indicates the crystalline nature of the SEI (likely containing Na_2_SO_4_ and Na_2_CO_3_) via identification of lattice fringes and analysis of electron diffraction patterns^34^. Specifically, the SEI growth in the binary-solvent system (Fig. 5g, h) shows a mosaic structure, consisting of an amorphous matrix with Na-rich nanocrystallites dispersed within, similar to that in the single-solvent system (Supplementary Fig. 42). These data are consistent with the XPS experimental findings on the SEI for both cases. However, the crystallite size and overall quantity of crystallites within the SEI are larger in the binary-solvent electrolyte, as shown by the TEM images as well as fast Fourier transform (FFT) and selected area electron diffraction (SAED) patterns (Fig. 5g, h), in which there are more discrete spots for the binary-solvent system compared to the rings in the single-solvent case. In addition, unlike the binary-solvent system, the single-solvent system can hardly reveal the presence of NaF (Supplementary Fig. 42). Since NaF can be detected via XPS of the SEI formed in the single solvent, this suggests that the NaF in the SEI of the single solvent is mainly amorphous or has poor crystallinity. These observations are consistent across several imaged regions of the SEI for both the single-solvent and binary-solvent systems.

Based on XPS and cryo-TEM observations, the binary-solvent electrolyte contributes to a higher content of crystalline NaF in the upper part of the SEI compared to that in the single-solvent system. Additionally, AFM confirms the results by suggesting a higher Young’s modulus of 1.2 GPa (Fig. 5c) in the binary-solvent electrolyte. Moreover, both AFM (Fig. 5b and Supplementary Fig. 32a) and SEM (Supplementary Fig. 34) suggest that the binary-solvent electrolyte can develop an SEI with lower surface roughness (smaller height deviation) compared to that in the single-solvent system. We speculate that the NaF-rich SEI with a uniform morphology and an improved Young’s modulus is the main reason for the long-term stability of the cell performance at −80 °C. This finding is in agreement with a prior report on a Li metal anode that a LiF-rich SEI would contribute to improved low-temperature performance for Li metal batteries^35^.

### Testing of full Na metal coin cells at temperatures ≤0 °C

**Fig. 6:**
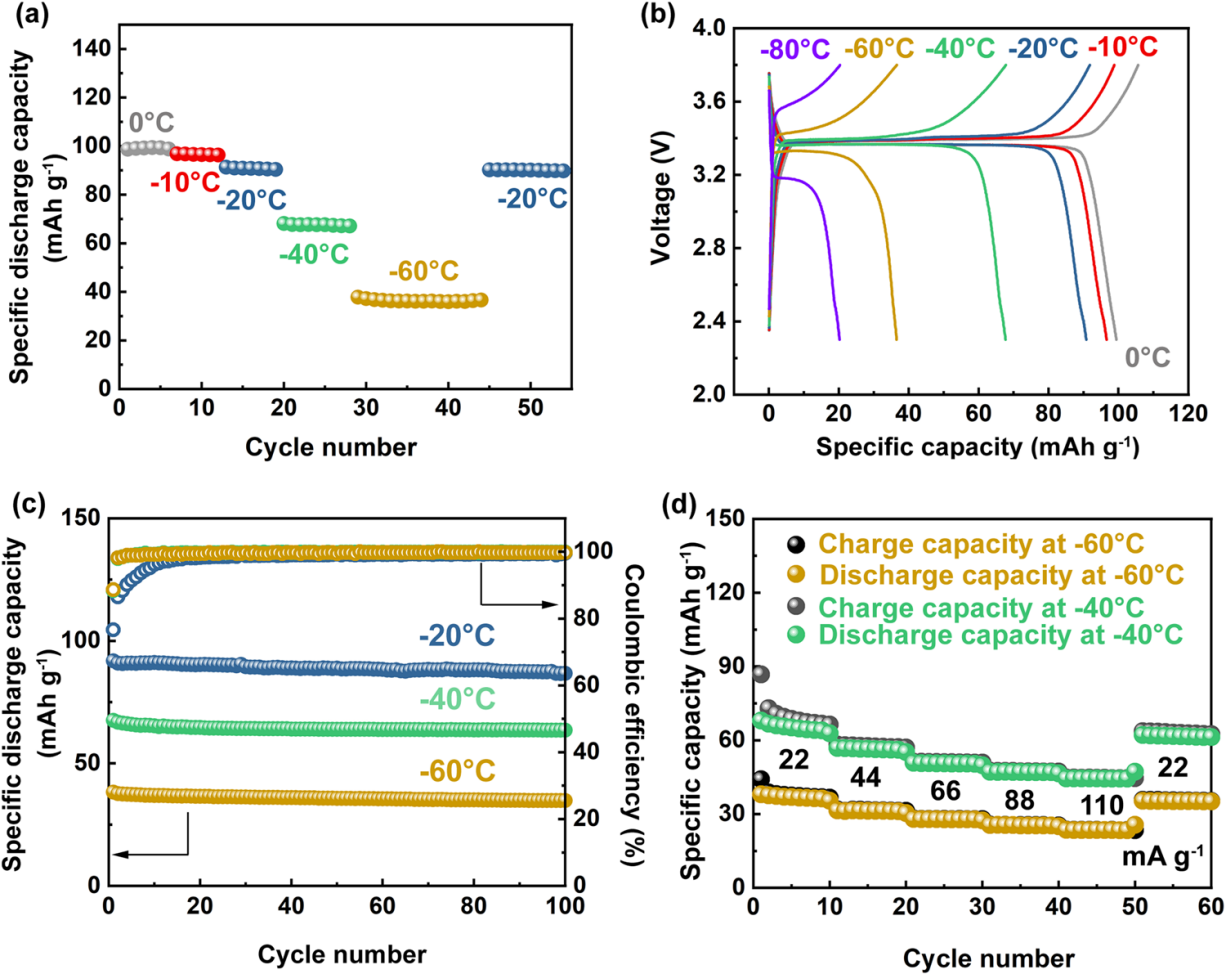
Electrochemical energy storage performance of Na||Na_3_V_2_(PO_4_)_3_ coin cells using the 0.5 M NaOTf-DEGDME/DOL (2:8) electrolyte solution at low temperatures. **a** Temperature-dependent galvanostatic cycling of cells at 22 mA g^−1^ (equivalent to 0.2C, 1C=110 mA g^−1^, based on the active material Na_3_V_2_(PO_4_)_3_) down to −60 °C with voltage cutoffs of 2.3 and 3.8 V. Note that the duration of cycling at each temperature was kept the same for comparison. **b** Galvanostatic charge–discharge voltage profiles at 22 mA g^−1^ from 0 °C to −80 °C. **c** Long-term galvanostatic cycling of cells at 22 mA g^−1^ at −20 °C, −40 °C and −60 °C. **d** Rate cycling performance (up to 110 mA g^−1^, equivalent to 1C) of cells at −40 °C and −60 °C.

Sodium metal batteries comprising Na_3_V_2_(PO_4_)_3_ as the cathode^8^ (active material loading: ~2 mg cm^−2^) and Na metal as the anode in the 0.5 M NaOTf-DEGDME/DOL (2:8) electrolyte were tested at 22 mA g^−1^ (equivalent to 0.2C, 1C = 110 mA g^−1^) and low temperatures down to −80 °C (Fig. 6 and Supplementary Fig. 43). Galvanostatic cycling (Fig. 6a) shows that the discharge capacity decreases as the cell is sequentially cooled. At −60 °C, ~42% of the specific capacity obtained at −20 °C can be retained. The capacity loss from the temperature stepping is reversible, and the capacity is fully recovered as the temperature rises back to −20 °C (Fig. 6a). In addition, the CE increases with decreasing temperature. It increases from an average value of 91.3% at 0 °C to 99.9% at −60 °C, and then decreases to 99.5% when the temperature increases back to −20 °C (Supplementary Fig. 43a). The charge–discharge voltage profiles from 0 °C to −80 °C (Fig. 6b) suggest that the cell voltage hysteresis is also temperature sensitive. At −60 °C, the cell voltage hysteresis is approximately four times that at −40 °C and five times that at −20 °C. For long-term cycling (Fig. 6c), a cell at −20 °C exhibits an initial discharge capacity of 92 mAh g^-1^ and a capacity retention of 94% after 100 cycles, with an average CE of 98.1%. At −40 °C, the initial capacity decreases to 68 mAh g^-1^ with a capacity retention of 94% after 100 cycles, while a higher average CE (99.6%) can be achieved. Even at −60 °C, a similar CE (99.5%) can still be maintained, with a capacity retention of 91% (decay rate < 0.089% per cycle) after 100 cycles. The enlarged CE data are shown in Supplementary Fig. 43b. Figure 6d shows the rate performance of the cells at currents up to 110 mA g^−1^ (1 C) at −40 °C and −60 °C, which further confirms the ability of Na metal to be cycled in the full cell configuration using the designed electrolyte under extremely cold conditions. The corresponding CE data at different rates are detailed in Supplementary Fig. 43c, which shows that the CE at both temperatures increases with increasing current rate.

Good cycling performance is also achieved at higher mass loadings of Na_3_V_2_(PO_4_)_3_ up to 3.0 mg cm^−2^ at −40 °C (Supplementary Figs. 44 and 45). Moreover, an anode of Na@MXene (Na metal incorporated into an MXene electrode)^36^ is employed for pairing with Na_3_V_2_(PO_4_)_3_ at −40 °C, showing a stable performance (Supplementary Figs. 46 and 47), which demonstrates a proof-of-concept full cell with less Na metal (2 mAh g^−1^) employed on the anode side.

In summary, we have formulated low-temperature electrolytes comprising acyclic/cyclic ethers (DEGDME/DOL) and NaOTf salt, which extend the operating temperature limit for Na metal batteries. In particular, a single-solvent electrolyte of 1 M NaOTf-DEGDME enables stable Na metal cycling down to −40 °C, showing a low overpotential of 100 mV at 1 mA cm^−2^ with a capacity of 1 mAh cm^−2^ for over 300 h. Theoretical calculations show that NaOTf has a moderately high reduction potential (1.02 V vs. Na/Na^+^) compared to the other commonly used Na salts, leading to the formation of an inorganic protective layer before the electrolyte solvent is decomposed, but with a moderate driving force. This could contribute to a moderate reaction with Na metal and thus the formation of a smooth and uniform SEI. To further decrease the temperature threshold, DOL was introduced to form binary-solvent electrolytes. An optimized electrolyte consisting of 0.5 M NaOTf-DEGDME/DOL (2:8 in volume ratio) exhibits a resistance increase of only 3.4 times from +20 °C to −80 °C and high thermal stability with no phase transition down to −150 °C. We further demonstrate stable Na cycling with a small overpotential of 50 mV for over 2000 h at −80 °C. The good electrochemical performance could be ascribed to the formation of a robust NaF-rich SEI (with high NaF content in the upper layer) with uniform morphology and mechanical strength, revealed by a suite of characterizations, including SEM, AFM, XPS and cryo-TEM. As a proof of concept, sodium metal batteries comprising Na_3_V_2_(PO_4_)_3_ as the cathode and Na metal as the anode present low capacity decay rates and high CE values during cycling down to −60 °C. In addition, sodium metal full cells with a controlled amount of Na metal employed as the anode have also been demonstrated.

## Methods

### Materials

Metallic sodium (Na) chips (average thickness of 1 mm) were made from rolling-pressed Na metal cubes (99.9% trace metals basis, Sigma–Aldrich) in an argon (Ar)-filled glove box (O_2_ < 0.6 PPM, H_2_O < 0.1 PPM, Mbraun), whose oxidized surfaces were cut off using a knife. Sodium hexafluorophosphate (NaPF_6_, Sigma–Aldrich), sodium trifluoromethanesulfonate (NaOTf, Beantown Chemical), sodium perchlorate (NaClO_4_, Sigma–Aldrich), sodium bis(trifluoromethanesulfonyl)imide (NaTFSI, Solvionic) and sodium bis(fluorosulfonyl)imide (NaFSI, Solvionic) were dried in glass vials for over 12 hours in the glove box (O_2_ < 0.6 PPM, H_2_O < 0.1 PPM, Mbraun) before being used. Diethylene glycol dimethyl ether (DEGDME, anhydrous, H_2_O < 50 ppm, Sigma–Aldrich), 1,2-dimethoxyethane (anhydrous, H_2_O < 30 ppm water, Sigma–Aldrich) and 1,3-dioxolane (anhydrous, H_2_O < 30 ppm, Sigma–Aldrich) were directly used as solvents for preparing electrolytes.

To synthesize Na_3_V_2_(PO_4_)_3_, 0.364 g vanadium oxide (V_2_O_5_, Alfar Aesar) and 0.54 g oxalic acid (H_2_C_2_O_4_, anhydrous, Alfar Aesar) were dissolved in 20 mL distilled water at 70 °C. Sodium dihydrogen phosphate (NaH_2_PO_4_, 0.72 g, Alfar Aesar), 0.2 g glucose (anhydrous, Alfar Aesar) and 50 mL 1-propanol (Alfar Aesar) were added and mixed, followed by drying at 70 °C. The dried sample was ground and annealed at 400 °C for 4 h and then 750 °C for 8 h under an Ar gas atmosphere with a heating rate of 5 °C min^−1^. The annealed powder was mixed with carbon black (Super C65, C-NERGY) and polyvinylidene fluoride (PVDF, HSV 900, Arkema) in a weight ratio of 6:3:1 using 1-methyl-2-pyrrolidone (NMP, anhydrous, Sigma–Aldrich) to form a slurry. The slurry was cast onto aluminum foil (99.9% purity, 16 µm thickness, MTI) and dried at 70 °C in a vacuum oven, followed by cutting the specimen into a cathode with a diameter of 12 mm (average thickness of 60–160 µm). The active material loading was up to 3 mg cm^−2^.

To synthesize Ti_3_C_2_ MXene, 3 g Ti_3_AlC_2_ powder (98%, 400 mesh, Jilin 11 Technology Co., Ltd, China) was added into 30 mL hydrofluoric acid (HF) solution (40%, Alfa Aesar) under stirring for 24 h to obtain Ti_3_C_2_ MXene. The obtained Ti_3_C_2_ MXene was washed with deionized (DI) water until a pH value of 6–7 was reached for the solution, which was then freeze-dried. Then, 0.25 g as-prepared Ti_3_C_2_ MXene was weighed and added into a 50 mL cetyltrimethylammonium bromide (CTAB) solution (0.20 wt %, 99%, Alfa Aesar) under stirring at 40 °C for 24 h. The collected Ti_3_C_2_ MXene was washed using DI water and dried at 70 °C. After that, 0.25 g as-obtained Ti_3_C_2_ MXene was weighed and dispersed in 40 mL NaOH solution (1 M) under stirring (500 rpm) at room temperature for 72 h, and then, the precipitate was collected and washed using DI water and freeze-dried. The electrode slurry composed of the as-synthesized Ti_3_C_2_ MXene and PVDF in a weight ratio of 9:1 (dispersed in NMP) was cast on Cu foil (99.8% purity, 9 µm thickness, MTI). The wet coating was dried at 70 °C in a vacuum oven, followed by cutting into an anode with a diameter of 12 mm (average thickness of 100 µm). The active material loading was up to 2 mg cm^−2^.

### Physicochemical characterizations

Dynamic viscosity (or absolute viscosity) measurements were conducted using a viscometer (*micro*VISC^TM^, RheoSense), which was specifically calibrated for low-temperature measurement (down to −40 °C), in the glove box (O_2_ < 0.6 PPM, H_2_O < 0.1 PPM, Mbraun). Before testing, each sample (100 µL) was isothermally stored at the target temperature for over 10 h. Scanning electron microscopy (SEM) images were obtained using a field emission gun environmental SEM system (XL 300 ESEM-FEG). Cryogenic transmission electron microscopy (cryo-TEM) analysis was conducted using an FEI Tecnai F30 TEM operating at 300 kV. All images were recorded at a low dose rate between 1 and 2 × 10^2^ electrons·Å^−2^·s^−1^. X-ray photoelectron spectroscopy (XPS) analysis was performed via a PHI Versaprobe II scanning XPS microprobe with a 0.47 eV system resolution using a monochromatic 1486.7 X-ray source. Differential scanning calorimetry (DSC) was performed using a Netzsch DSC 204 F1 Phoenix. Both the samples and reference were tested between −150 °C and +20 °C at a controlled rate of 10 °C min^−1^. Atomic force microscopy (AFM) was conducted on copper foils after Na plating/stripping using a Bruker’s Dimension Icon AFM with an RTESPA-150 probe.

### Electrochemical characterizations

A temperature chamber (MC-812, ESPEC) was employed to ensure stable temperature environments between −80 °C and +20 °C. Tested coin cells were maintained at a specified temperature for at least an hour to attain thermal equilibrium. The electrolytes were prepared in the glove box (O_2_ < 0.6 PPM, H_2_O < 0.1 PPM, Mbraun). The masses of salts were weighed using a balance (PA84C, OHAUS), and the volumes of solvents were measured using pipettes (100–1000 µL, High Performance Single-Channel Pipettor, VWR). Galvanostatic Na||Na symmetric cycling was conducted in 2032-type coin cells, which were assembled with two identical Na chips (average thickness of 1 mm, diameter of 12 mm) and a separator (Celgard 2400, thickness of 24 µm, diameter of 15 mm, average porosity of 39%) filled with 40 µL electrolyte. Asymmetric coin cells were also assembled using stainless steel foil (thickness of 0.5 mm, 316 stainless steel) as the working electrode and freshly cut Na as the counter electrode with a Celgard 2400 separator and 40 µL electrolyte. CE measurements were performed in asymmetric coin cells by plating and stripping at a current density of 0.5 mA cm^−2^ for over 1 h (0.5 mAh cm^−2^ deposited or stripped per half cycle). Sodium metal cells were assembled with Na_3_V_2_(PO_4_)_3_ cathodes and Na metal or Na@MXene anodes with the tested electrolytes. To prepare Na@MXene electrodes, 2 mAh cm^−2^ Na was deposited onto the Ti_3_C_2_ MXene electrode using asymmetric Na||MXene cells at 0.5 mA cm^−2^ in the tested electrolyte with a Celgard 2400 separator. After Na deposition, the Na||MXene cells were disassembled, and the Na@MXene electrodes were collected. Both symmetric cells and sodium metal batteries were tested using a standard battery tester (CT2001A, Wuhan LANHE Electronics Co., Ltd). Electrochemical impedance spectroscopy (EIS) was conducted using an electrochemical workstation (VMP3, Bio-Logic Science Instruments) at a scanning frequency from 1 MHz to 0.1 Hz with an AC amplitude of 5 mV and 10 points per decade. The average open-circuit voltage time before EIS measurement was ~2 h.

The total resistance of the electrolyte solution (*R*_*e*_) was measured through EIS in stainless steel||stainless steel cells^19^. A polymer membrane (Celgard, average thickness of 25 µm, porosity of 39%, diameter of 15 mm) was sandwiched between two pieces of 316 stainless steel (thickness: 0.5 mm) in a 2032-type coin cell with 40 µL electrolyte added (Supplementary Fig. 3). The ionic conductivity (*δ*) of the electrolyte solution was calculated based on the equation below:

$$\delta=L/(A{{\cdot }}Re)$$

where *R*_*e*_ is the resistance value from EIS fitting curve using the equivalent electrical circuit^37,38^, *L* is the distance between the two pieces of stainless steel, and *A* is the product of the membrane area and porosity.

TEM samples were prepared by carrying out a single Na plating/stripping cycle (0.25 mA cm^−2^ for 2 h) using a Cu TEM grid as the working electrode. The coin cell containing the grid was disassembled in an Ar-filled glove box, and the grid was washed with 0.5 mL DEGDME and vacuum dried inside the glove box. Next, the grid was loaded into a Gatan cryo-specimen holder inside the glove box. The Cu TEM grid was quickly transferred (~10 s air exposure) from the argon environment into the TEM load lock, where it was brought to vacuum. Next, the temperature of the sample was decreased using liquid nitrogen and maintained at −175 °C during imaging. The XPS samples were transferred into the chamber via a sealed Ar-filled vessel to avoid exposure to air. Regarding the changes in the electrolyte resistance (Fig. 4b), the averages and deviations were based on three consecutive EIS measurements.

### First-principles computations

Density functional simulations were carried out using the Gaussian 16 package^39^. We used the B3LYP functional with the D3 version of Grimme (empirical dispersion = gd3) and a 6-311++G basis set^40–43^. Solvent effects were included with the polarization continuum model (PCM) using DEGDME as the solvent (dielectric constant = 7.39)^44^. The reduction potential was computed using the thermodynamic cycle described by Han et al. and Borodin et al.^45,46^. The reduction potential, ${E}_{{red}}^{0}$, was calculated based on the equation below:

$${E}_{{red}}^{0}=-\frac{\left[\triangle {G}_{{ea}}+\triangle {G}_{{sol}}\left({S}^{-}\right)-\triangle {G}_{{sol}}\left(S\right)\,\right]}{F}-1.8$$

where $\triangle {G}_{{ea}}$ is the free energy of electron affinity computed in the gas phase, $\triangle {G}_{{sol}}\left({S}^{-}\right)$ and $\triangle {G}_{{sol}}\left(S\right)$ are the Gibbs free energy for the solvation process of charged and neutral species, respectively, and *F* is the Faraday constant. To convert the potential to the Na/Na^+^ reference according to previous works^45,46^, 1.8 V was extracted from the equation. The International Union of Pure and Applied Chemistry recommends a value of 4.42–4.44 V on the absolute potential scale^47^.

### Reporting summary

Further information on research design is available in the Nature Research Reporting Summary linked to this article.

## Supplementary information

Supplementary Information

Reporting Summary

## Data Availability

The data that support the findings of this study are available within the text, including the Methods and Supplemental information. Raw datasets related to the current work are available from the corresponding author on reasonable request.
