# Supplementary material for: Extending the low-temperature operation of sodium metal batteries combining linear and cyclic ether-based electrolyte solutions
Source: Nat Commun. 2022 Aug 22;13:4934. doi: 10.1038/s41467-022-32606-4 (PMC9395411; doi:10.1038/s41467-022-32606-4)
Supplement: Supplementary file 1 — Supplementary Information [file 41467_2022_32606_MOESM1_ESM.pdf]

Supplementary information:

Extending the low-temperature operation of sodium metal batteries combining linear and cyclic ether-based electrolyte solutions

Chuanlong Wang[1], Akila C. Thenuwara[2], Jianmin Luo[1], Pralav P. Shetty[2], Matthew T. McDowell[2,3], Haoyu Zhu[4], Sergio Posada-Pérez[5,6], Hui (Claire) Xiong[4,7,*], Geoffroy Hautier[1,5,*], Weiyang Li[1,*]

[*]Corresponding author

Email: clairexiong@boisestate.edu

Email: geoffroy.hautier@dartmouth.edu

Email: weiyang.li@dartmouth.edu

[1]Thayer School of Engineering, Dartmouth College, 14 Engineering Drive, Hanover, NH, 03755, USA

[2]G.W. Woodruff School of Mechanical Engineering, Georgia Institute of Technology, 801 Ferst Drive, Atlanta, GA, 30332, USA

[3]School of Materials Science and Engineering, Georgia Institute of Technology, 771 Ferst Drive, Atlanta, GA, 30332, USA

[4]Micron School of Materials Science and Engineering, Boise State University, 1910 University Drive, Boise, ID, 83725, USA

[5]Institute of Condensed Matter and Nanosciences, UCLouvain, Chemin des Étoiles 8, B-1348 Louvain-la-Neuve, Belgium

[6]Institut de Química Computacional i Catàlisi and Departament de Química, Universitat de Girona, C/ Maria Aurèlia Capmany, 69, 17003 Girona, Catalonia, Spain

[7]Center for Advanced Energy Studies, Idaho Falls, ID, 83401, USA

Table of contents

Supplementary Figure 1 | Radar chart comparing performance of Na and Li metal anodes cycling at low temperatures (≤−40ºC)... 6

Supplementary Figure 2 | Investigation of the electrolyte consisting of 1 M $NaPF_6$-DEGDME across a range of temperatures. 7

Supplementary Figure 3 | Schematic illustration and photos of a stainless steel||stainless steel symmetric cell configuration used to measure the electrolyte conductivity.. 8

Supplementary Figure 4 | Galvanostatic cycling of Na||Na symmetric cells operating at +20˚C in eight different electrolyte solutions... 9

Supplementary Figure 5 | Enlarged voltage profiles at the 50th cycle (100 hours) of Na||Na symmetric cells cycling at +20˚C in six electrolyte solutions... 10

Supplementary Figure 6 | SEM images of Na metal electrode surfaces after 50 cycles at +20˚C (symmetric Na||Na cells) in 1 M NaOTf-DEGDME, NaFSI-DME, $NaClO_4$-DEGDME, NaTFSI-DOL electrolytes 11

Supplementary Figure 7 | SEM images of Na metal electrode surfaces after cycling at +20˚C (symmetric Na||Na cells) in 1 M NaFSI-DEGDME, $NaClO_4$-DME, NaTFSI-DME, NaTFSI-DEGDME electrolytes 12

Supplementary Figure 8 | SEM images of Na metal electrode surfaces after 50 cycles at −20˚C (symmetric Na||Na cells) in 1 M NaOTf-DEGDME, $NaClO_4$-DME, NaTFSI-DME, NaFSI-DME electrolytes.. 13

Supplementary Figure 9 | SEM images of Na metal electrode surfaces after 50 cycles at −20˚C (symmetric Na||Na cells) in 1 M $NaClO_4$-DEGDME, NaTFSI-DEGDME, NaFSI-DEGDME, NaTFSI-DOL electrolytes 14

Supplementary Figure 10 | XPS profiles of the Na metal electrode surfaces in 1 M NaOTf-DEGDME at +20˚C, −20˚C and −40˚C 15

Supplementary Figure 11 | XPS depth profile analysis on the Na metal electrode after 50 cycles (symmetric Na||Na cells) in 1 M NaOTf-DEGDME electrolyte at +20˚C..................... 16

Supplementary Figure 12 | XPS depth profile analysis on the Na metal electrode after 50 cycles (symmetric Na||Na cells) in 1 M NaOTf-DEGDME electrolyte at −20˚C..................... 17

Supplementary Figure 13 | XPS depth profile analysis on the Na metal electrode after 50 cycles (symmetric Na||Na cells) in 1 M NaOTf-DEGDME electrolyte at −40˚C..................... 18

Supplementary Figure 14 | XPS of the Na metal electrode surface after 50 cycles (symmetric Na||Na cells) in 1 M $NaClO_4$-DME electrolyte at +20˚C and −20˚C....................................... 19

Supplementary Figure 15 | XPS depth profile analysis on the Na metal electrode after 50 cycles (symmetric Na||Na cells) in 1 M $NaClO_4$-DME electrolyte at +20˚C 20

Supplementary Figure 16 | XPS depth profile analysis on the Na metal electrode after 50 cycles (symmetric Na||Na cells) in 1 M $NaClO_4$-DME electrolyte at −20˚C 21

Supplementary Figure 17 | XPS of the Na metal electrode surface after 50 cycles (symmetric Na||Na cells) in 1 M NaFSI-DME electrolyte at +20˚C and −20˚C 22

Supplementary Figure 18 | XPS depth profile analysis on the Na metal electrode after 50 cycles (symmetric Na||Na cells) in 1 M NaFSI-DME electrolyte at +20˚C 23

Supplementary Figure 19 | XPS depth profile analysis on the Na metal electrode after 50 cycles (symmetric Na||Na cells) in 1 M NaFSI-DME electrolyte at −20˚C 24

Supplementary Figure 20 | XPS of the Na metal electrode surface (symmetric Na||Na cells) in 1 M NaTFSI-DEGDME electrolyte at +20˚C and −20˚C 25

Supplementary Figure 21 | XPS depth profile on the Na metal electrode after 15 cycles (symmetric Na||Na cells) in 1 M NaTFSI-DEGDME electrolyte at +20˚C 26

Supplementary Figure 22 | XPS depth profile on the Na metal electrode after 50 cycles (symmetric Na||Na cells) in 1 M NaTFSI-DEGDME electrolyte at −20˚C 27

Supplementary Figure 23 | Illustration of tailoring low-temperature electrolytes comprising a sodium salt (NaOTf) and binary solvents of an acyclic ether (DEGDME) and a cyclic ether (DOL). 28

Supplementary Figure 24 | Temperature-dependent galvanostatic cycling of Na||Na symmetric cells in 1 M NaOTf-DEGDME/DOL (8:2 and 5:5 in volume ratio).. 29

Supplementary Figure 25 | Temperature-dependent galvanostatic cycling of Na||Na symmetric cells in 0.5 M NaOTf-DEGDME/DOL (8:2 in volume ratio) and 0.5 M NaOTf-DME/DOL (8:2, 5:5 and 2:8 in volume ratio).. 30

Supplementary Figure 26 | Temperature-dependent galvanostatic cycling of Na||Na symmetric cells in 0.5 M $NaClO_4$-DME/DOL (8:2 and 5:5 in volume ratio).. 31

Supplementary Figure 27 | EIS spectra of 0.5 M NaOTf-DEGDME/DOL (2:8), 0.5 M NaOTf-DEGDME/DOL (5:5) and 1 M NaOTf-DEGDME electrolyte solutions using the symmetric stainless steel||stainless steel cells across a range of temperatures 32

Supplementary Figure 28 | Temperature dependent ionic conductivity of $NaClO_4$-DME, NaFSI-DME, NaTFSI-DEGDME, and NaTFSI-DOL electrolyte solution (all in 1 M salt concentration). 33

Supplementary Figure 29 | Temperature-dependent dynamic viscosity of DEGDME and DEGDME/DOL (8:2, 5:5 and 2:8 in volume ratio). 34

Supplementary Figure 30 | Photographic pictures of *(1)* 1 M NaOTf-DEGDME, *(2)* 0.5 M NaOTf-DEGDME/DOL (5:5) and *(3)* 0.5 M NaOTf-DEGDME/DOL (2:8) after storing at +20˚C and −80˚C for 24 hours 35

Supplementary Figure 31 | Galvanostatic cycling of Na||Na symmetric cells in 0.5 M NaOTf-DEGDME/DOL (2:8 and 5:5 in volume ratio) at 0.2 mA cm^{-2} and 0.1 mAh cm^{-2} at −80°C. . 36

Supplementary Figure 32 | AFM characterization of SEI formed on copper foil after Na plating/stripping at 0.5 mA cm^{-2} and 0.5 mAh cm^{-2} with a cut-off voltage of 1.5 V after the 1st cycle (Cu foil is fully desodiated) in 1 M NaOTf-DEGDME electrolyte at −40°C. 37

Supplementary Figure 33 | Na plating/stripping profile of Na||Cu asymmetric cells at 0.5 mA cm^{-2} and 0.5 mAh cm^{-2} with a cut-off voltage of 1.5 V for the 1st cycle at −40˚C................... 38

Supplementary Figure 34 | SEM image of Cu surface after Na plating/stripping (Na||Cu asymmetric cells) for the 1st cycle at 0.5 mA cm^{-2} and 0.5 mAh cm^{-2} with a cut-off voltage of 1.5 V at −40˚C.. 39

Supplementary Figure 35 | SEM of a Na metal electrode surface after 50 cycles (symmetric Na||Na cells) at 0.5 mA cm^{-2} and 0.5 mAh cm^{-2} in 0.5 M NaOTf DEGDME/DOL (5:5) at −80°C 40

Supplementary Figure 36 | SEM of a Na metal electrode surface after 50 cycles (symmetric Na||Na cells) at 0.5 mA cm^{-2} and 0.5 mAh cm^{-2} in 1 M NaOTf DEGDME at −80°C............. 41

Supplementary Figure 37 | XPS of S 2*p*, C 1*s*, O 1*s* and F 1*s* profiles of Na metal electrode surface after 50 cycles (symmetric Na||Na cells) at a current density of 0.5 mA cm^{-2} with a capacity of 0.5 mAh cm^{-2} in 0.5 M NaOTf-DEGDME/DOL (2:8 in volume ratio) electrolyte at −80˚C.. 42

Supplementary Figure 38 | XPS of S 2*p*, C 1*s*, O 1*s* and F 1*s* profiles on Na metal electrode surface after 50 cycles (symmetric Na||Na cells) at a current density of 0.5 mA cm^{-2} with a capacity of 0.5 mAh cm^{-2} in 0.5 M NaOTf-DEGDME/DOL (5:5 in volume ratio) electrolyte at −80˚C 43

Supplementary Figure 39 | XPS depth profile analysis on Na metal electrode after 50 cycles (symmetric Na||Na cells) at a current density of 0.5 mA cm^{-2} with a capacity of 0.5 mAh cm^{-2} in 0.5 M NaOTf-DEGDME/DOL (5:5) electrolyte at −80˚C 44

Supplementary Figure 40 | XPS depth profile analysis on Na metal electrode after 50 cycles (symmetric Na||Na cells) at a current density of 0.5 mA cm^{-2} with a capacity of 0.5 mAh cm^{-2} in 0.5 M NaOTf-DEGDME/DOL (2:8) at −80˚C 45

Supplementary Figure 41 | Contents of elements and inorganic components determined by ex situ postmortem XPS depth profiling of the Na metal electrodes (symmetric Na||Na cells) after 50 cycles at a current density of 0.5 mA cm^{-2} with a capacity of 0.5 mAh cm^{-2} in 0.5 M NaOTf-DEGDME/DOL (5:5) at −80˚C... 46

Supplementary Figure 42 | SEI formed after the 1st cycle (after Na is fully stripped) in Na||Cu TEM grid cells at a current density of 0.25 mA cm^{-2} with a capacity of 0.5 mAh cm^{-2} at −40°C using the single-solvent electrolyte of 1 M NaOTf-DEGDME.................................... 47

Supplementary Figure 43 | Coulombic efficiency of Na||$Na_3V_2(PO_4)_3$ coin cells using the 0.5M NaOTf-DEGDME/DOL (2:8) electrolyte solution at low temperatures.................................. 48

Supplementary Figure 44 | Cycling performance of Na||$Na_3V_2(PO_4)_3$ coin cells at different mass loadings of $Na_3V_2(PO_4)_3$ (up to 3.0 mg cm^{-2}) at 22 mA g^{-1} and −40˚C 49

Supplementary Figure 45 | Coulombic efficiency of Na||$Na_3V_2(PO_4)_3$ coin cells at different mass loadings of $Na_3V_2(PO_4)_3$ (up to 3.0 mg cm^{-2}) at −40˚C 50

Supplementary Figure 46 | Galvanostatic cycling of full cells of $Na_3V_2(PO_4)_3$ cathode || Na@MXene anode at 22 mA g^{-1} and −40˚C.. 51

Supplementary Figure 47 | Coulombic efficiency of full cells of $Na_3V_2(PO_4)_3$ cathode || Na@MXene anode at −40˚C ... 52

Supplementary Table 1 | Summary and comparison of the electrochemical cycling performance at temperatures ≤ −40°C of various symmetric and asymmetric cells comprising Na and Li metal electrodes ... 53

Supplementary Table 2 | Physicochemical properties of the electrolyte solvents investigated 54

Supplementary Table 3 | Summary of the dissolution of five Na salts (salt concentration was kept at 1 M) in three different solvents, respectively, at −35°C ... 55

Supplementary Table 4 | Summary of element values (R_s, R_{SEI}, and $R_{electrode}$) obtained from fitting the impedance data of Na||Na symmetric cells containing 1 M $NaPF_6$-DEGDME solution at 20˚C and -20˚C ... 56

Supplementary Table 5 | Summary of resistance values (R_e) obtained from fitting the impedance data of stainless steel||stainless steel symmetric cells containing 1 M $NaPF_6$-DEGDME solution at a range of temperatures ... 57

Supplementary Table 6 | Summary of electrochemical behavior of single-solvent electrolyte systems (salt concentration was kept at 1 M) at 20°C. ... 58

Supplementary Table 7 | Summary of identified XPS peaks ... 59

Supplementary Table 8 | Summary of SEI composition (surface and bulk) ... 60

Supplementary Table 9 | Summary of calculated reduction potentials of the solvents and salt species with or without the Na cation on the calculation (Units in V vs. Na/Na^+). ... 61

Supplementary Table 10 | Summary of salt dissolution in binary solvents at 1 M/0.5 M concentrations at −35°C ... 62

Supplementary Table 11 | Summary of resistance values (R_e) obtained from fitting the impedance data of stainless steel||stainless steel symmetric cells containing 0.5 M NaOTf-DEGDME/DOL (2:8) solution at a range of temperatures. ... 63

Supplementary Table 12 | Summary of resistance values (Re) obtained from fitting the impedance data of stainless steel||stainless steel symmetric cells containing 0.5 M NaOTf-DEGDME/DOL (5:5) solution at a range of temperatures ... 64

Supplementary Table 13 | Summary of resistance values (Re) obtained from fitting the impedance data of stainless steel||stainless steel symmetric cells containing 1 M NaOTf-DEGDME solution at a range of temperatures ... 65

Supplementary Note 1 ... 66

Supplementary Note 2 ... 66

References ... 68

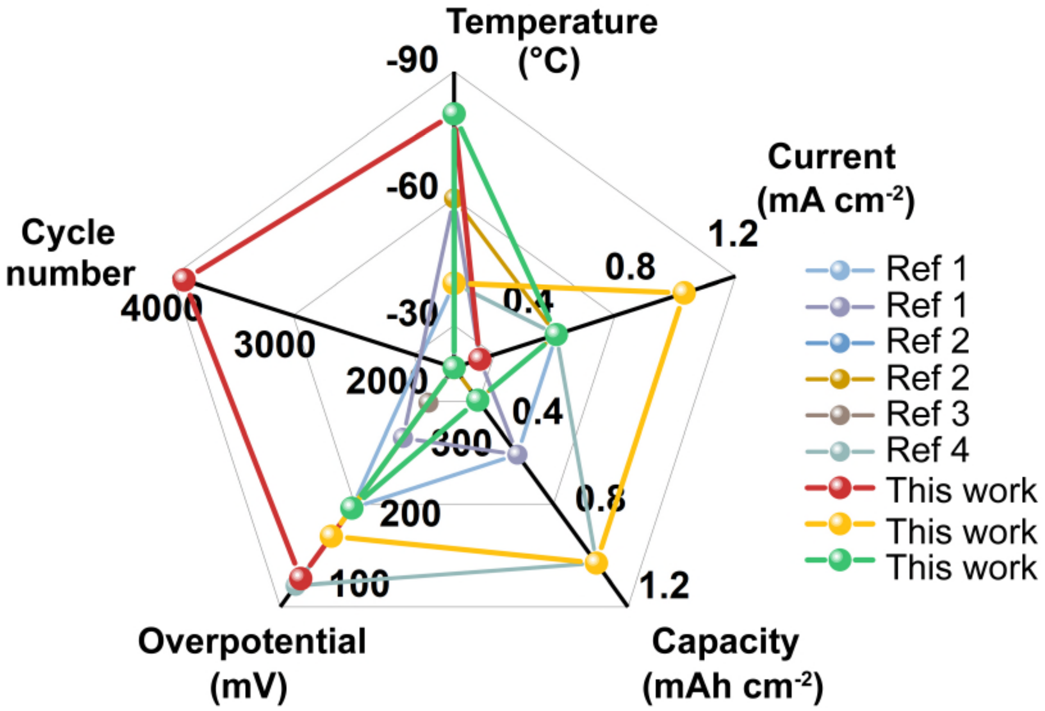

Supplementary Figure 1 | Radar chart comparing performance of Na and Li metal anodes cycling at low temperatures (≤−40ºC)[1-4], based on **Supplementary Table 1**.

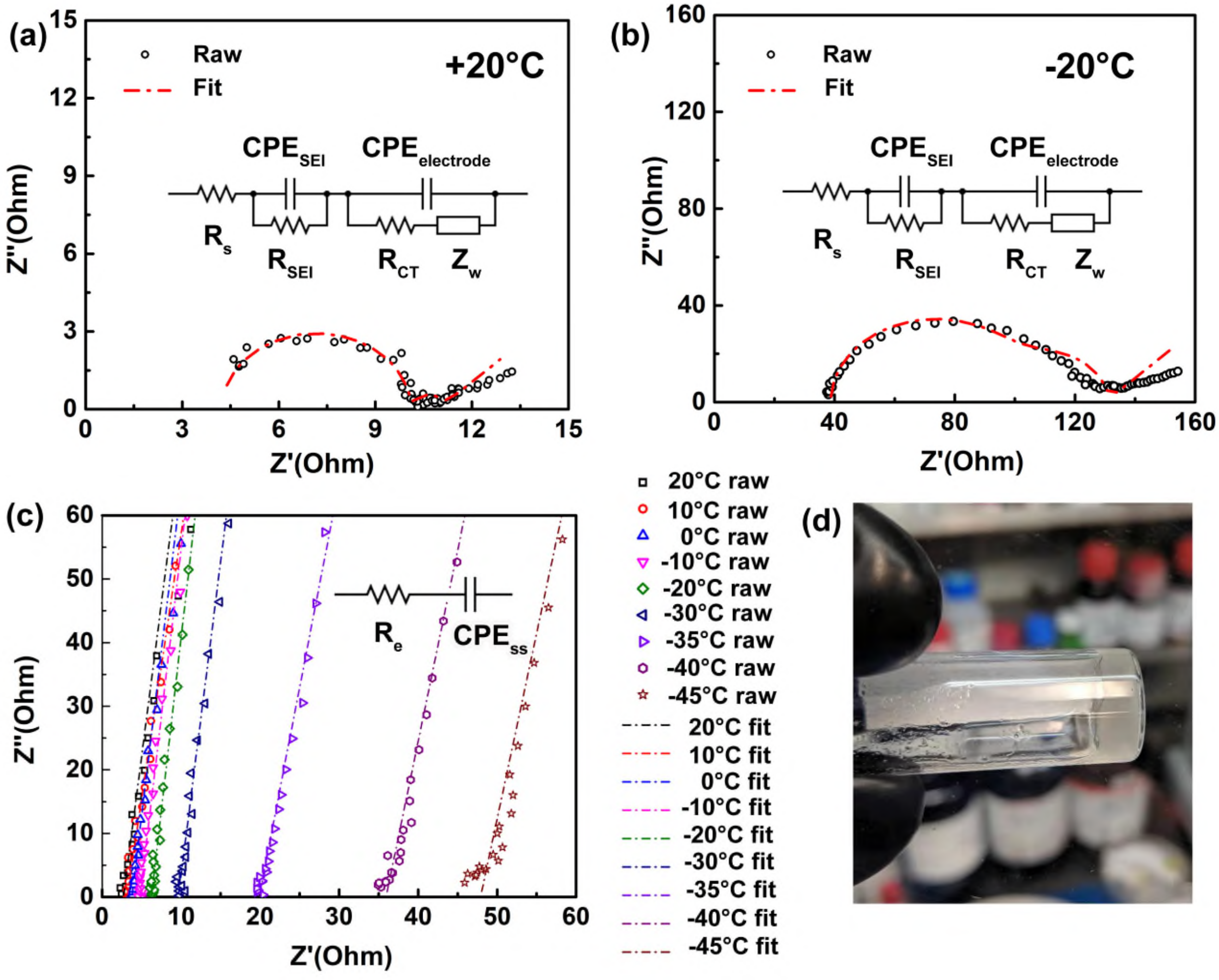

Supplementary Figure 2 | Investigation of the electrolyte consisting of 1 M $NaPF_6$-DEGDME across a range of temperatures. **a**, Electrochemical impedance spectroscopy (EIS) of Na||Na symmetric cells using 1 M $NaPF_6$-DEGDME solution at +20˚C. **b**, EIS of Na||Na symmetric cells using 1 M $NaPF_6$-DEGDME solution at −20˚C. The insets in **a** and **b** present the equivalent circuits used for EIS fitting[2,3,5,6]: R_s, resistance of the electrolyte solution; R_{SEI}, resistance of SEI; CPE_{SEI}, capacitance of SEI; $CPE_{electrode}$, double-layer capacitance; $R_{CT,}$ charge transfer resistance; Z_w, diffusion impedance. **c**, Resistance evaluation of 1 M $NaPF_6$-DEGDME electrolyte solution using the symmetric stainless steel||stainless steel cells at a range of temperatures. The inset presents the equivalent circuit used for EIS fitting:[7,8] R_e, total resistance of the electrolyte; CPE_{ss}, capacitance of blocking electrodes (stainless steel). **d**, Salt precipitation observed at the bottom of a glass vial for 1 M $NaPF_6$-DEGDME electrolyte solution stored at −35˚C for 12 hours.

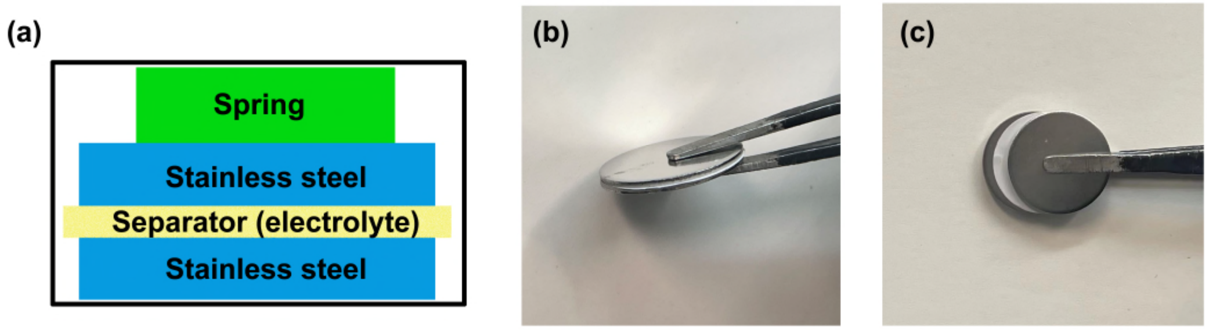

Supplementary Figure 3 | Schematic illustration and photos of a stainless steel||stainless steel symmetric cell configuration used to measure the electrolyte conductivity. **a**, schematic illustration. **b** and **c**, side-view and top-view photos, respectively (the cell is hold by a tweezer for the purpose of photo taken).

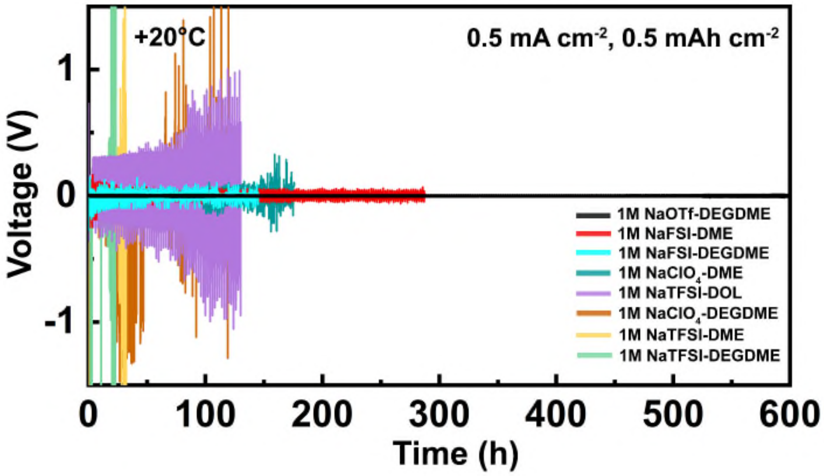

Supplementary Figure 4 | Galvanostatic cycling of Na||Na symmetric cells operating at +20˚C in eight different electrolyte solutions at a current density of 0.5 mA cm^{-2} and a capacity of 0.5 mAh cm^{-2}. Note that performance (overpotential and cycle life) of the Na metal electrode in these eight electrolytes exhibits the following decreasing trend: NaOTf-DEGDME > NaFSI-DME > NaFSI-DEGDME > $NaClO_4$-DME > $NaClO_4$-DEGDME > NaTFSI-DOL > NaTFSI-DEGDME > NaTFSI-DME.

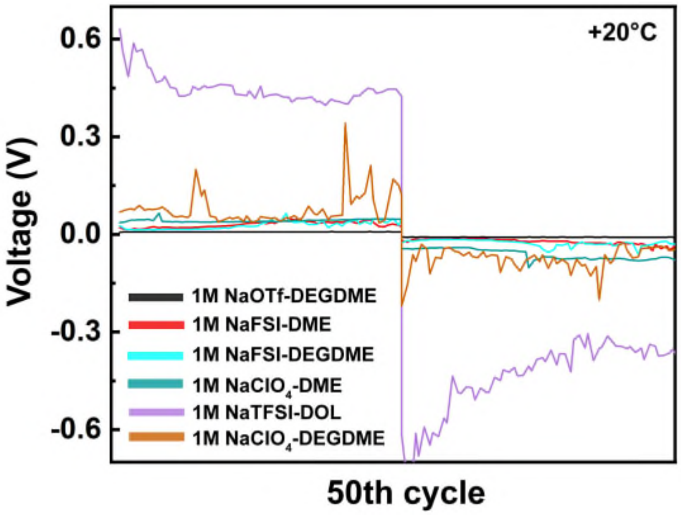

Supplementary Figure 5 | Enlarged voltage profiles at the 50th cycle (100 hours) of Na||Na symmetric cells cycling at +20˚C in six electrolyte solutions, at a current density of 0.5 mA cm^{-2} with a capacity of 0.5 mAh cm^{-2}. Note that the 1 M NaTFSI-DME and NaTFSI-DEGDME electrolyte solutions showed early failure before the 50th cycle, so their curves are not displayed here.

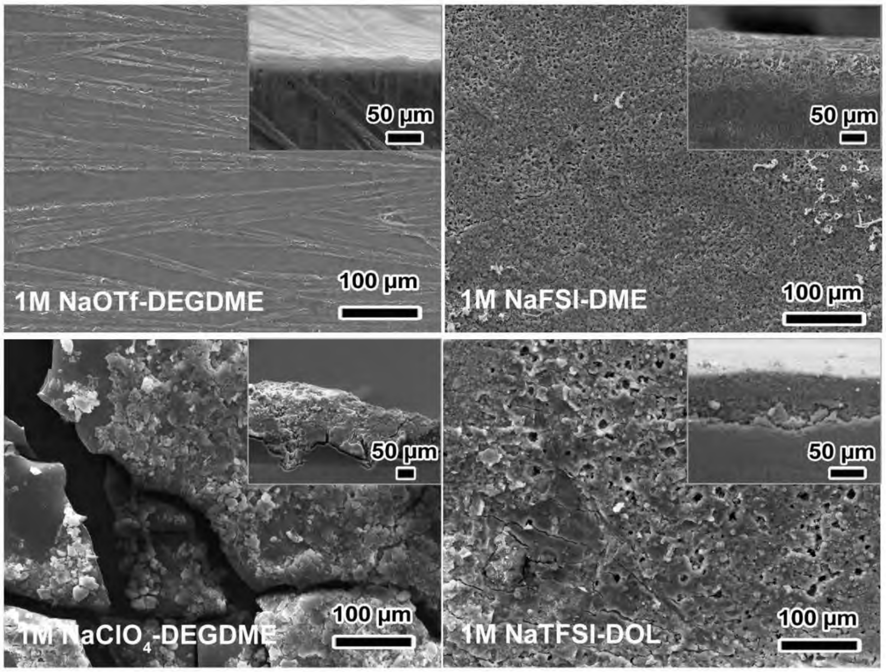

Supplementary Figure 6 | SEM images of Na metal electrode surfaces after 50 cycles at +20˚C (symmetric Na||Na cells) in 1 M NaOTf-DEGDME, NaFSI-DME, $NaClO_4$-DEGDME, NaTFSI-DOL electrolytes at a current density of 0.5 mA cm^{-2} with a capacity of 0.5 mAh cm^{-2} (Insets: corresponding cross-sectional SEM images).

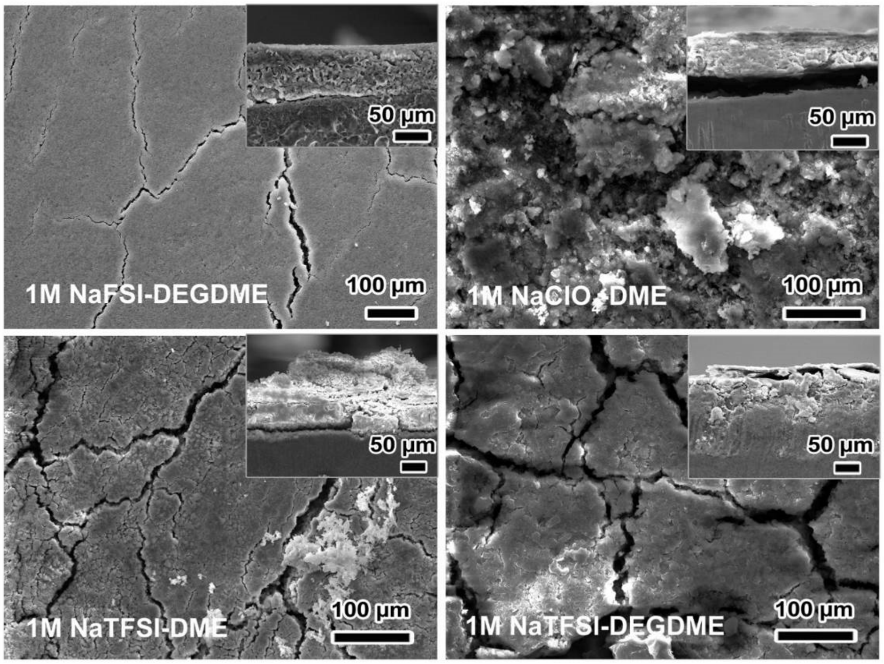

Supplementary Figure 7 | SEM images of Na metal electrode surfaces after cycling at +20˚C (symmetric Na||Na cells) in 1 M NaFSI-DEGDME, $NaClO_4$-DME, NaTFSI-DME, NaTFSI-DEGDME electrolytes at a current density of 0.5 mA cm^{-2} with a capacity of 0.5 mAh cm^{-2}. Note that the cell with 1 M NaTFSI-DME failed at the 22nd cycle and the cell with 1 M NaTFSI-DEGDME failed at the 16th cycle. The cells with 1 M NaFSI-DEGDME and $NaClO_4$-DME were stopped after 50 cycles. Insets: corresponding cross-sectional SEM images.

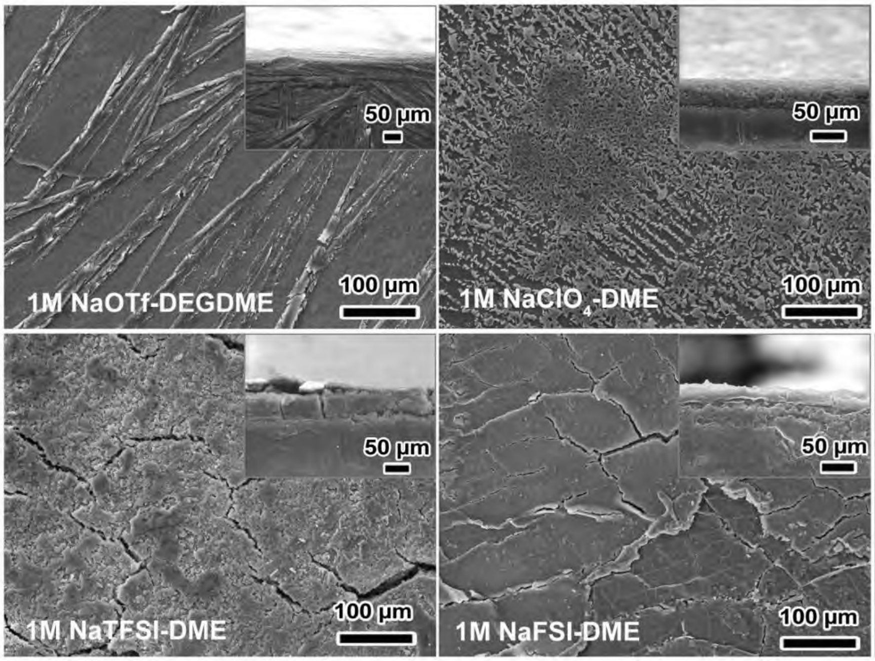

Supplementary Figure 8 | SEM images of Na metal electrode surfaces after 50 cycles at −20˚C (symmetric Na||Na cells) in 1 M NaOTf-DEGDME, $NaClO_4$-DME, NaTFSI-DME, NaFSI-DME electrolytes at a current density of 0.5 mA cm^{-2} with a capacity of 0.5 mAh cm^{-2} (Insets: corresponding cross-sectional SEM images).

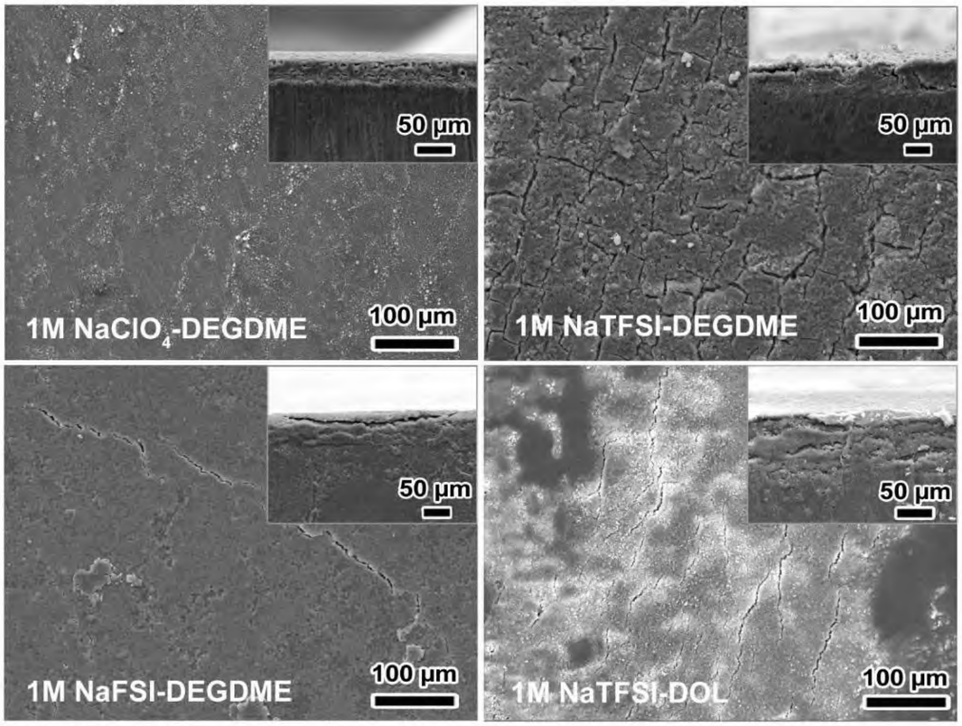

Supplementary Figure 9 | SEM images of Na metal electrode surfaces after 50 cycles at −20˚C (symmetric Na||Na cells) in 1 M $NaClO_4$-DEGDME, NaTFSI-DEGDME, NaFSI-DEGDME, NaTFSI-DOL electrolytes at a current density of 0.5 mA cm^{-2} with a capacity of 0.5 mAh cm^{-2} (Insets: corresponding cross-sectional SEM images).

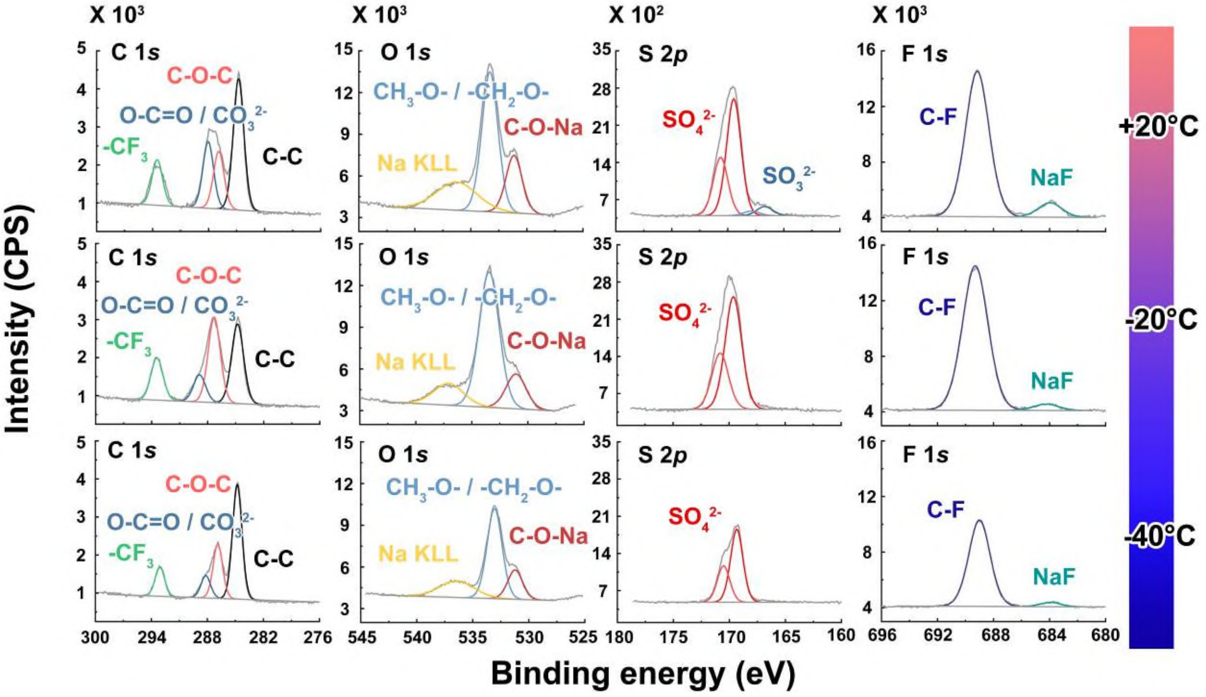

Supplementary Figure 10 | XPS profiles of the Na metal electrode surfaces in 1 M NaOTf-DEGDME at +20˚C, −20˚C and −40˚C using symmetric Na||Na cells after 50 cycles at a current density of 0.5 mA cm^{-2} with a capacity of 0.5 mAh cm^{-2}.

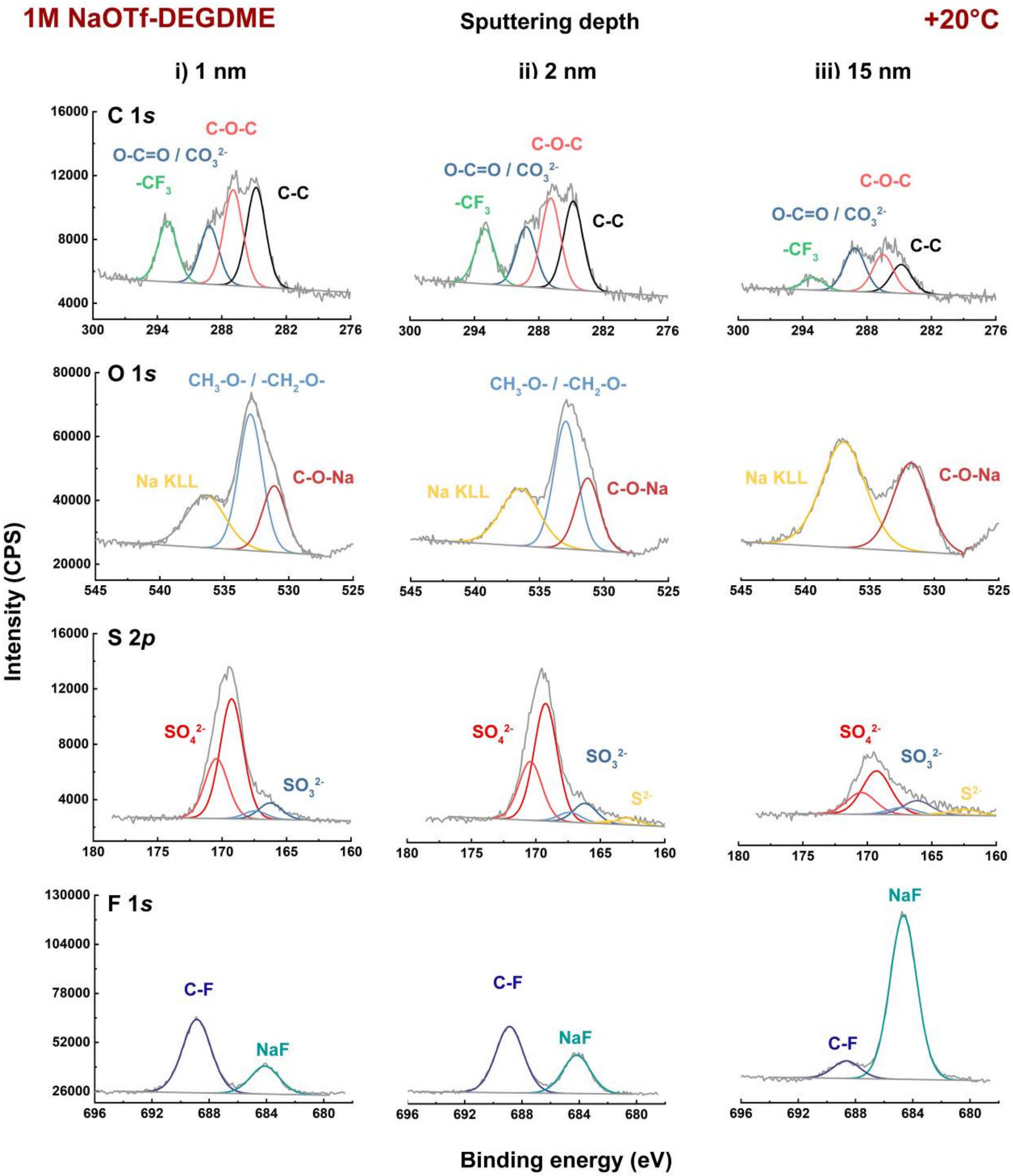

Supplementary Figure 11 | XPS depth profile analysis on the Na metal electrode after 50 cycles (symmetric Na||Na cells) in 1 M NaOTf-DEGDME electrolyte at +20˚C at a current density of 0.5 mA cm^{-2} with a capacity of 0.5 mAh cm^{-2}.

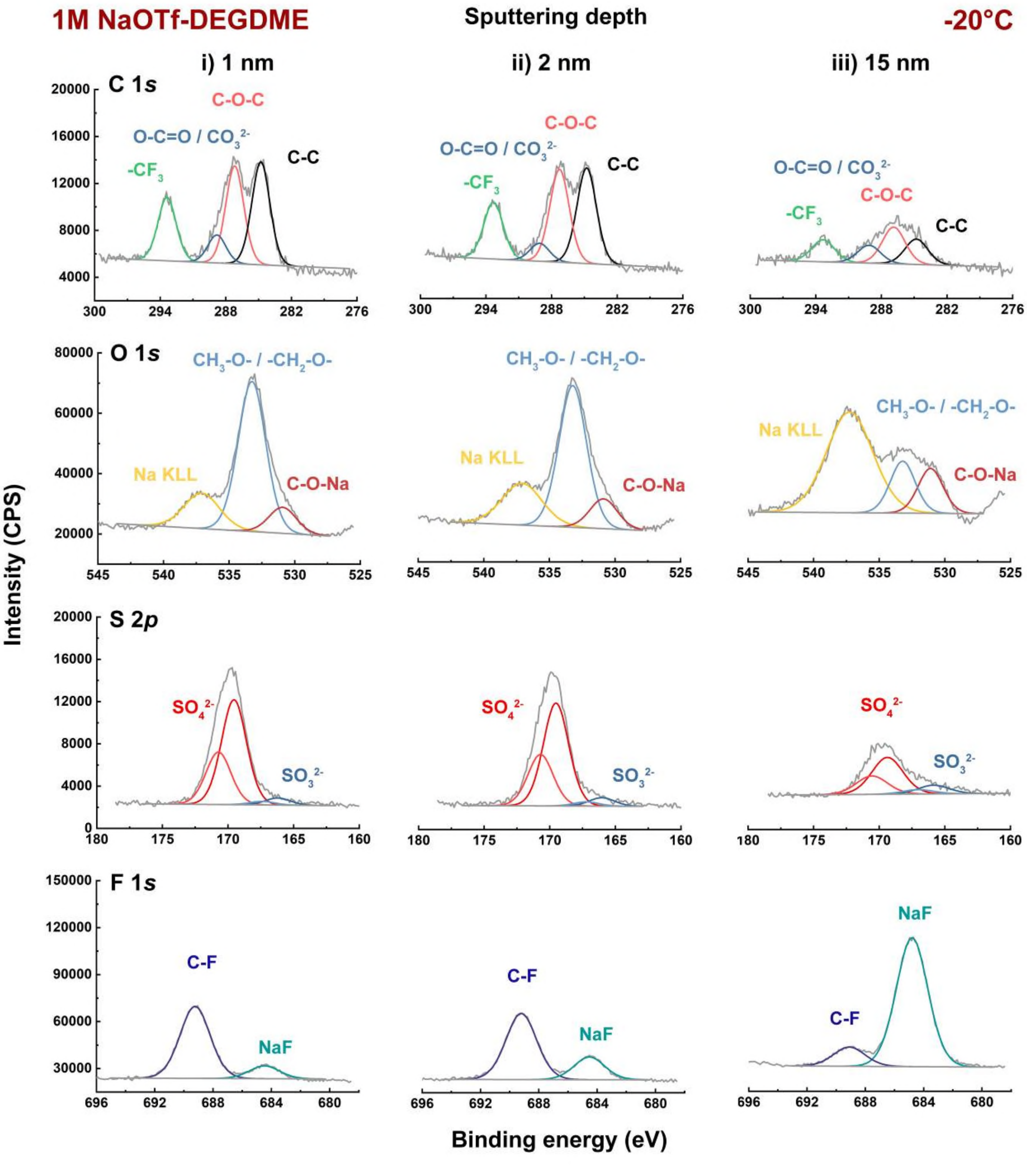

Supplementary Figure 12 | XPS depth profile analysis on the Na metal electrode after 50 cycles (symmetric Na||Na cells) in 1 M NaOTf-DEGDME electrolyte at −20˚C at a current density of 0.5 mA cm^{-2} with a capacity of 0.5 mAh cm^{-2}.

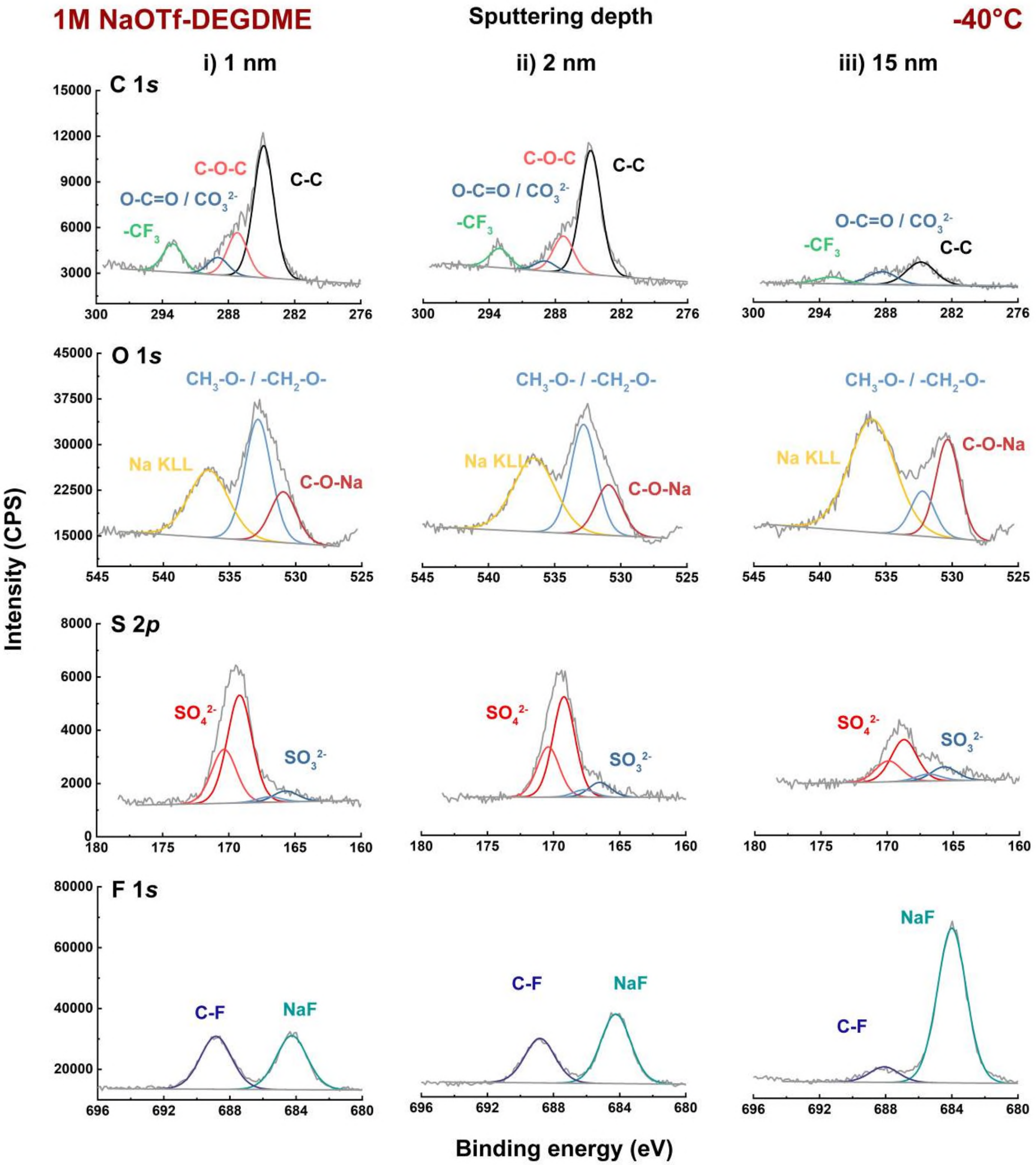

Supplementary Figure 13 | XPS depth profile analysis on the Na metal electrode after 50 cycles (symmetric Na||Na cells) in 1 M NaOTf-DEGDME electrolyte at −40˚C at a current density of 0.5 mA cm^{-2} with a capacity of 0.5 mAh cm^{-2}.

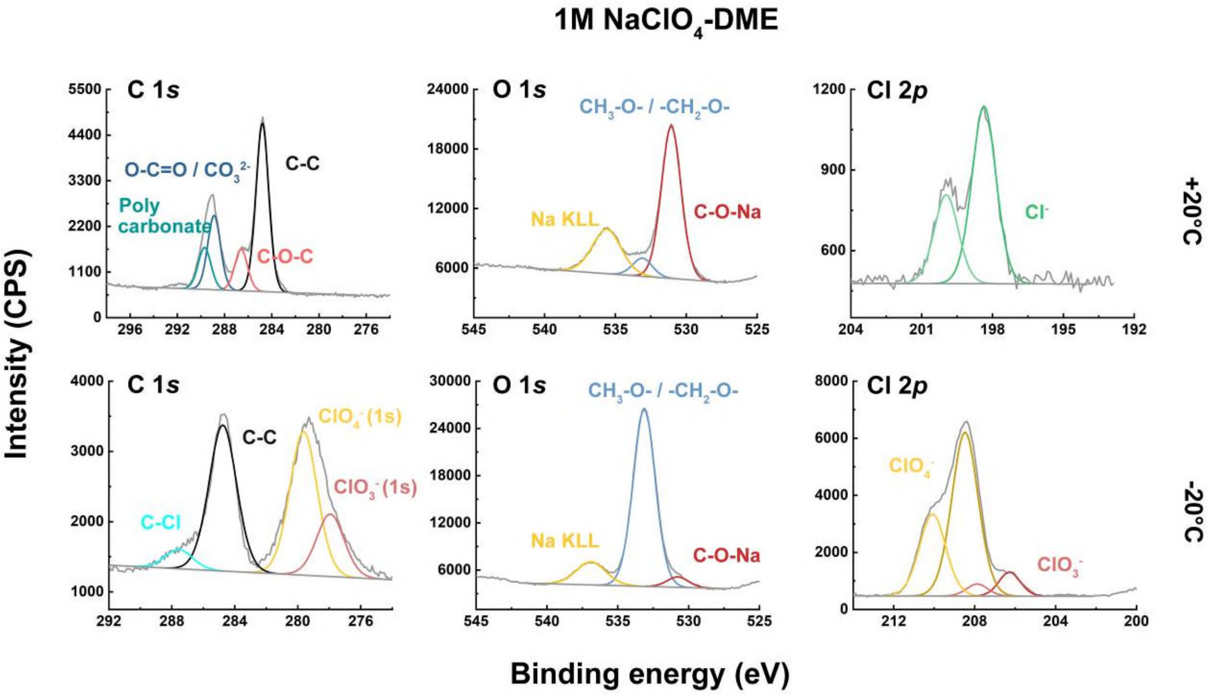

Supplementary Figure 14 | XPS of the Na metal electrode surface after 50 cycles (symmetric Na||Na cells) in 1 M $NaClO_4$-DME electrolyte at +20˚C and −20˚C at a current density of 0.5 mA cm^{-2} with a capacity of 0.5 mAh cm^{-2}. The binding energies of all elements were calibrated with respect to the C 1*s* signal at 284.8 eV. In the C 1*s* profile, besides the peaks of O-C=O (288.7 eV) and C-O-C (286.6 eV), the peaks at 287.6 eV and 289.7 eV are ascribed to C-Cl and polycarbonate; the peaks at 279.6 eV and 277.9 eV correspond to ClO_4^- (Cl 1*s*) and ClO_3^- (Cl 1*s*). In the O 1*s* spectrum, the peaks at 533.3 eV and 531.2 eV correspond to CH_3-O-/-CH_2-O- groups and C-O-Na (e.g., RCH_2ONa) [9], respectively, while the peak at 536.3 eV is attributed to Na KLL. For Cl 2*p* XPS peak-fitting, the $2p_{3/2}$ to $2p_{1/2}$ area ratio is fixed at 2:1 and the doublet separation is 1.60 eV. In the Cl 2*p* profile, the peak at 198.4 eV (based on $2p_{3/2}$) is assigned to Cl^- and the peaks at 208.5 eV and 206.3 eV can be ascribed to ClO_4^- (based on $2p_{3/2}$) and ClO_3^- (based on $2p_{3/2}$) [10-13], respectively.

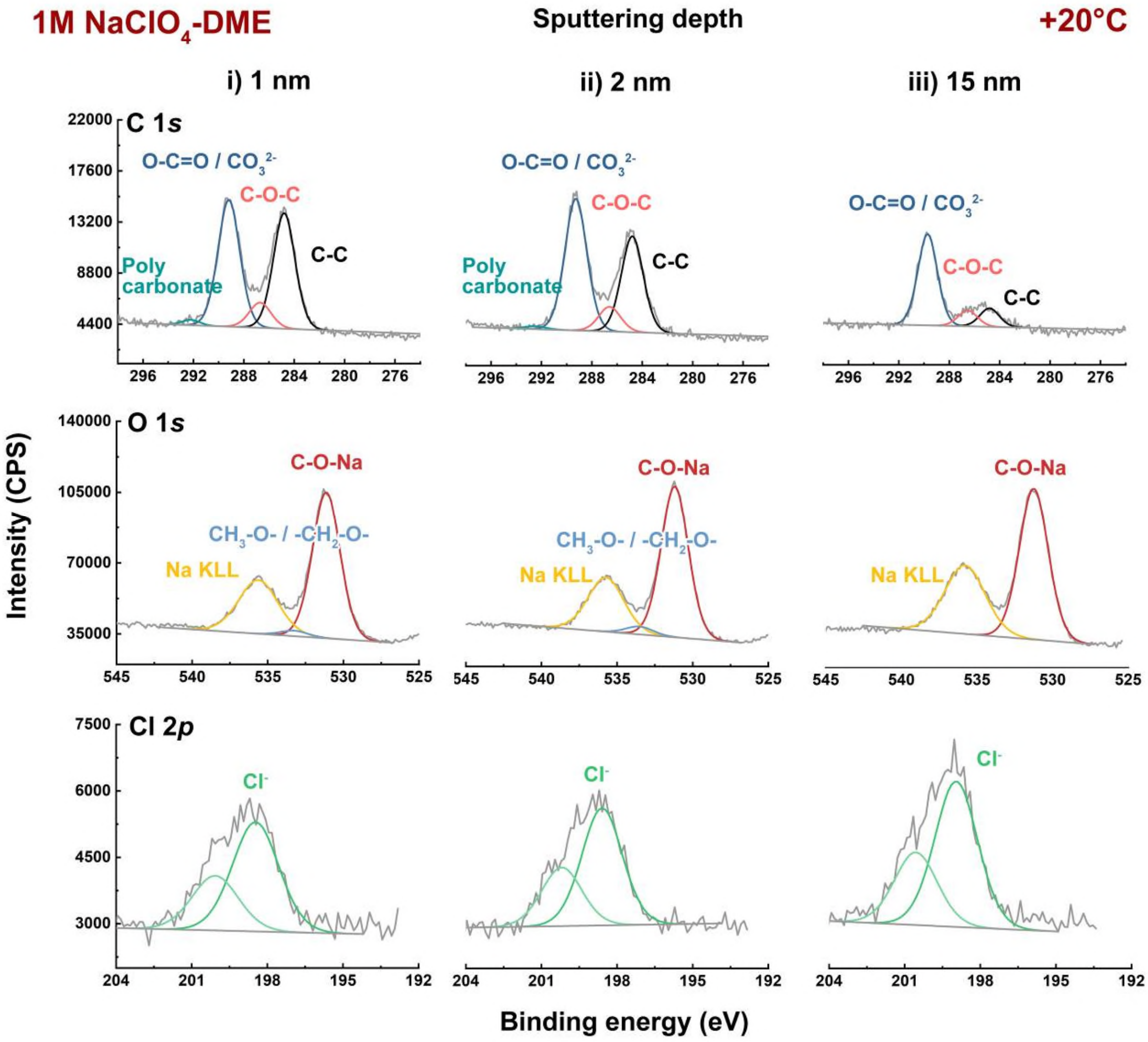

Supplementary Figure 15 | XPS depth profile analysis on the Na metal electrode after 50 cycles (symmetric Na||Na cells) in 1 M $NaClO_4$-DME electrolyte at +20˚C at a current density of 0.5 mA cm^{-2} with a capacity of 0.5 mAh cm^{-2}. XPS peaks are identified in **Supplementary Figure 12**.

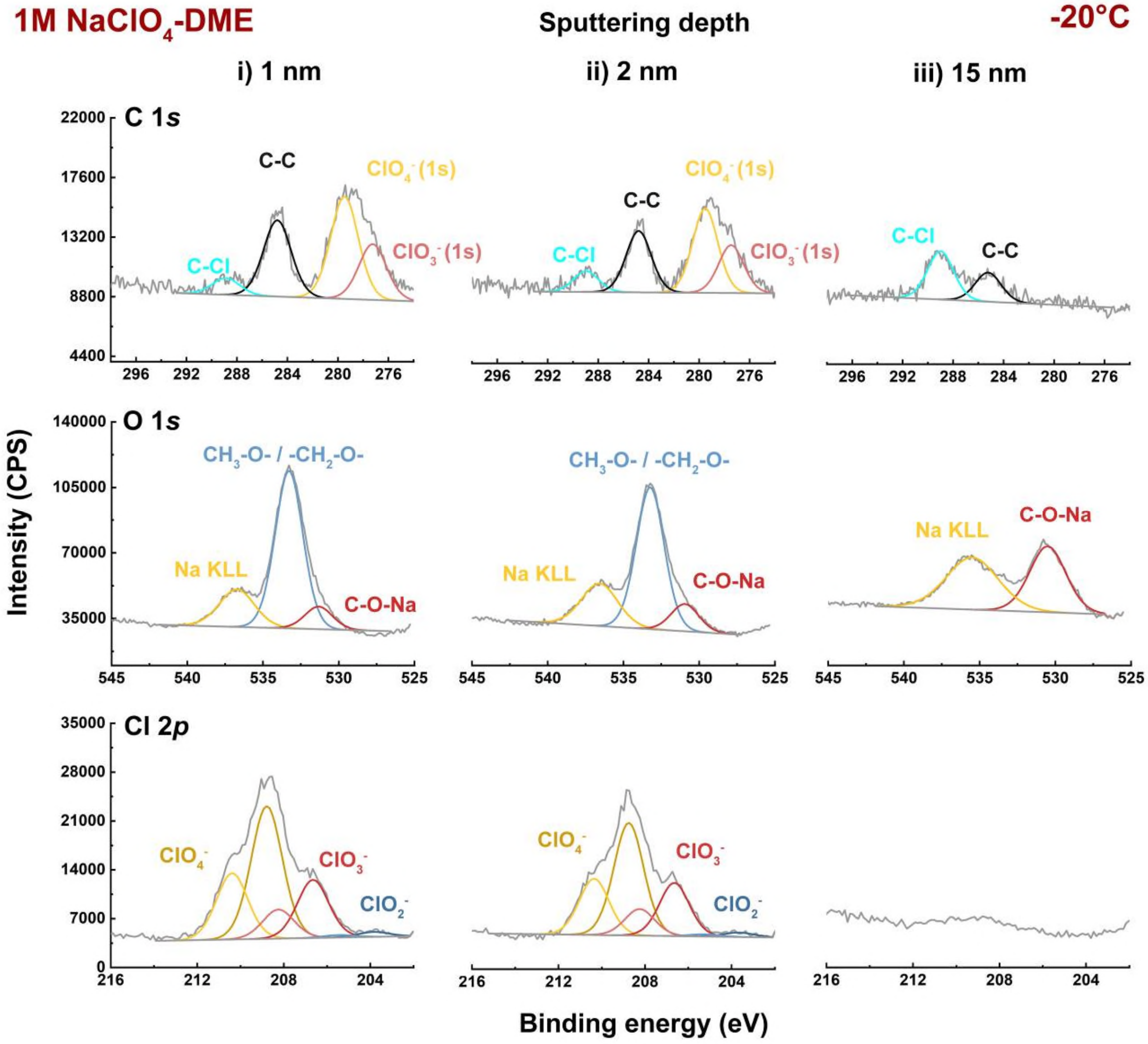

Supplementary Figure 16 | XPS depth profile analysis on the Na metal electrode after 50 cycles (symmetric Na||Na cells) in 1 M $NaClO_4$-DME electrolyte at −20˚C at a current density of 0.5 mA cm^{-2} with a capacity of 0.5 mAh cm^{-2}. In addition to the XPS peaks identified in **supplementary Figure 12**, the peak at 203.8 eV is attributed to ClO_2^- [10-13].

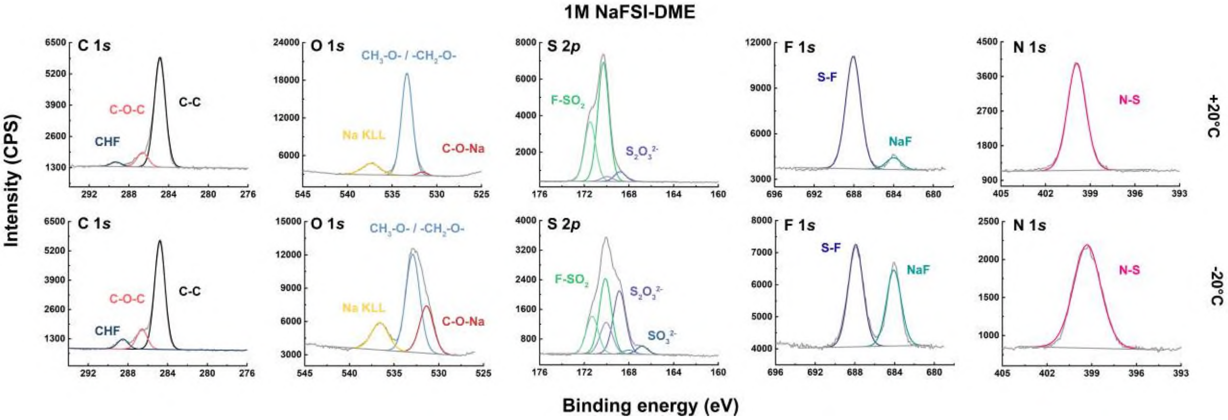

Supplementary Figure 17 | XPS of the Na metal electrode surface after 50 cycles (symmetric Na||Na cells) in 1 M NaFSI-DME electrolyte at +20˚C and −20˚C at a current density of 0.5 mA cm^{-2} with a capacity of 0.5 mAh cm^{-2}. The binding energies of all elements were calibrated with respect to the C 1*s* signal at at 284.8 eV. In the C 1*s* XPS profile, in addition to the peak of C-O-C (286.6 eV), the binding energy at 289.3 eV corresponds to CHF. In the O 1*s* spectrum, the peaks at 533.3 eV and 531.2 eV correspond to CH_3-O-/-CH_2-O- groups[9] and C-O-Na (e.g., RCH_2ONa), respectively, while the peak at 536.3 eV is attributed to Na KLL. For S 2*p* peak-fitting, the $2p_{3/2}$ to $2p_{1/2}$ area ratio is fixed at 2:1 and the doublet separation is 1.18 eV. In S 2*p* XPS, the doublet at 170.2 eV (based on $2p_{3/2}$) can be assigned to F-SO_2 and the peaks at 168.7 eV and 167.2 eV (based on $2p_{3/2}$) are ascribed to $S_2O_3^{2-}$ and SO_3^{2-}. For F 1*s*, the peaks at 687.8 eV and 684.1 eV are assigned to S-F and NaF, respectively. For N 1*s*, the peak at 399.3 eV can be ascribed to N-S[10,14-18].

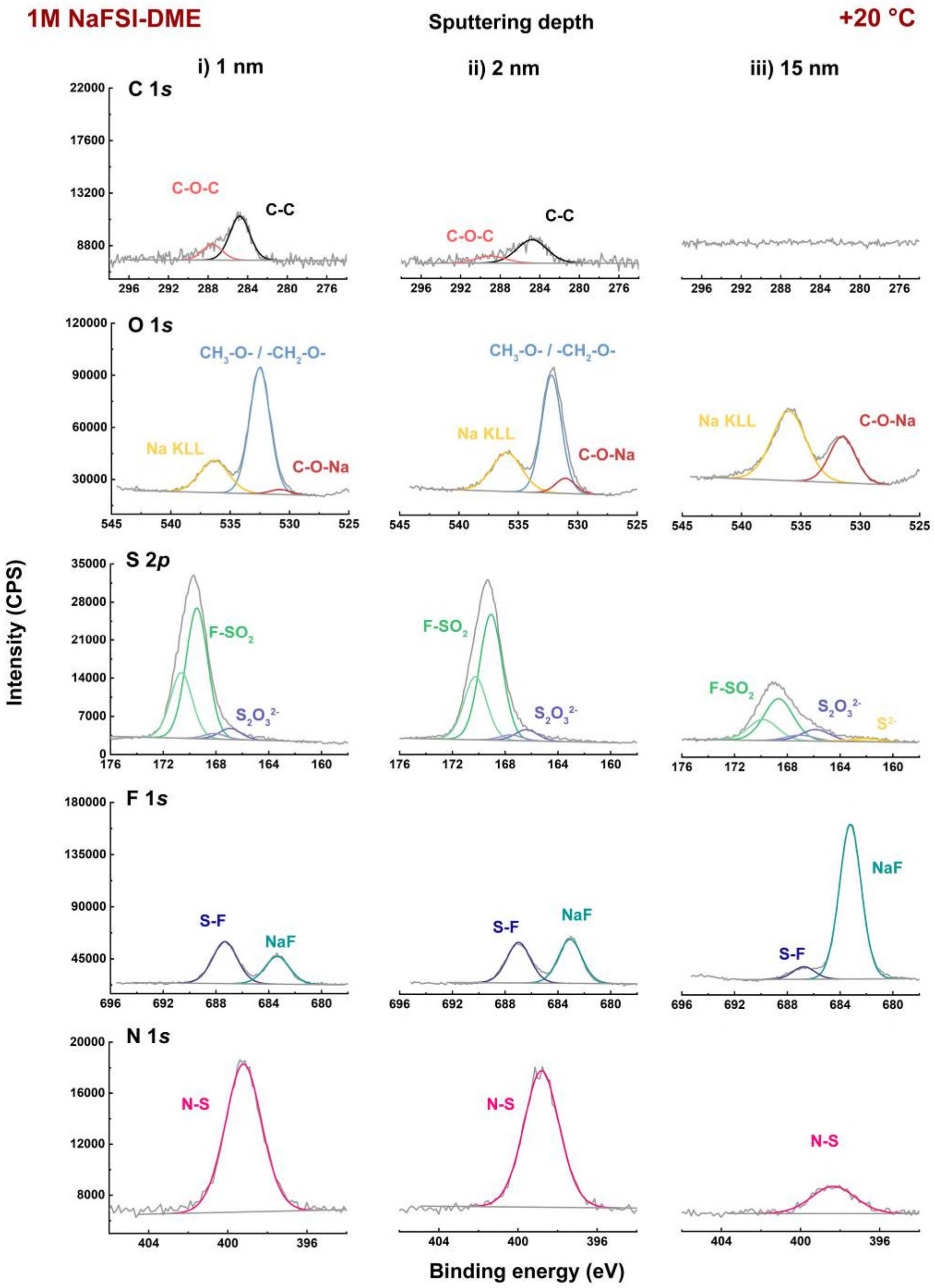

Supplementary Figure 18 | XPS depth profile analysis on the Na metal electrode after 50 cycles (symmetric Na||Na cells) in 1 M NaFSI-DME electrolyte at +20˚C at a current density of 0.5 mA cm^{-2} with a capacity of 0.5 mAh cm^{-2}. Besides the XPS peaks identified in **Supplementary Figure 15**, an additional S 2*p* peak at 161.1 eV (based on the $2p_{3/2}$) is assigned to S^{2-} [10,14-18].

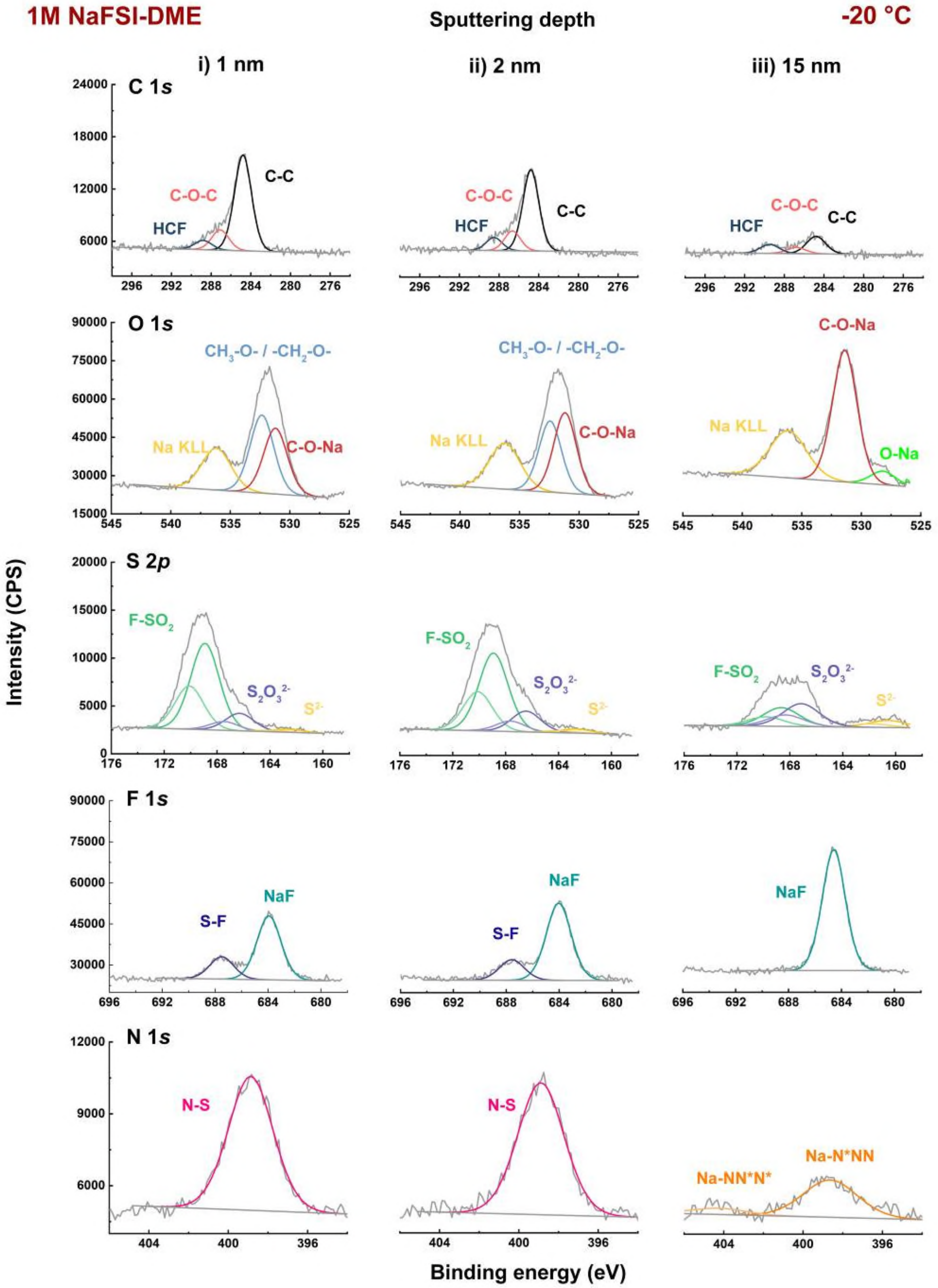

Supplementary Figure 19 | XPS depth profile analysis on the Na metal electrode after 50 cycles (symmetric Na||Na cells) in 1 M NaFSI-DME electrolyte at −20˚C at a current density of 0.5 mA cm^{-2} with a capacity of 0.5 mAh cm^{-2}. Besides the XPS peaks identified in **Supplementary Figure 15**, the N 1*s* XPS peaks at 403.6 eV and 398.5 eV correspond to Na-NN*N* (1N) and Na-N*NN (2N), respectively. Furthermore, the peak at 528.2 eV is attributed to Na_2O in the O 1*s* profile, and the one at 161.1 eV (based on the $2p_{3/2}$) is ascribed to S^{2-} in the S 2*p* profile[10,14-18].

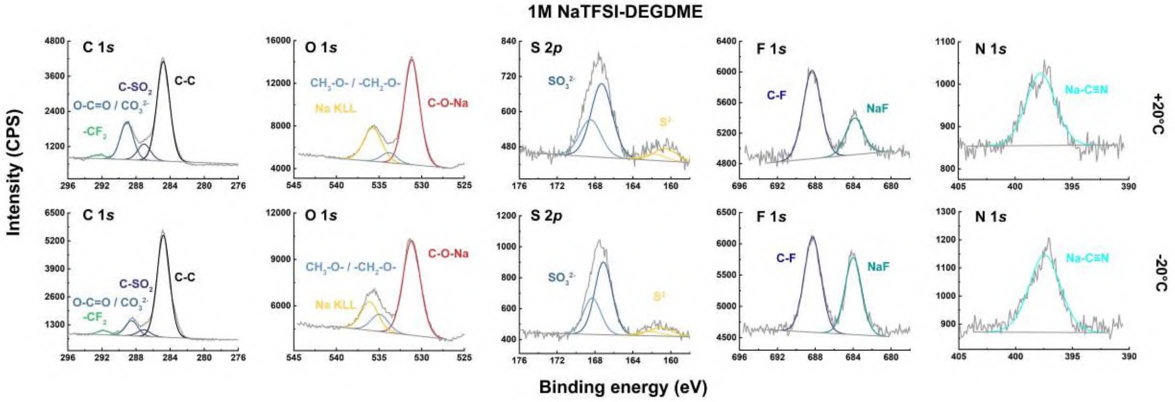

Supplementary Figure 20 | XPS of the Na metal electrode surface (symmetric Na||Na cells) in 1 M NaTFSI-DEGDME electrolyte at +20˚C and −20˚C at a current density of 0.5 mA cm^{-2} with a capacity of 0.5 mAh cm^{-2}. Note that the cell with 1 M NaTFSI-DEGDME at +20˚C failed early at the 16th cycles, and the cell with 1 M NaTFSI-DEGDME at −20˚C was stopped after 50 cycles. The binding energies of all elements were calibrated with respect to the C 1*s* signal at 284.8 eV. In the C 1*s* XPS profile, besides the peak of O-C=O (288.7 eV), the peaks at 287.2 eV and 292.3 eV are attributed to C-SO_2 and -CF_2, respectively. In the O 1*s* spectrum, the peaks at 533.3 eV and 531.2 eV correspond to CH_3-O-/-CH_2-O- groups and C-O-Na (e.g., RCH_2ONa) [9], respectively, while the peak at 536.3 eV is attributed to Na KLL. For S 2*p* peak-fitting, the $2p_{3/2}$ to $2p_{1/2}$ area ratio is fixed at 2:1 and the doublet separation is 1.18 eV. In S 2*p* XPS, the doublets at 167.2 eV and 161.1 eV (based on $2p_{3/2}$) are ascribed to SO_3^{2-} and S^{2-}. For F 1*s*, the peaks at 689.3 eV and 684.1 eV are assigned to C-F and NaF. For N 1*s*, the peak at 397.4 eV is assigned to NaCN[10,14-18].

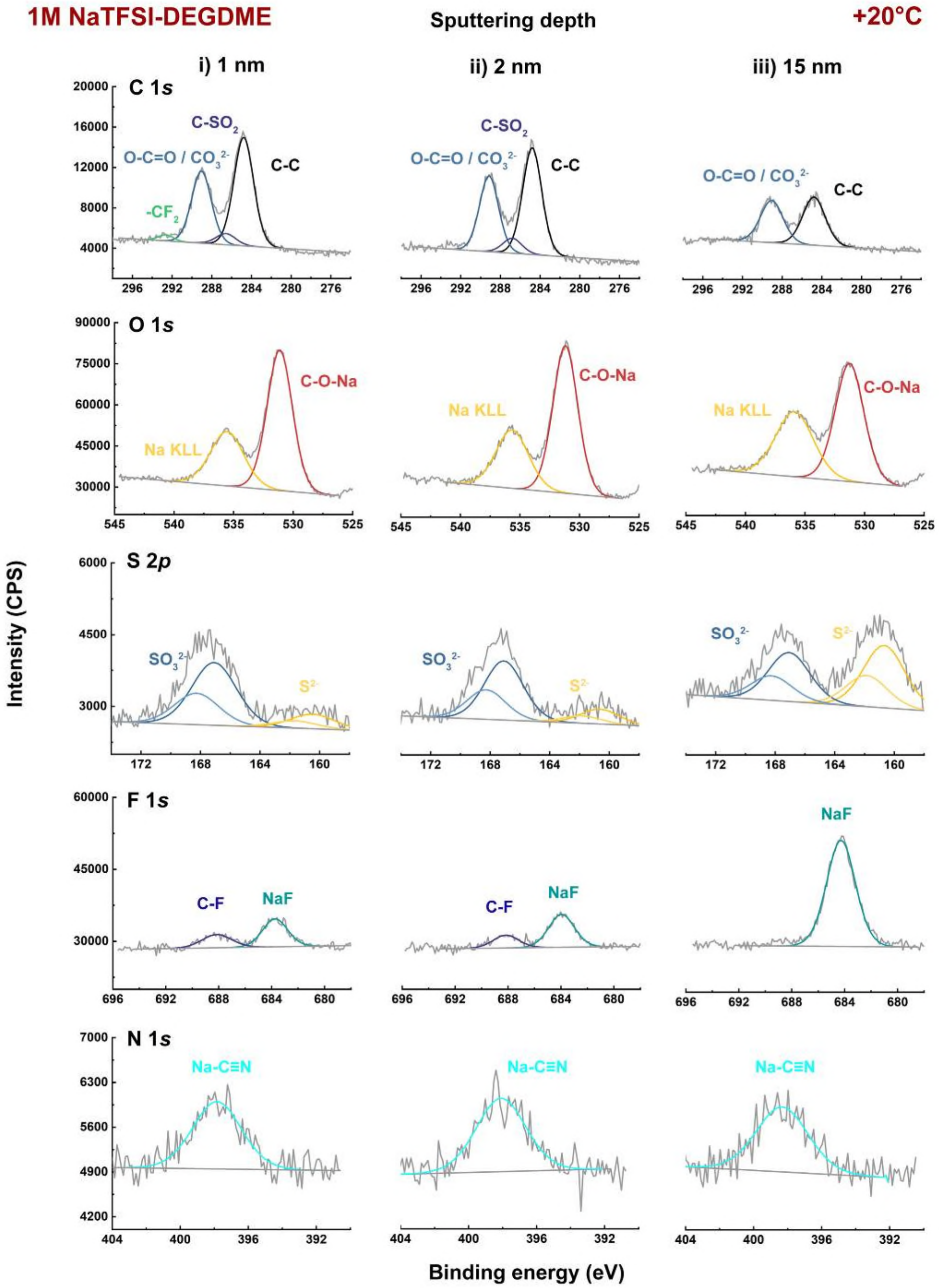

Supplementary Figure 21 | XPS depth profile on the Na metal electrode after 15 cycles (symmetric Na||Na cells) in 1 M NaTFSI-DEGDME electrolyte at +20˚C at a current density of 0.5 mA cm^{-2} with a capacity of 0.5 mAh cm^{-2}.

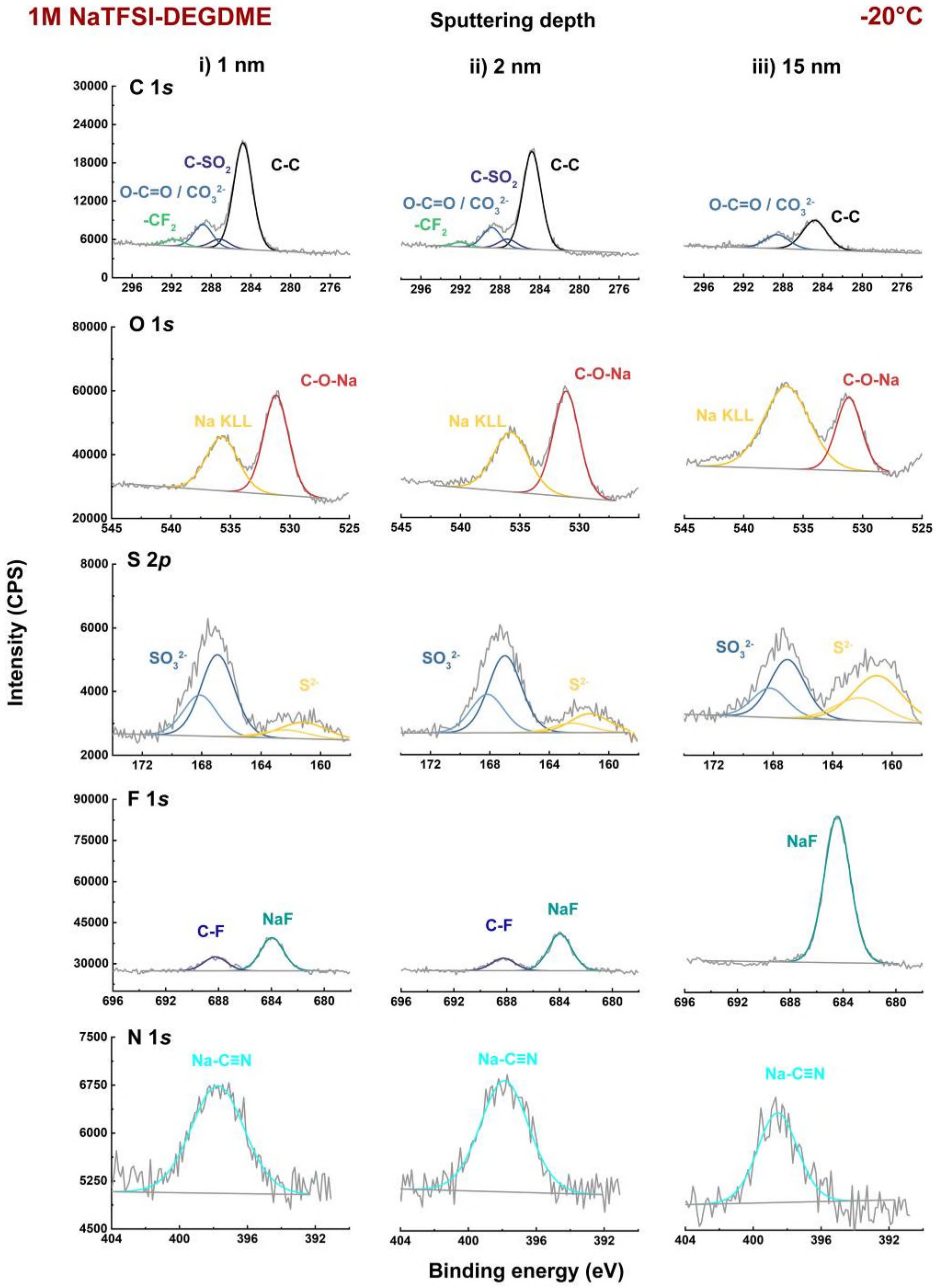

Supplementary Figure 22 | XPS depth profile on the Na metal electrode after 50 cycles (symmetric Na||Na cells) in 1 M NaTFSI-DEGDME electrolyte at −20˚C at a current density of 0.5 mA cm^{-2} with a capacity of 0.5 mAh cm^{-2}.

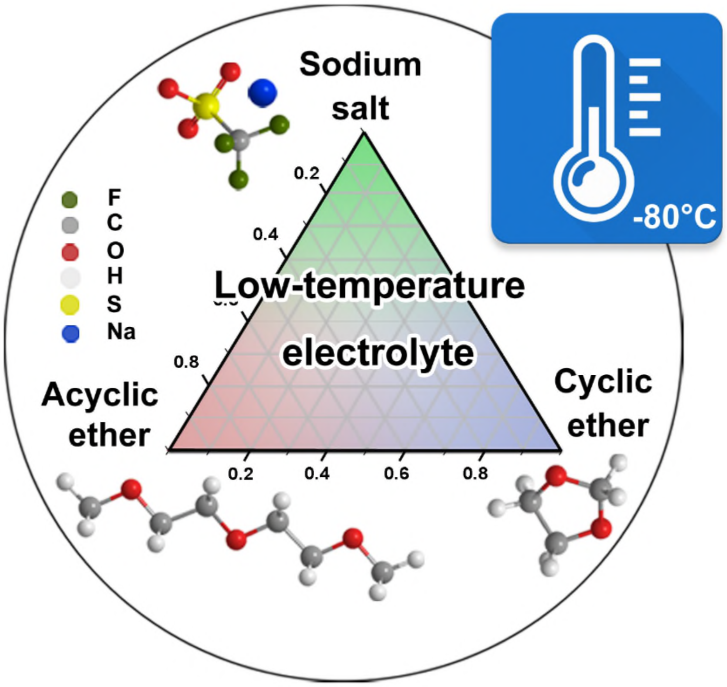

Supplementary Figure 23 | Illustration of tailoring low-temperature electrolytes comprising a sodium salt (NaOTf) and binary solvents of an acyclic ether (DEGDME) and a cyclic ether (DOL).

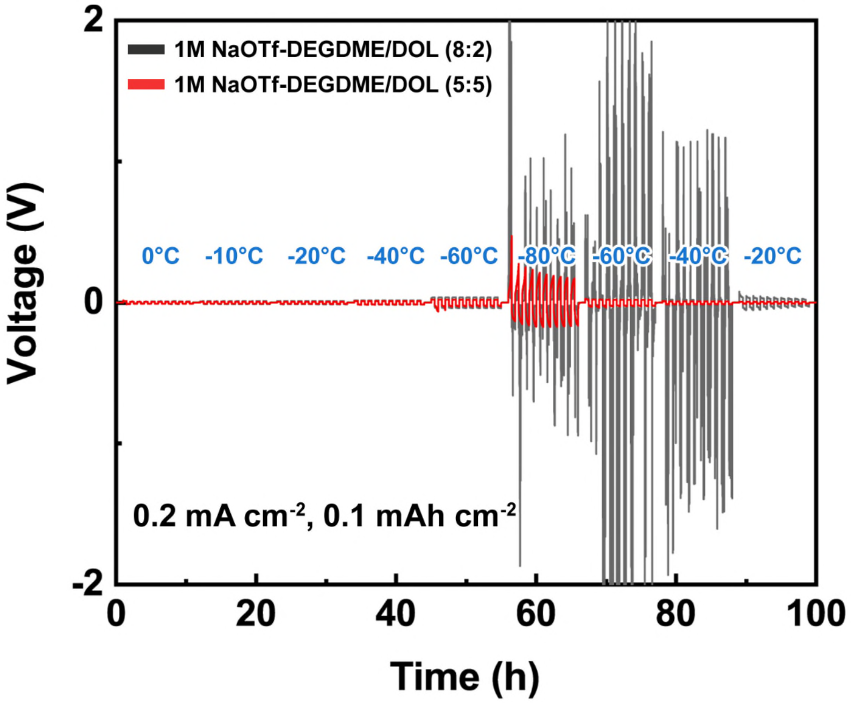

Supplementary Figure 24 | Temperature-dependent galvanostatic cycling of Na||Na symmetric cells in 1 M NaOTf-DEGDME/DOL (8:2 and 5:5 in volume ratio) at a current density of 0.2 mA cm^{-2} with a capacity of 0.1 mAh cm^{-2}.

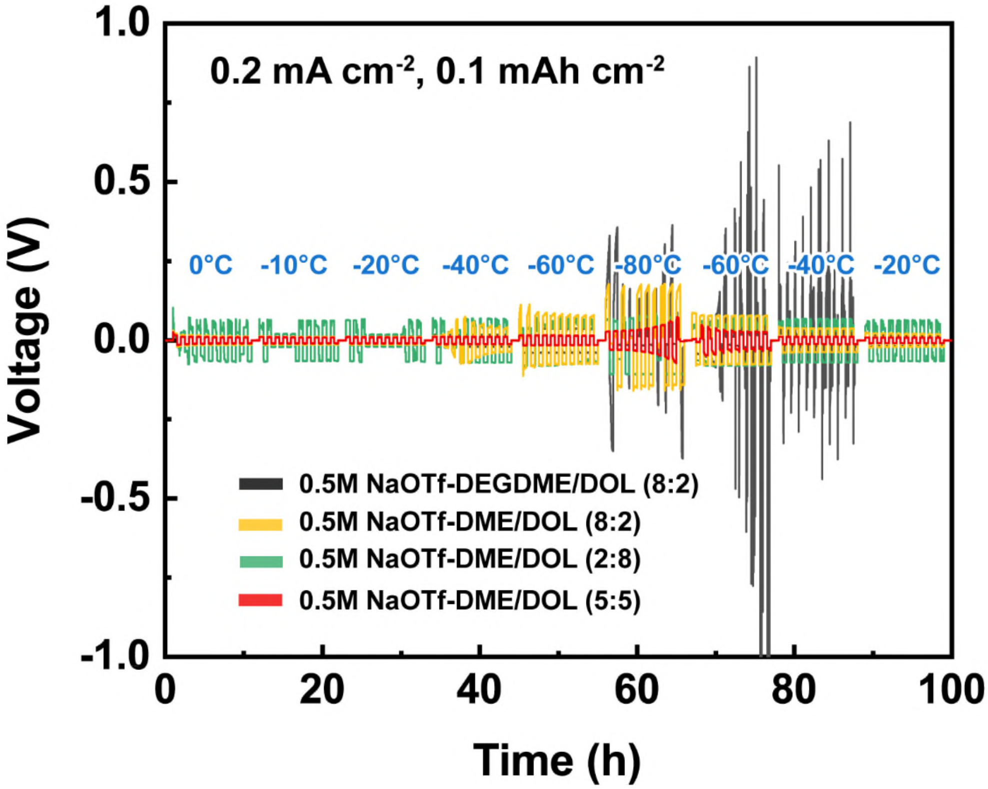

Supplementary Figure 25 | Temperature-dependent galvanostatic cycling of Na||Na symmetric cells in 0.5 M NaOTf-DEGDME/DOL (8:2 in volume ratio) and 0.5 M NaOTf-DME/DOL (8:2, 5:5 and 2:8 in volume ratio) at a current density of 0.2 mA cm^{-2} with a capacity of 0.1 mAh cm^{-2}.

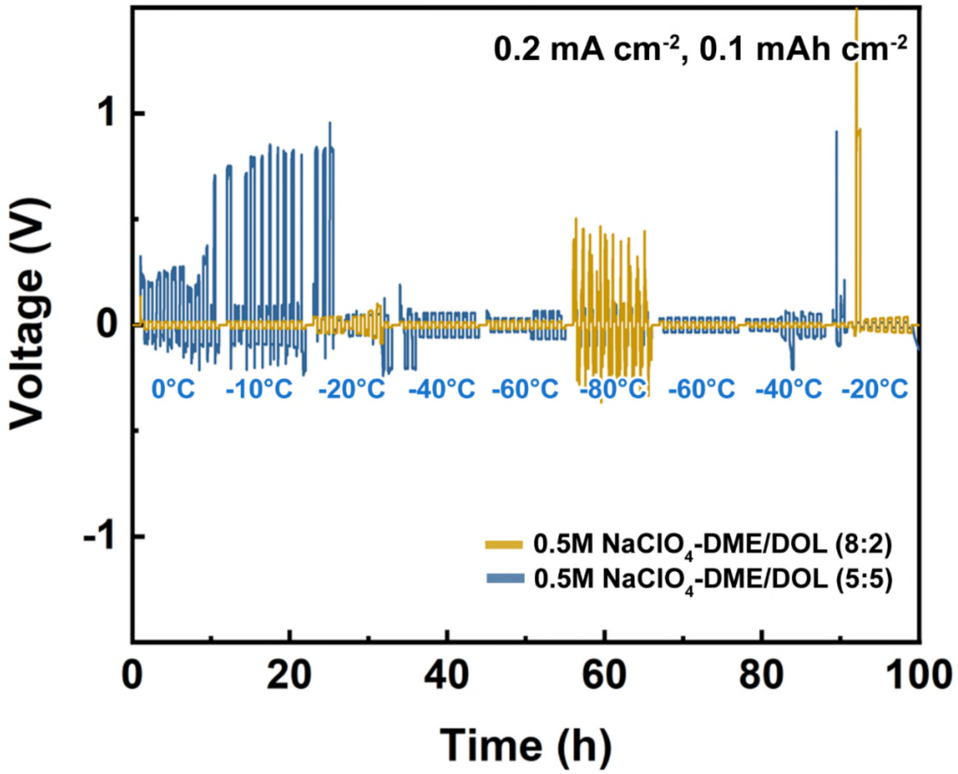

Supplementary Figure 26 | Temperature-dependent galvanostatic cycling of Na||Na symmetric cells in 0.5 M $NaClO_4$-DME/DOL (8:2 and 5:5 in volume ratio) at a current density of 0.2 mA cm^{-2} with a capacity of 0.1 mAh cm^{-2}.

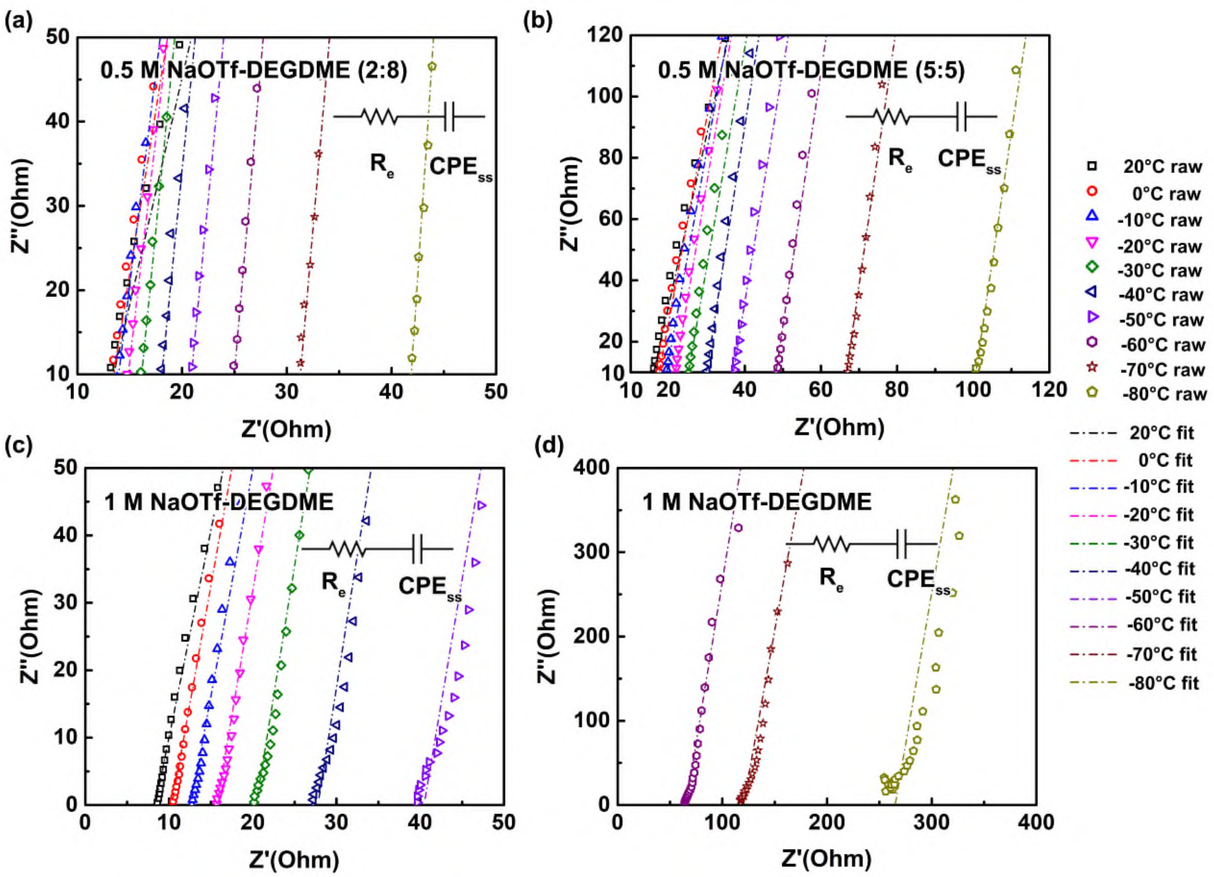

Supplementary Figure 27 | EIS spectra of **a,** 0.5 M NaOTf-DEGDME/DOL (2:8), **b,** 0.5 M NaOTf-DEGDME/DOL (5:5), **c and d,** 1 M NaOTf-DEGDME electrolyte solutions using the symmetric stainless steel||stainless steel cells across a range of temperatures (EIS spectra for resistance evaluation shown in Figure 4b and c). The insets present the equivalent circuits used for EIS fitting[7,8]: R_e, total resistance of the electrolyte; CPE_{ss}, capacitance of the blocking electrodes (stainless steel).

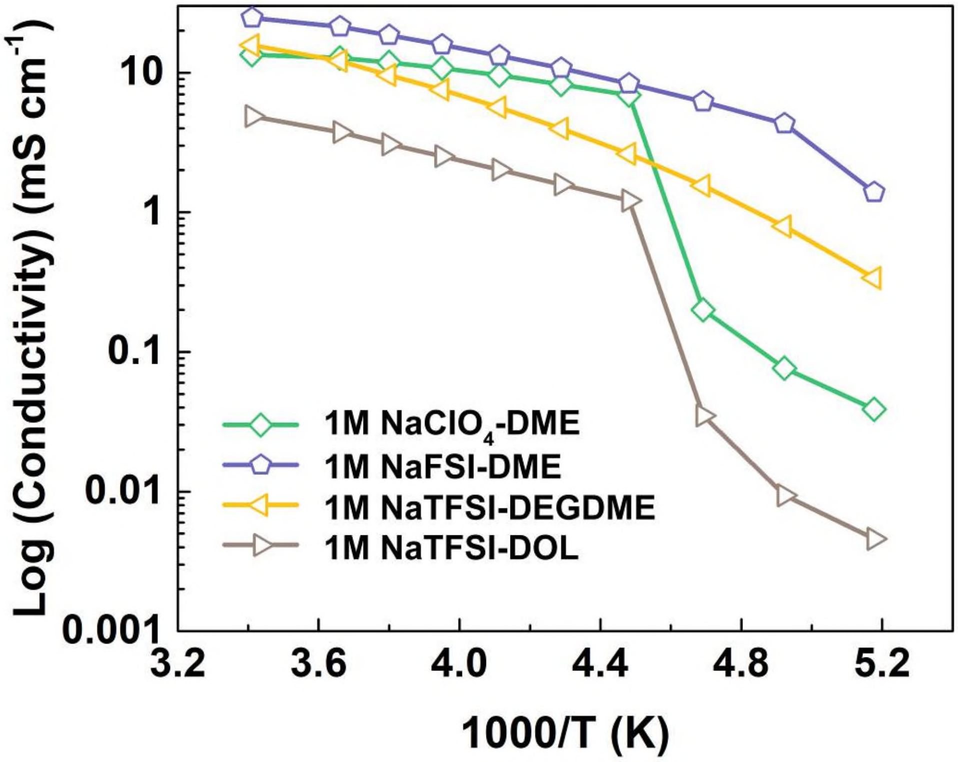

Supplementary Figure 28 | Temperature dependent ionic conductivity of $NaClO_4$-DME, NaFSI-DME, NaTFSI-DEGDME, and NaTFSI-DOL electrolyte solution (all in 1 M salt concentration).

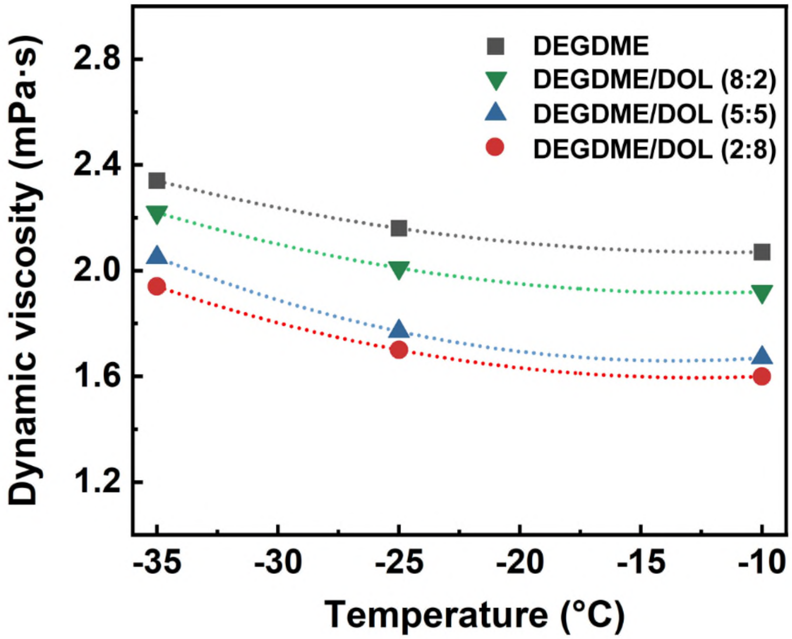

Supplementary Figure 29 | Temperature-dependent dynamic viscosity of DEGDME and DEGDME/DOL (8:2, 5:5 and 2:8 in volume ratio).

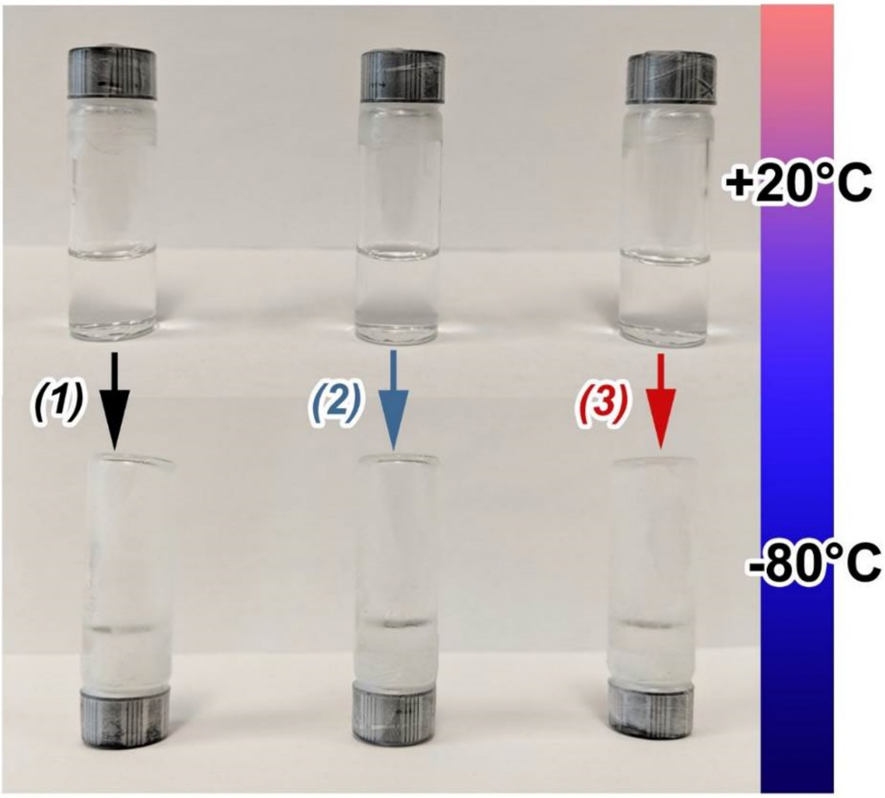

Supplementary Figure 30 | Photographic pictures of *(1)* 1 M NaOTf-DEGDME, *(2)* 0.5 M NaOTf-DEGDME/DOL (5:5) and *(3)* 0.5 M NaOTf-DEGDME/DOL (2:8) after storing at +20˚C and −80˚C for 24 hours**.** All three electrolytes are stable at −80˚C, showing no precipitation of salts.

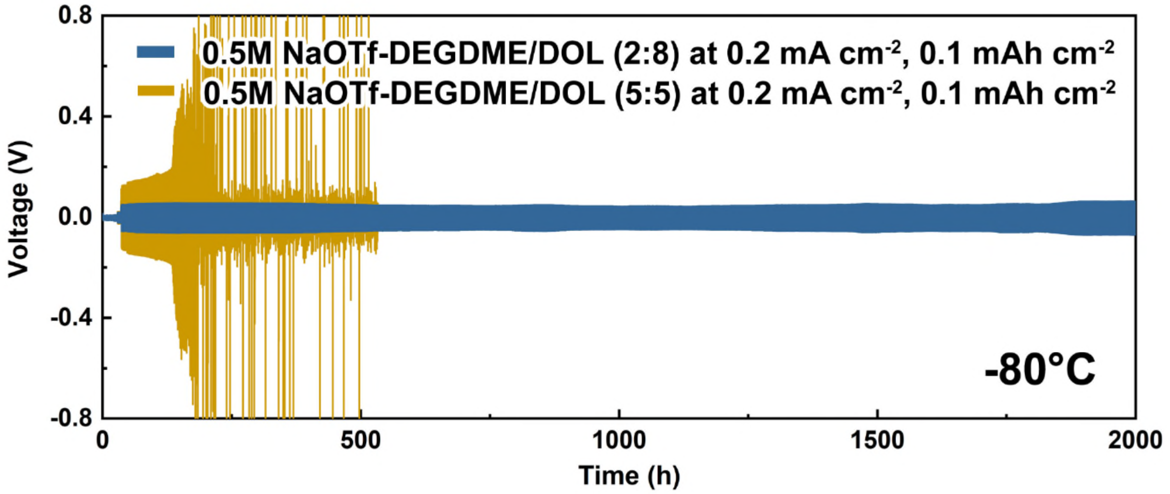

Supplementary Figure 31 | Galvanostatic cycling of Na||Na symmetric cells in 0.5 M NaOTf-DEGDME/DOL (2:8 and 5:5 in volume ratio) at 0.2 mA cm^{-2} and 0.1 mAh cm^{-2} at −80˚C. Note that initial stepwise temperature drop was carried out to stabilize the cells.

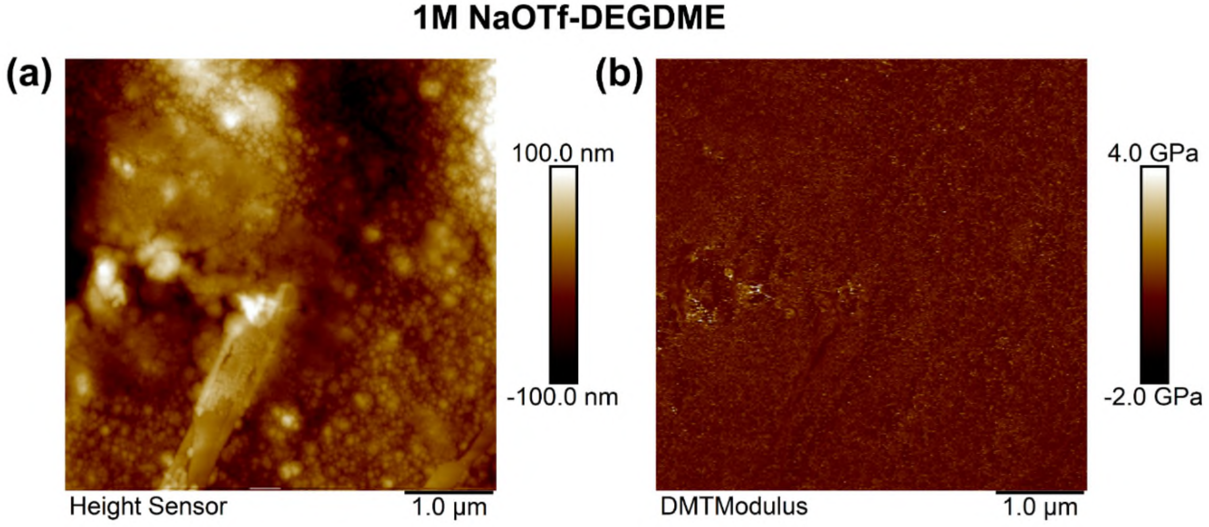

Supplementary Figure 32 | AFM characterization of SEI formed on copper foil after Na plating/stripping at 0.5 mA cm^{-2} and 0.5 mAh cm^{-2} with a cut-off voltage of 1.5 V after the 1st cycle (Cu foil is fully desodiated) in 1 M NaOTf-DEGDME electrolyte at −40°C. **a**, AFM topography of SEI formed on copper foil. **b**, Young's modulus (determined by AFM) of SEI formed on copper foil (average Young's modulus: ~0.5 GPa).

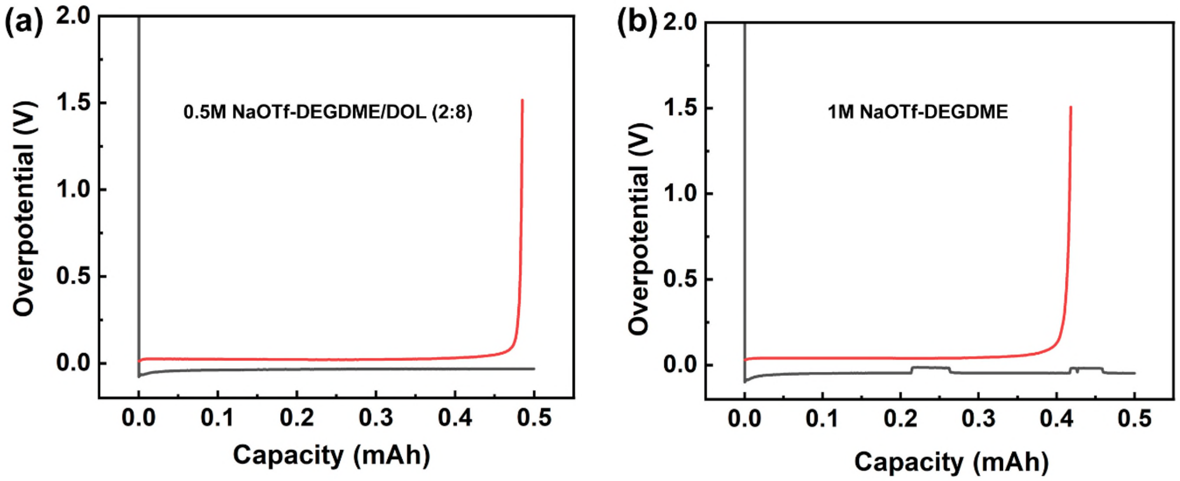

Supplementary Figure 33 | Na plating/stripping profile of Na||Cu asymmetric cells at 0.5 mA cm^{-2} and 0.5 mAh cm^{-2} with a cut-off voltage of 1.5 V for the 1st cycle at −40˚C. **a**, 0.5 M NaOTf-DEGDME/DOL (2:8) electrolyte. **b**, 1 M NaOTf-DEGDME electrolyte.

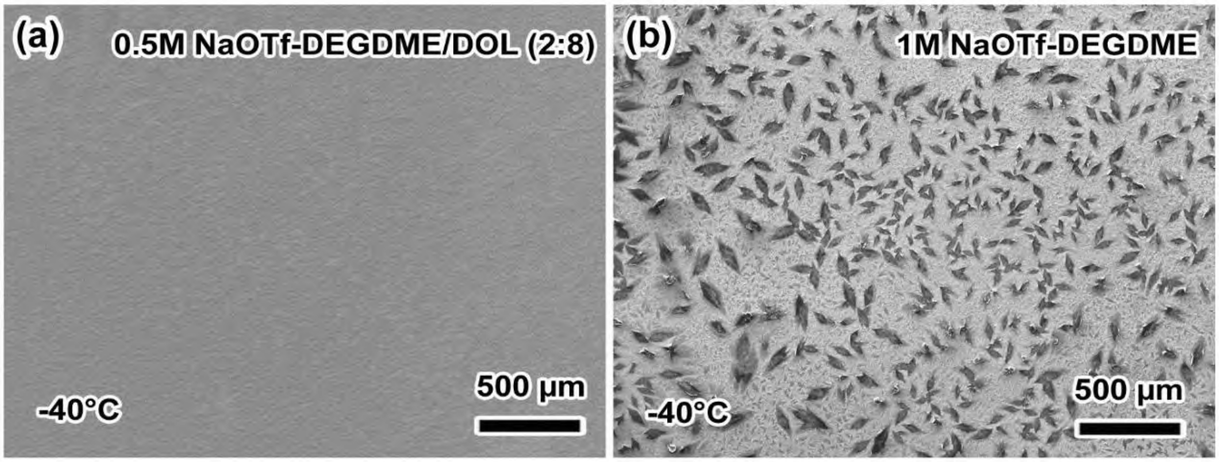

Supplementary Figure 34 | SEM image of Cu surface after Na plating/stripping (Na||Cu asymmetric cells) for the 1st cycle at 0.5 mA cm^{-2} and 0.5 mAh cm^{-2} with a cut-off voltage of 1.5 V at −40˚C. **a**, 0.5 M NaOTf-DEGDME/DOL (2:8) electrolyte. **b**, 1 M NaOTf-DEGDME electrolyte.

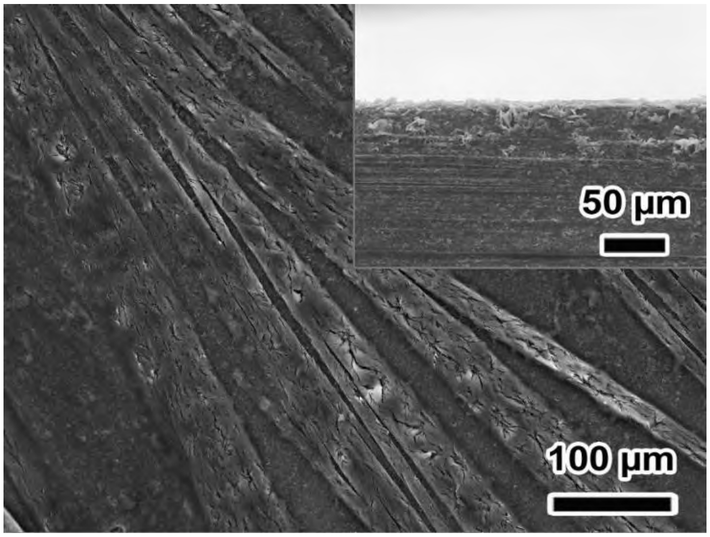

Supplementary Figure 35 | SEM of a Na metal electrode surface after 50 cycles (symmetric Na||Na cells) at 0.5 mA cm^{-2} and 0.5 mAh cm^{-2} in 0.5 M NaOTf DEGDME/DOL (5:5) at −80°C (Inset: corresponding cross-sectional SEM image).

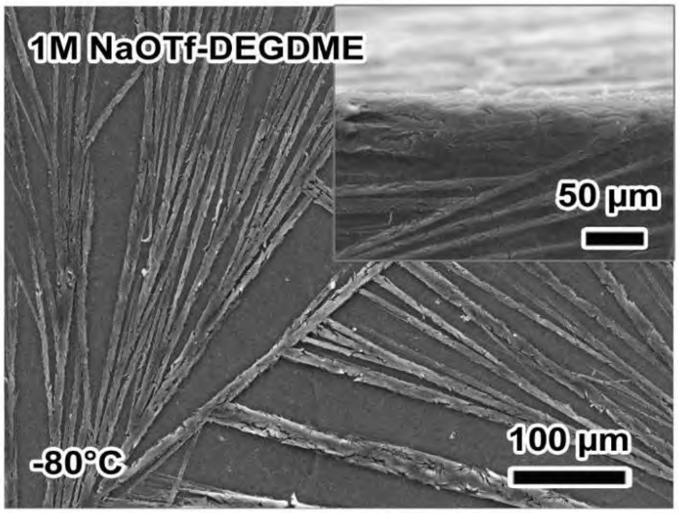

Supplementary Figure 36 | SEM of a Na metal electrode surface after 50 cycles (symmetric Na||Na cells) at 0.5 mA cm^{-2} and 0.5 mAh cm^{-2} in 1 M NaOTf DEGDME at −80°C (Inset: corresponding cross-sectional SEM image).

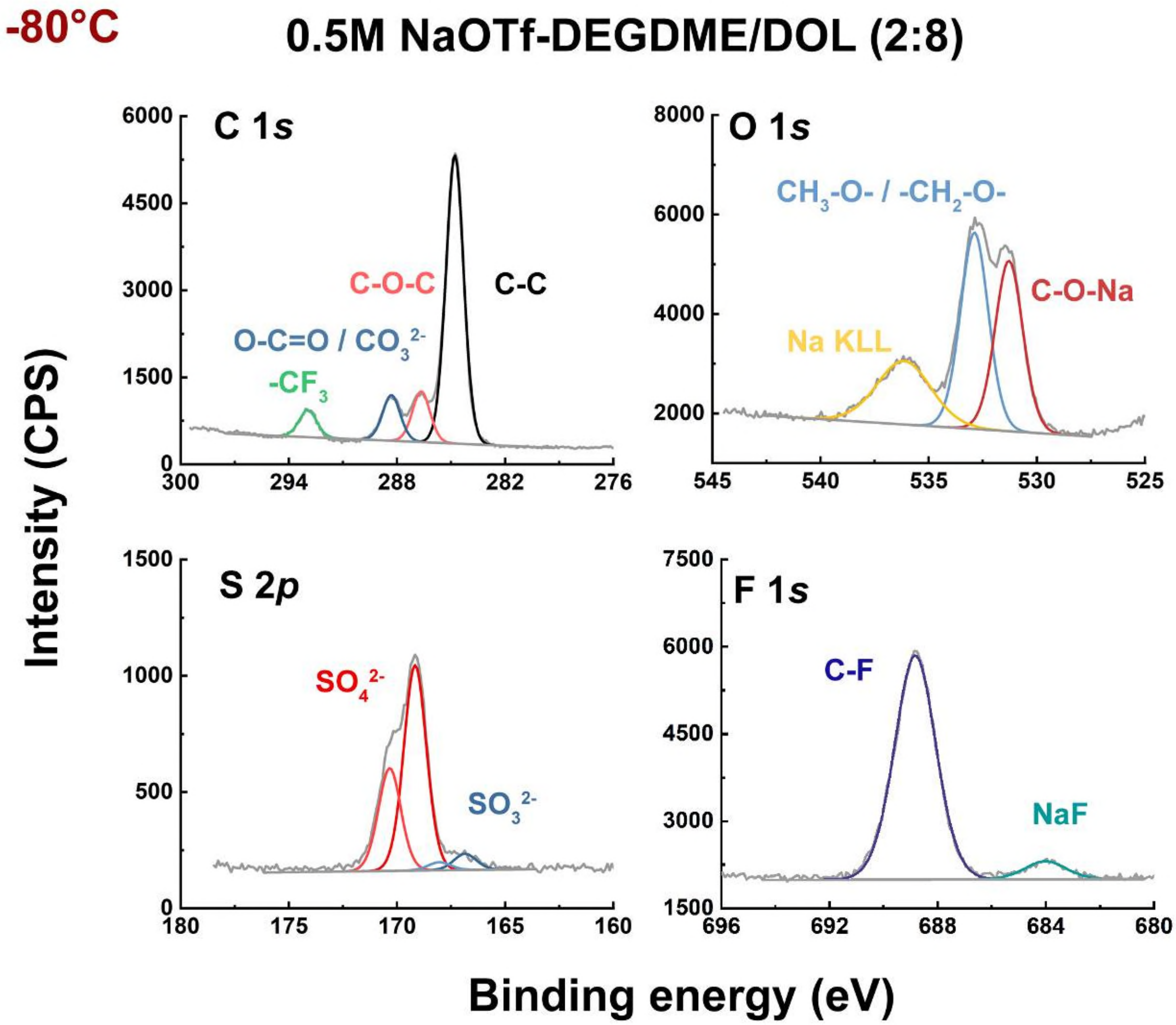

Supplementary Figure 37 | XPS of S 2*p*, C 1*s*, O 1*s* and F 1*s* profiles of Na metal electrode surface after 50 cycles (symmetric Na||Na cells) at a current density of 0.5 mA cm^{-2} with a capacity of 0.5 mAh cm^{-2} in 0.5 M NaOTf-DEGDME/DOL (2:8 in volume ratio) electrolyte at −80˚C**.**

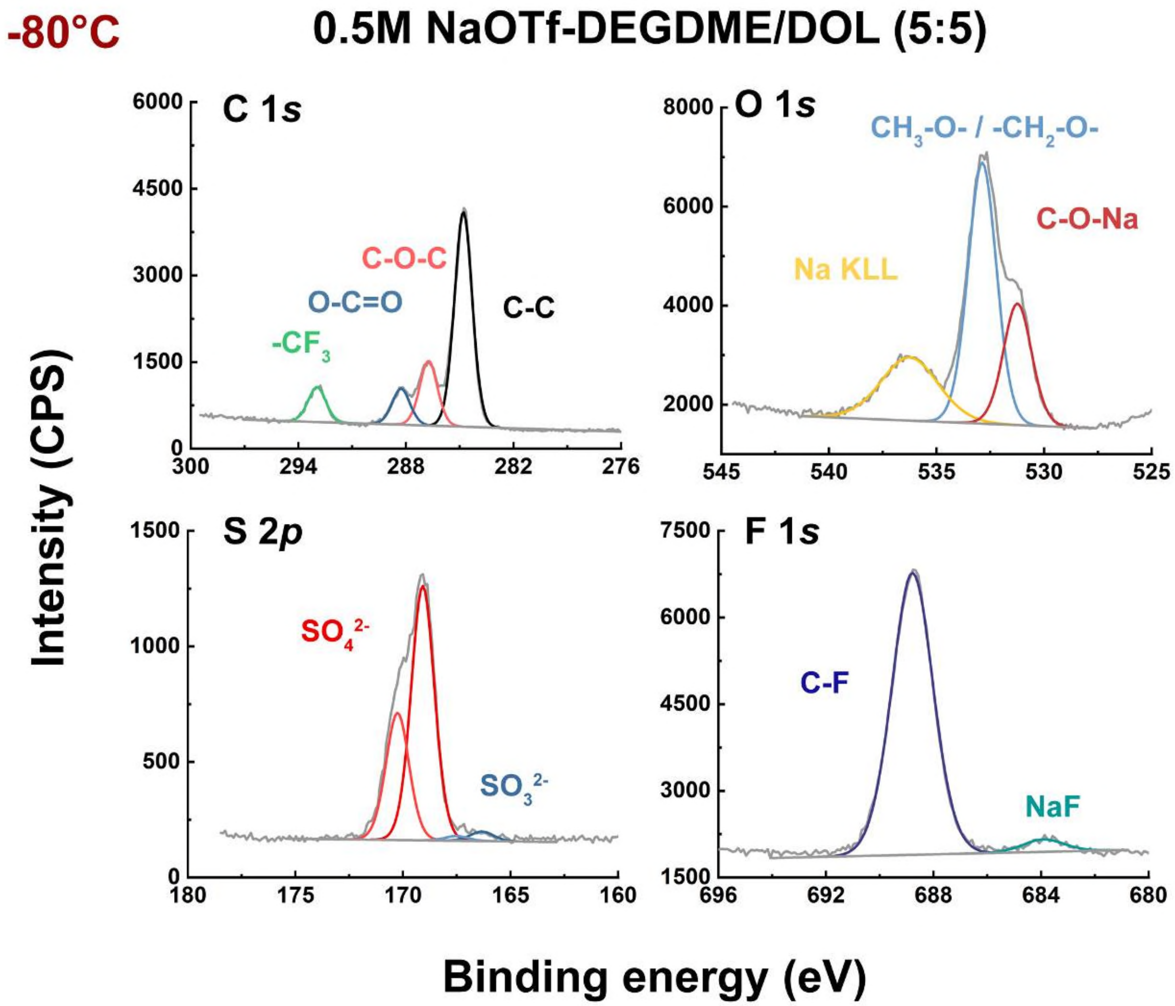

Supplementary Figure 38 | XPS of S 2*p*, C 1*s*, O 1*s* and F 1*s* profiles on Na metal electrode surface after 50 cycles (symmetric Na||Na cells) at a current density of 0.5 mA cm^{-2} with a capacity of 0.5 mAh cm^{-2} in 0.5 M NaOTf-DEGDME/DOL (5:5 in volume ratio) electrolyte at −80˚C**.**

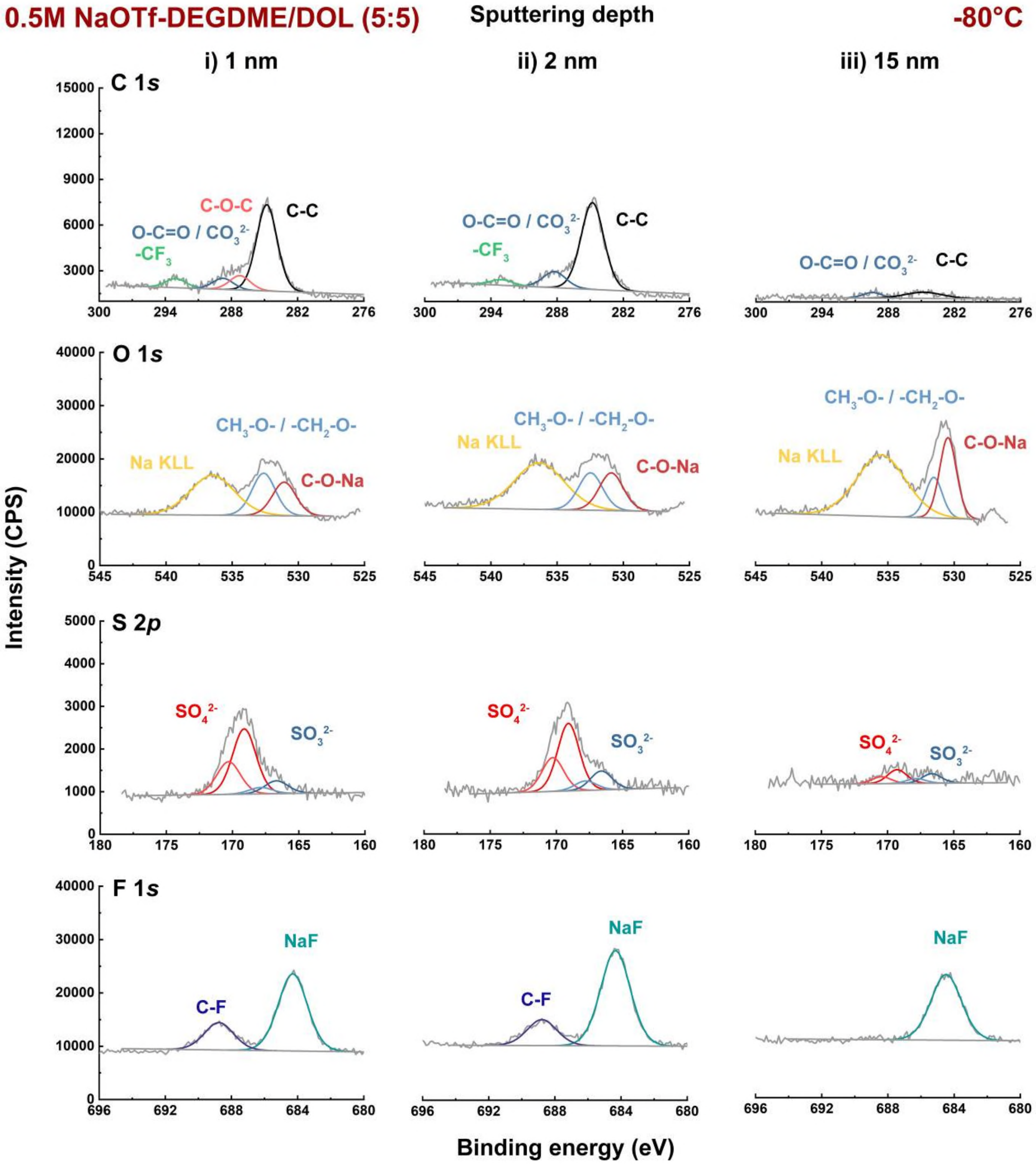

Supplementary Figure 39 | XPS depth profile analysis on Na metal electrode after 50 cycles (symmetric Na||Na cells) at a current density of 0.5 mA cm^{-2} with a capacity of 0.5 mAh cm^{-2} in 0.5 M NaOTf-DEGDME/DOL (5:5) electrolyte at −80˚C.

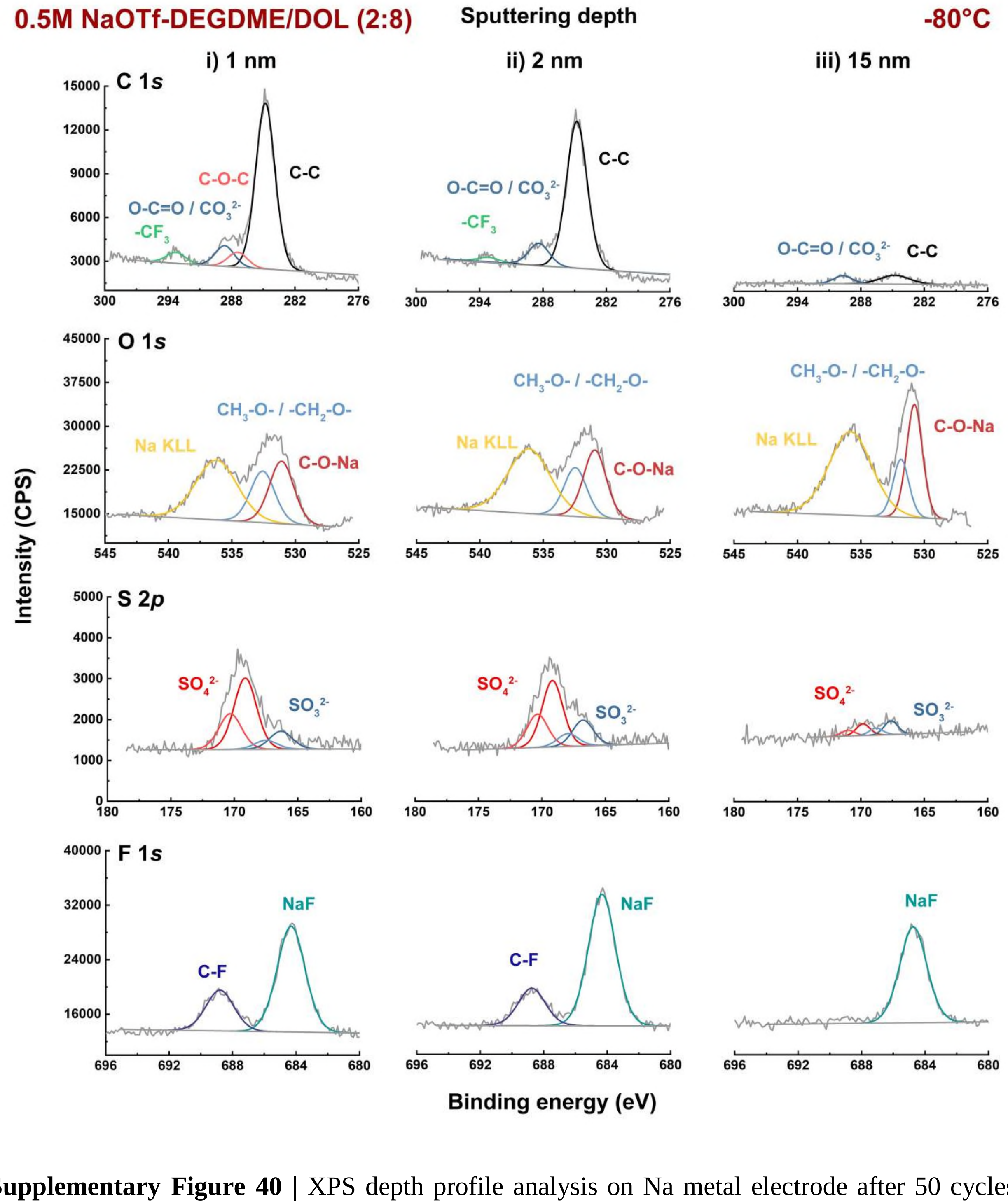

Supplementary Figure 40 | XPS depth profile analysis on Na metal electrode after 50 cycles (symmetric Na||Na cells) at a current density of 0.5 mA cm^{-2} with a capacity of 0.5 mAh cm^{-2} in 0.5 M NaOTf-DEGDME/DOL (2:8) at −80˚C**.**

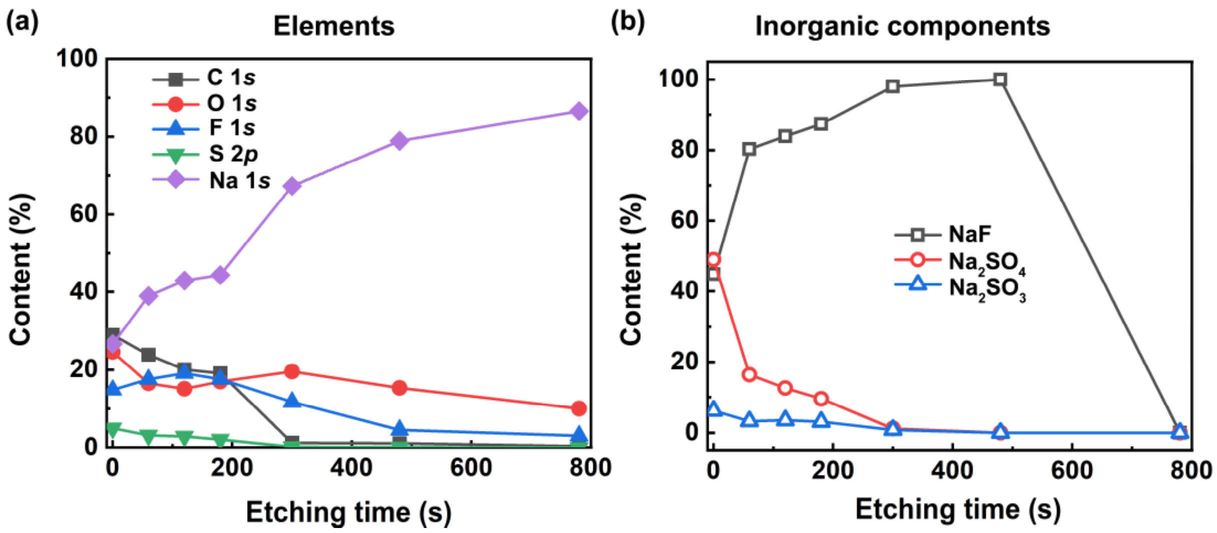

Supplementary Figure 41 | Contents of elements and inorganic components determined by ex situ postmortem XPS depth profiling of the Na metal electrodes (symmetric Na||Na cells) after 50 cycles at a current density of 0.5 mA cm^{-2} with a capacity of 0.5 mAh cm^{-2} in 0.5 M NaOTf-DEGDME/DOL (5:5) at −80˚C. **a,** Content of C 1*s*, O 1*s*, F 1*s*, S 2*p* and Na 1*s* elements in SEI. **b,** Content of main SEI inorganic components including NaF, Na_2SO_4 and Na_2SO_3**.**

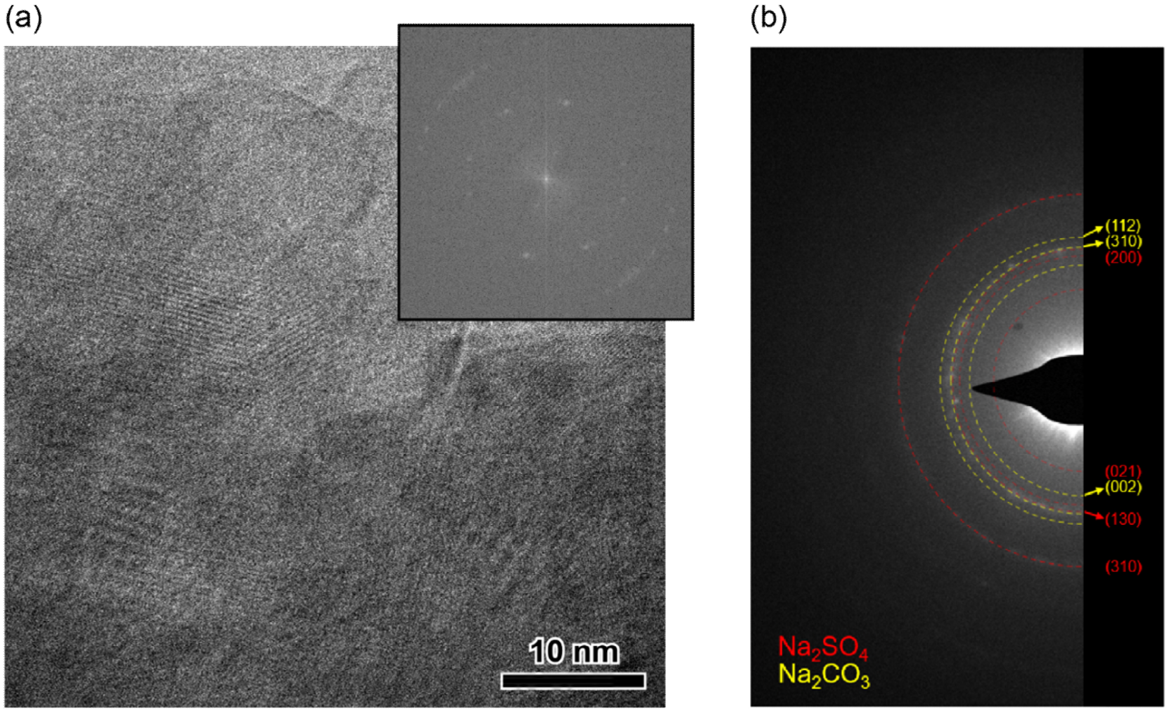

Supplementary Figure 42 | SEI formed after the 1st cycle (after Na is fully stripped) in Na||Cu TEM grid cells at a current density of 0.25 mA cm^{-2} with a capacity of 0.5 mAh cm^{-2} at −40°C using the single-solvent electrolyte of 1 M NaOTf-DEGDME. **a**, High-resolution, cryo-TEM micrograph with the FFT shown in the inset. **b**, Selected-area electron diffraction pattern of the SEI with the positions of the rings expected to be most intense from Na_2SO_4 and Na_2CO_3 overlaid. The lattice fringes are generally consistent with the dominant lattice spacings of Na_2SO_4 and Na_2CO_3.

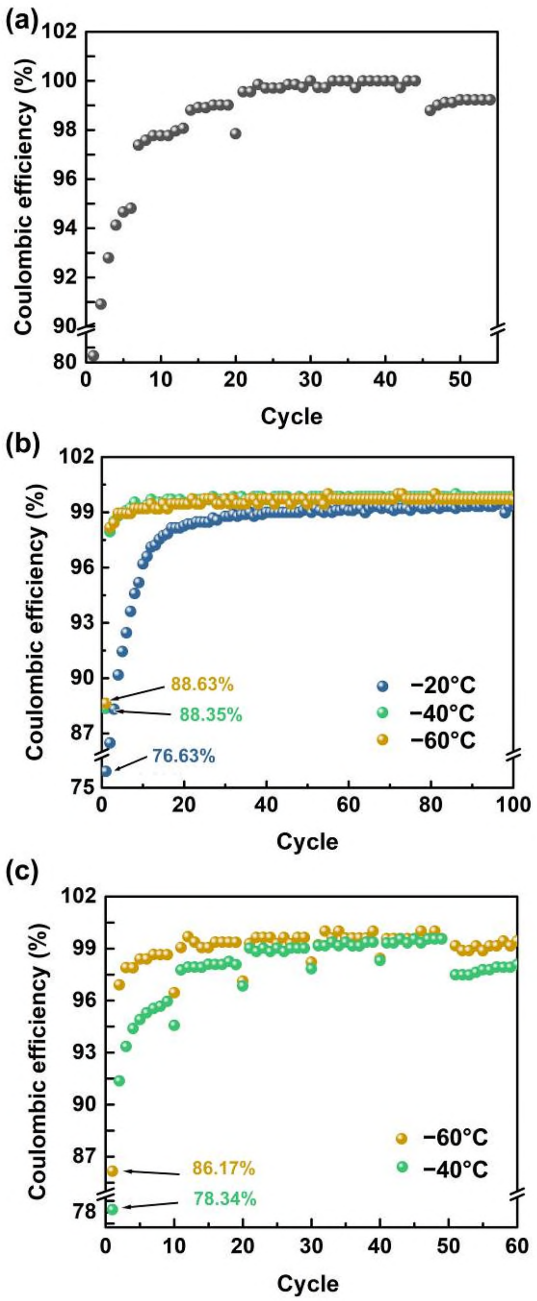

Supplementary Figure 43 | Coulombic efficiency of Na||$Na_3V_2(PO_4)_3$ coin cells using the 0.5M NaOTf-DEGDME/DOL (2:8) electrolyte solution at low temperatures. **a,** Coulombic efficiency of temperature-dependent galvanostatic cycling of cells at 22 mA g^{-1} (based on the active material of $Na_3V_2(PO_4)_3$) down to -60˚C with voltage cutoffs of 2.3 and 3.8 V in **Figure 6a**. **b**, Enlarged Coulombic efficiency of galvanostatic cycling of cells at 22 mA g^{-1} at −20˚C, −40˚C and −60˚C in **Figure 6c**. **c,** Coulombic efficiency of rate cycling performance (up to 110 mA g^{-1}) of cells at −40˚C and −60˚C in **Figure 6d**.

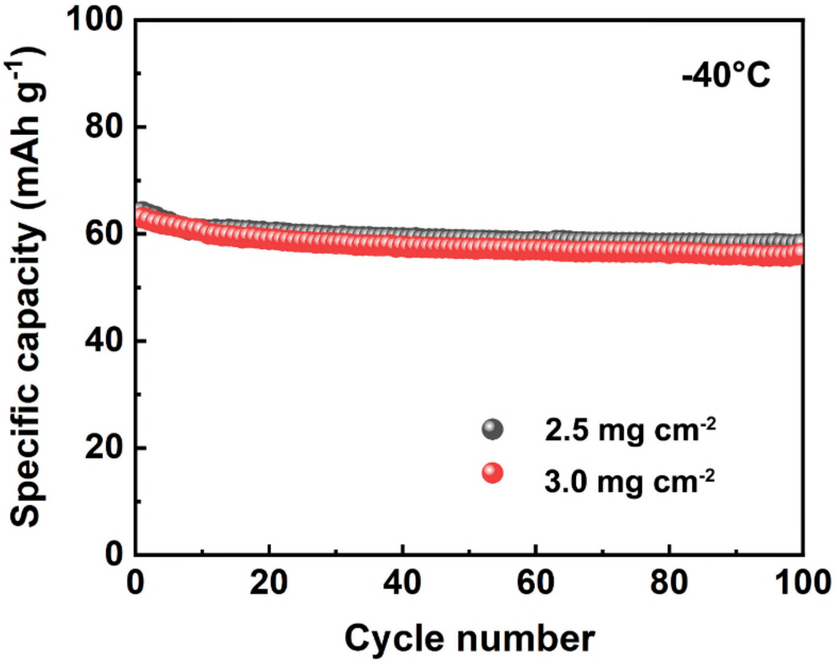

Supplementary Figure 44 | Cycling performance of Na||$Na_3V_2(PO_4)_3$ coin cells at different mass loadings of $Na_3V_2(PO_4)_3$ (up to 3.0 mg cm^{-2}) at 22 mA g^{-1} and −40˚C.

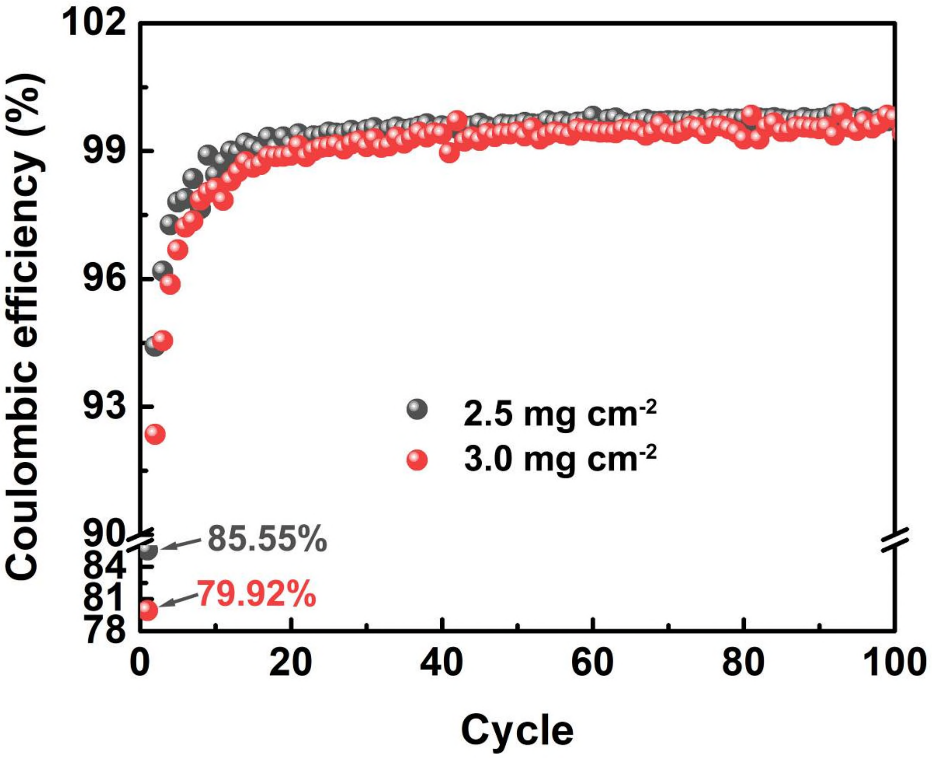

Supplementary Figure 45 | Coulombic efficiency of Na||$Na_3V_2(PO_4)_3$ coin cells at different mass loadings of $Na_3V_2(PO_4)_3$ (up to 3.0 mg cm^{-2}) at −40˚C reported in **Supplementary Figure 44**.

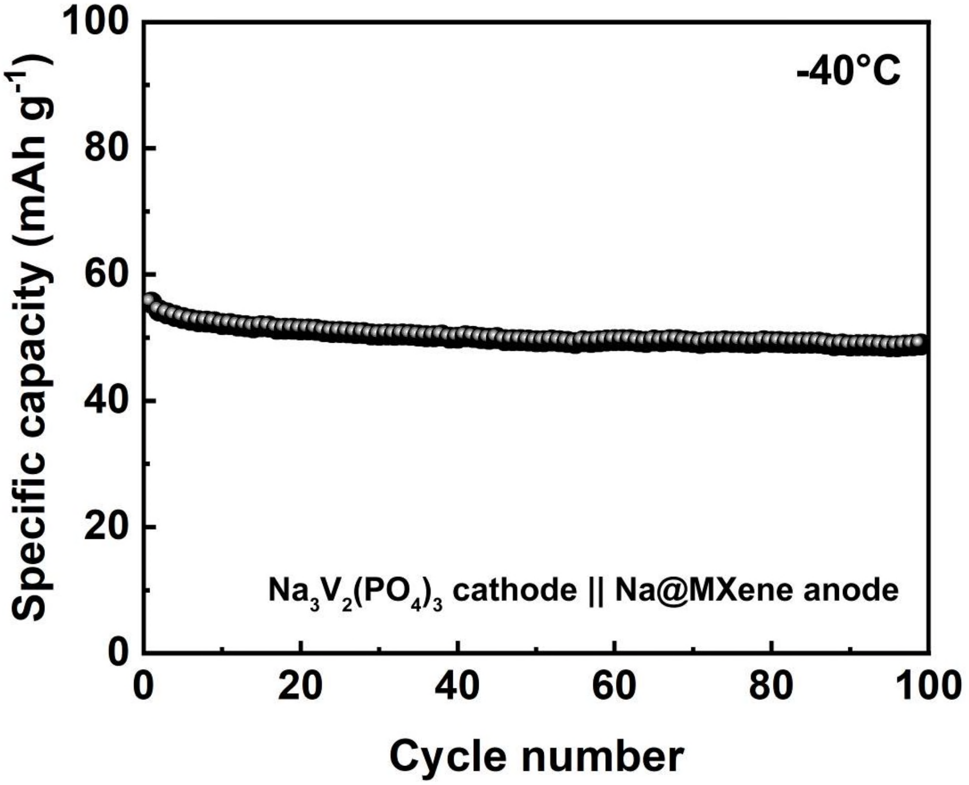

Supplementary Figure 46 | Galvanostatic cycling of full cells of $Na_3V_2(PO_4)_3$ cathode||Na@MXene anode at 22 mA g^{-1} and −40˚C**.**

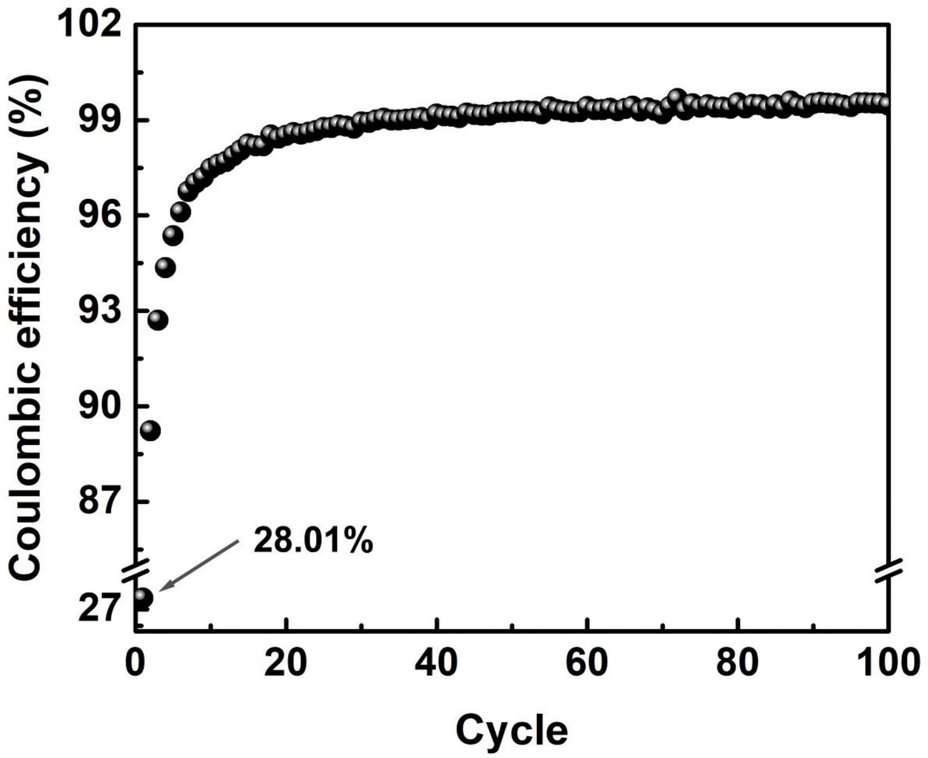

Supplementary Figure 47 | Coulombic efficiency of full cells of $Na_3V_2(PO_4)_3$ cathode||Na@MXene anode at −40˚C reported in **Supplementary Figure 46.**

Supplementary Table 1 | Summary and comparison of the electrochemical cycling performance at temperatures ≤ −40°C of various symmetric and asymmetric cells comprising Na and Li metal electrodes[1-4]. N/A indicates no long-term cycling reported.

Cell configuration	Temperature (ºC)	Current (mA cm^{-2})	Capacity (mAh cm^{-2})	Over-potential (mV)	Cycle number	Electrolyte	Ref
Na‖Na (coin cells)	−40	1.0	1.0	~100 (Average)	>165	1 M NaOTf-DEGDME (40 µL)	This work
Na‖Na (coin cells)	−80	0.5	0.25	~150 (Average)	>715	0.5 M NaOTf-DEGDME/DOL (2:8) (40 µL)	This work
Na‖Na (coin cells)	−80	0.2	0.1	~50 (Average)	>3,900	0.5 M NaOTf-DEGDME/DOL (2:8) (40 µL)	This work
Li‖Cu (coin cells)	−40	0.5	0.5	~150 (1st cycle)	N/A	1 M $LiPF_6$-PC/FEC (8:1) (15 µL mAh^{-1})	1
Li‖Cu (coin cells)	−60	0.2	0.5	~250 (1st cycle)	N/A	1 M $LiPF_6$-PC/FEC (8:1) (15 µL mAh^{-1})	1
Li‖Li (coin cells)	−40	0.5	0.25	~500 (Average)	10	0.8 M LiTFSI/ 0.2 M $LiNO_3$ - DOL/DME (8:2) + 10 vol % FEC (40 µL)	2
Li‖Li (coin cells)	−60	0.5	0.25	~1,000 (Average)	10	0.8 M LiTFSI/ 0.2 M $LiNO_3$ - DOL/DME (8:2) + 10 vol % FEC (40 µL)	2
Li‖Li (coin cells)	−80	0.2	0.1	~300 (Average)	10	1 M LiTFSI - DOL/DME (2:8) (40 µL)	3
Na‖Na (coin cells)	−40	0.5	1.0	~40 (Average)	5	1 M $NaPF_6$ - ether–[C_4C1im][BF4] (10 µL cm^{-2})	4

Supplementary Table 2 | Physicochemical properties of the electrolyte solvents investigated.

Solvent	Melting point (°C)	Dielectric constant (ε) at +25°C	Dynamic viscosity (η) (mPa·S) at −35°C
**DEGDME**	−64	7.30	2.34
**DME**	−58	7.20	1.89
**DOL**	−95	7.00	1.82

Supplementary Table 3 | Summary of the dissolution of five Na salts (salt concentration was kept at 1 M) in three different solvents, respectively, at −35°C (“Yes” indicates fully dissolved, “No” indicates not fully dissolved).

	**$NaPF_6$**	**NaOTf**	**$NaClO_4$**	**NaFSI**	**NaTFSI**
**Diglyme**	No	Yes	Yes	Yes	Yes
**DME**	No	No	Yes	Yes	Yes
**DOL**	No	No	No	No	Yes

Supplementary Table 4 | Summary of element values (R_s, R_{SEI}, and $R_{electrode}$) obtained from fitting the impedance data of Na||Na symmetric cells containing 1 M $NaPF_6$-DEGDME solution at 20˚C and -20˚C shown in **Supplementary Figure 2a and 2b** using the equivalent electrical circuits (insets in **Supplementary Figure 2a and 2b**).

	**20°C**		**−20°C**	
	**Value**	**Deviation**	**Value**	**Deviation**
**R_s (Ohm)**	4.239	0.471	38.75	0.2613
**R_{SEI} (Ohm)**	0.8716	0.5392	27.99	4.272
**$R_{electrode}$ (Ohm)**	5.843	0.1823	63.32	4.255

Note: Fitting of electrochemical impedance data was made using the frequency of 123 kHz to 0.07 HZ for -20°C; Fitting of electrochemical impedance data was made using the frequency of 123 kHz to 0.13 HZ for 20°C.

Supplementary Table 5 | Summary of resistance values (R_e) obtained from fitting the impedance data of stainless steel||stainless steel symmetric cells containing 1 M $NaPF_6$-DEGDME solution at a range of temperatures shown in **Supplementary Figure 2c** using the equivalent electrical circuit (inset of **Supplementary Figure 2c**).

	**R_e (Ohm)**	
	**Value**	**Deviation**
**20°C**	2.691	0.2522
**10°C**	3.149	0.2536
**0°C**	3.763	0.2515
**−10°C**	4.79	0.2514
**−20°C**	6.237	0.2511
**−30°C**	9.647	0.2519
**−35°C**	19.87	0.2571
**−40°C**	35.93	0.2582
**−45°C**	47.9	0.2587

Note: Fitting of electrochemical impedance data was made using the frequency of 155 kHz to 57 HZ.

Supplementary Table 6 | Summary of electrochemical behavior of single-solvent electrolyte systems (salt concentration was kept at 1 M) at 20°C.

	**$NaPF_6$**	**NaOTf**	**$NaClO_4$**	**NaFSI**	**NaTFSI**
**DEGDME**					
**DME**					
**DOL**					

Note:

Green color: stable voltage profile with low overpotential

Blue color: no testing due to salt precipitation at low temperatures

Orange color: unstable voltage profiles

Red color: very high overpotential

Supplementary Table 7 | Summary of identified XPS peaks[9-18].

C 1*s* (eV)	O 1*s* (eV)	F 1*s* (eV)	S 2*p* (eV)	Cl 2*p* (eV)	N 1*s* (eV)
$-CF_3$ (293.4)<br>$-CF_2$ (292.3)<br>Polycarbonate (289.7)<br>CHF (289.3)<br>O-C=O / CO_3^{2-} (288.7)<br>C-Cl (287.6)<br>$C-SO_2$ (287.2)<br>C-O-C (286.6)<br>C-C (284.8)<br>ClO_4^-, 1s (279.6)<br>ClO_3^-, 1s (277.9)	Na Kll (536.3)<br>CH_3-O- / $-CH_2$-O- (533.3)<br>C-O-Na (531.2)<br>Na_2O (528.2)	C-F (689.3)<br>S-F (687.8)<br>Na-F (684.1)	$F-SO_2$ (170.2)<br>SO_4^{2-} (169.6)<br>CF_x-SO_2 (168.9)<br>$S_2O_3^{2-}$ (168.7)<br>SO_3^{2-} (167.2)<br>S^{2-} (161.1)	ClO_4^- (208.5)<br>ClO_3^- (206.3)<br>ClO_2^- (203.8)<br>Cl^- (198.4)	Na-NN*N*, 1N (403.6)<br>S-N (399.3)<br>Na-N*NN*, 2N (398.5)<br>Na-C≡N (397.4)

NOTE: XPS positions in S 2*p* and Cl 2*p* spectra are based on $2p_{3/2}$. For XPS peak-fitting of S 2*p*, $2p_{3/2}$ to $2p_{1/2}$ area ratio is fixed at 2:1 and 1.18 eV is employed as the doublet separation of $2p_{3/2}$ and $2p_{1/2}$. For XPS peak-fitting for Cl 2*p*, $2p_{3/2}$ to $2p_{1/2}$ area ratio is fixed at 2:1 and the doublet separation of $2p_{3/2}$ and $2p_{1/2}$ is 1.60 eV.

Supplementary Table 8 | Summary of SEI composition (surface and bulk)[9-18].

	+20°C		−20°C	
**Electrolytes**	**Surface (<1 nm)**	**Bulk (2-15 nm)**	**Surface (<1 nm)**	**Bulk (2-15 nm)**
**1 M NaOTf-DEGDME**	$-CF_3$ containing components, Na_2SO_4, Na_2SO_3, NaF, Na_2CO_3, C-O-Na, organic debris	Na_2S (appear)	$-CF_3$ containing components, Na_2SO_4, NaF, Na_2CO_3, C-O-Na, organic debris	Na_2SO_3 (appear)
**1 M $NaClO_4$-DME**	NaCl, Na_2CO_3, C-O-Na, organic debris	N/A	$NaClO_4$, $NaClO_3$,C-O-Na, organic debris	$NaClO_2$ (appear)
**1 M NaFSI-DME**	$-SO_2F$ containing components, $Na_2S_2O_3$, NaF, C-O-Na, organic debris	Na_2S (appear)	$-SO_2F$ containing components, $Na_2S_2O_3$, Na_2SO_3, NaF, C-O-Na, organic debris	Na_2S, Na_2O, NaN_3 (appear); Na_2SO_3 (disappear)
**1 M NaTFSI-DEGDME**	$-CF_2$ containing components, Na_2SO_3, Na_2CO_3, Na_2S, NaF, NaCN, C-O-Na, organic debris	$-CF_2$ containing components (disappear)	$-CF_2$ containing components, Na_2SO_3, Na_2CO_3, Na_2S, NaF, NaCN, C-O-Na, organic debris	$-CF_2$ containing components (disappear)

Note: "appear" indicates that a component is not detected on the surface but observed in the bulk; "disappear" indicates that a component is shown on the surface but not in the bulk; "N/A" indicates that same components are observed on the surface and in the bulk.

Supplementary Table 9 | Summary of calculated reduction potentials of the solvents and salt species with or without the Na cation on the calculation (Units in V vs. Na/Na^+).

	DME	DEGDME	DOL	NaOTf	$NaPF_6$	$NaClO_4$	NaTFSI	NaFSI
**Without Na^+**	-1.15	-1.28	-1.06	-0.04	-0.50	-0.32	0.12	1.34
**With Na^+**	-0.02	0.25	-0.59	1.02	0.75	4.95	-0.30	2.37

Supplementary Table 10 | Summary of salt dissolution in binary solvents at 1 M/0.5 M concentrations at −35°C ("Yes" indicates fully dissolved, "No" indicates not fully dissolved, "N/A" indicates no results or failure in previous screening).

	**NaOTf (1 M)**	**NaOTf (0.5 M)**
**DEGDME-DOL (8:2)**	Yes	Yes
**DEGDME-DOL (5:5)**	Yes	Yes
**DEGDME-DOL (2:8)**	No	Yes
**DME-DOL (8:2)**	N/A	Yes
**DME-DOL (5:5)**	N/A	Yes
**DME-DOL (2:8)**	N/A	Yes

NOTE: The salt concentration below 0.5 M shows a marked decrease in conductivity, which makes such electrolytes not practical. Concentrations above 1 M cause significant salt precipitation at low temperature (e.g. −20°C)[19]. The salt concentrations between 0.5 M and 1 M are therefore chosen in the study.

Supplementary Table 11 | Summary of resistance values (R_e) obtained from fitting the impedance data of stainless steel||stainless steel symmetric cells containing 0.5 M NaOTf-DEGDME/DOL (2:8) solution at a range of temperatures in **Supplementary Figure 27a** using the equivalent electrical circuit (inset in **Supplementary Figure 27a**).

	**R_e (Ohm)**	
	**Value**	**Deviation**
**20°C**	11.6	0.2518
**0°C**	12.17	0.2465
**−10°C**	12.95	0.2513
**−20°C**	13.91	0.2435
**−30°C**	15.36	0.243
**−40°C**	17.26	0.2424
**−50°C**	20.13	0.2421
**−60°C**	24.29	0.2415
**−70°C**	30.6	0.2416
**−80°C**	41.36	0.2403

Note: Fitting of electrochemical impedance data was made using the frequency of 155 kHz to 57 HZ.

Supplementary Table 12 | Summary of resistance values (R_e) obtained from fitting the impedance data of stainless steel||stainless steel symmetric cells containing 0.5 M NaOTf-DEGDME/DOL (5:5) solution at a range of temperatures shown in **Supplementary Figure 27b** using the equivalent electrical circuit (inset in **Supplementary Figure 27b**).

	**R_e (Ohm)**	
	**Value**	**Deviation**
**20°C**	14.26	0.2512
**0°C**	16.03	0.2484
**−10°C**	18	0.248
**−20°C**	20.41	0.2469
**−30°C**	23.78	0.2404
**−40°C**	28.64	0.2393
**−50°C**	35.67	0.2333
**−60°C**	46.96	0.2325
**−70°C**	65.54	0.2322
**−80°C**	99.47	0.2532

Note: Fitting of electrochemical impedance data was made using the frequency of 155 kHz to 57 HZ.

Supplementary Table 13 | Summary of resistance values (R_e) obtained from fitting the impedance data of stainless steel||stainless steel symmetric cells containing 1 M NaOTf-DEGDME solution at a range of temperatures shown in **Supplementary Figure 27c and 27d** using the equivalent electrical circuits (insets in **Supplementary Figure 27c and 27d)**.

	**R_e (Ohm)**	
	**Value**	**Deviation**
**20°C**	8.371	0.2496
**0°C**	10.34	0.2478
**−10°C**	12.61	0.2482
**−20°C**	15.72	0.247
**−30°C**	20.4	0.2465
**−40°C**	27.78	0.2463
**−50°C**	40.44	0.2401
**−60°C**	65.4	0.2396
**−70°C**	120	0.2556
**−80°C**	265	0.255

Note: Fitting of electrochemical impedance data was made using the frequency of 155 kHz to 57 HZ.

Supplementary Note 1

Supplementary Figures 5 to **8** display the surface morphology of Na metal electrode cycled in different electrolytes at +20°C and −20°C. Besides the fact that the NaOTf-DEGDME electrolyte enables relatively smooth surfaces at both +20°C and −20°C. Key observations are summarized as below:

(1) There is distinct difference in the Na metal electrode surface morphology associated with $NaClO_4$ salt at +20°C and −20°C. For example, a severely damaged surface can be observed for $NaClO_4$-DEGDME at +20°C, while a relatively smooth surface (with pores identified from cross-section view) can be seen at −20°C. Similar trend can be observed for the case of $NaClO_4$-DME. This may be due to the significantly reduced reaction between the $NaClO_4$ salt and Na metal with decreasing temperature. This is also consistent with the electrochemical behavior investigation, in which the electrolyte with $NaClO_4$ salt operates much better at low-temperature compared to room-temperature condition.

(2) For the cases with NaFSI and NaTFSI salts, the electrolytes with NaFSI generally lead to less fractured and porous Na metal electrode surfaces compared to the ones with NaTFSI. Even though LiTFSI is reported as a frequently used salt for Li metal anode[20-23], NaTFSI is shown to be a less promising candidate for Na metal anode.

Supplementary Note 2

Supplementary Figures 13 to **21** present the XPS spectra, depth profiles and analyses on the chemical composition of the SEI formed in $NaClO_4$-DME, NaFSI-DME, and NaTFSI-DEGDME at +20°C and −20°C (four out of the eight electrolyte candidates were surveyed for the XPS analysis). Key analyses on the composition are summarized as below:

(1) For $NaClO_4$-DME, the composition of the SEI is very sensitive to temperature. Specifically, the NaCl (198.4 eV) component is ubiquitous at +20°C. In contrast, $NaClO_4$ (208.5 eV) and $NaClO_3$ (206.3 eV) are exhibited on the surface and $NaClO_2$ (203.8 eV) is present at the inner zone at −20°C. Note that the presence of ClO_4^- is possibly due to the precipitation/residual of the salt on the surface of Na metal. The detection of ClO_3^- is possibly due to the decomposition of $NaClO_4$. Such differences suggest that a lower temperature can alleviate severe corrosion caused by the reduction from $NaClO_4$ to NaCl.

(2) As for NaFSI-DME, the SEI formed at −20°C shows more complicated components in comparison to that formed at +20°C. At +20°C, -SO_2F enriched species (170.2 eV) and $Na_2S_2O_3$ (168.7 eV) exist on the surface in addition to NaF (684.1 eV), C-O-Na (531.2 eV) and organic debris, while Na_2S (161.1 eV) appears in the bulk. At −20°C, Na_2SO_3 (167.2 eV), NaN_3 (403.6 and 398.5 eV) and Na_2O (528.2 eV) are detected in addition to the above ingredients. Interestingly, the low temperature does not seem to reduce the extent of the reactions.

(3) With respect to NaTFSI-DEGDME, given similarity in the anion structure of $TFSI^-$ compared to FSI^-, the SEI species are moderately dissimilar. In particular, -CF_2 containing compounds (292.3 eV) and NaCN (397.4 eV) are revealed instead of -SO_2F enriched ones for the FSI^-.

References

[1] Gao, Y. et al. Low-temperature and high-rate-charging lithium metal batteries enabled by an electrochemically active monolayer-regulated interface. *Nat. Energy* **5**, 534–542 (2020)

[2] Thenuwara, A. et al. Efficient low-temperature cycling of lithium metal anodes by tailoring the solid-electrolyte interphase. *ACS Energy Lett.* **5**, 2411-2420 (2020).

[3] Thenuwara, A., Shetty, P. & McDowell, M. Distinct nanoscale interphases and morphology of lithium metal electrodes operating at low temperatures. *Nano Lett.* **19**, 8664–8672 (2019).

[4] Hu, X. et al. Deeply cycled sodium metal anodes at low temperature and in lean electrolyte conditions. *Angew. Chem.* **133**, 2–8 (2021).

[5] Saito, M. et al. Effects of Li salt anions and O_2 gas on Li dissolution/deposition behavior at Li metal negative electrode for non-aqueous Li-air batteries. *J. Electrochem. Soc.* **164**, A2872–A2880 (2017).

[6] Choi, W., Shin, H.-C., Kim, J., Choi, J.-Y. & Yoon, W.-S. Modeling and applications of electrochemical impedance spectroscopy (EIS) for lithium-ion batteries. *J. Electrochem. Sci. Technol.* **11**, 1–13 (2020).

[7] Raccichini, R., Furness, L., Dibden, J., Owen, J. & García-Araez, N. Impedance characterization of the transport properties of electrolytes contained within porous electrodes and separators useful for Li-S batteries. *J. Electrochem. Soc.* **165**, A2741-A2749 (2018).

[8] Gaberšček, M. Understanding Li-based battery materials via electrochemical impedance spectroscopy. *Nat. Commun.* **12**, 6513 (2021).

[9] Nazri, G.-A. &, Pistoia, G. *Lithium Batteries: Science and Technology*. Ch.17 (Springer, Press, 2003)

[10] Moulder, J. F. *Handbook of X-ray Photoelectron Spectroscopy: A Reference Book of Standard Spectra for Identification and Interpretation of XPS Data* (Physical Electronics Division, Perkin-Elmer Corporation Press, 1992).

[11] Nasybulin, E. et al. Effects of Electrolyte Salts on the Performance of Li–O_2 Batteries. *J. Phys. Chem. C* **117**, 2635−2645 (2013).

[12] Maibach, J. et al. A high pressure x-ray photoelectron spectroscopy experimental method for characterization of solid-liquid interfaces demonstrated with a Li-ion battery system. *Rev. Sci. Instrum.* **86**, 044101 (2015).

[13] Bodenes, L., Darwiche, A., Monconduit, L. & Martinez, H. The solid electrolyte interphase a key parameter of the high performance of Sb in sodium-ion batteries: comparative x-ray photoelectron spectroscopy study of Sb/Na-ion and Sb/Li-ion batteries. *J. Power Sources* **273**, 14-24 (2015).

[14] Aurbach, D. et al. On the surface chemical aspects of very high energy density, rechargeable Li-Sulfur batteries. *J. Electrochem. Soc.* **156**, A694-A702 (2009).

[15] Fiedler, C., Luerssen, B., Rohnke, M., Sann, J. & Janek, J. XPS and SIMS analysis of solid electrolyte interphases on lithium formed by ether-based electrolytes. *J. Electrochem. Soc.* **164**, A3742-A3749 (2017).

[16] Gu, Y. et al. Designable ultra-smooth ultra-thin solid-electrolyte interphases of three alkali metal anodes. *Nat. Commun.* **9**, 1339 (2018).

[17] Cao, X. et al. Monolithic solid–electrolyte interphases formed in fluorinated orthoformate-based electrolytes minimize Li depletion and pulverization. *Nat. Energy* **4**, 796-805 (2019).

[18] Lutz, L. et al. Role of electrolyte anions in the Na–O_2 battery: implications for NaO_2 solvation and the stability of the sodium solid electrolyte interphase in glyme ethers. *Chem. Mater*. **29**, 6066–6075 (2017).

[19] Lin, Y.-C. et al. Sputter-induced chemical transformation in oxoanions by combination of C_{60}^+ and Ar^+ ion beams analyzed with x-ray photoelectron spectrometry. *Analyst* **134**, 945–951 (2009).

[20] Miao, R. et al. Novel dual-salts electrolyte solution for dendrite-free lithium-metal based rechargeable batteries with high cycle reversibility. *J. Power Sources* **271**, 291-297 (2015).

[21] Qu, C. et al. $LiNO_3$-free electrolyte for Li-S battery: a solvent of choice with low K_{sp} of polysulfide and low dendrite of lithium. *Nano Energy* **39**, 262-272 (2017).

[22] Chen, X., Zhang, X.-Q., Li, H.-R. & Zhang, Q. Cation−solvent, cation−anion, and solvent−solvent interactions with electrolyte solvation in lithium batteries. *Batteries & Supercaps* **2**, 128 –131 (2019).

[23] Liu, Q. et al. Insight on lithium metal anode interphasial chemistry: reduction mechanism of cyclic ether solvent and SEI film formation. *Energy Storage Mater*. **17**, 366-373 (2019).
